# Characterization of Early-Phase Neutrophil Extracellular Traps in Urinary Tract Infections

**DOI:** 10.1371/journal.ppat.1006151

**Published:** 2017-01-27

**Authors:** Yanbao Yu, Keehwan Kwon, Tamara Tsitrin, Shiferaw Bekele, Patricia Sikorski, Karen E. Nelson, Rembert Pieper

**Affiliations:** The J. Craig Venter Institute, Rockville, MD, United States of America; Emory University School of Medicine, UNITED STATES

## Abstract

Neutrophils have an important role in the antimicrobial defense and resolution of urinary tract infections (UTIs). Our research suggests that a mechanism known as neutrophil extracellular trap (NET) formation is a defense strategy to combat pathogens that have invaded the urinary tract. A set of human urine specimens with very high neutrophil counts had microscopic evidence of cellular aggregation and lysis. Deoxyribonuclease I (DNase) treatment resulted in disaggregation of such structures, release of DNA fragments and a proteome enriched in histones and azurophilic granule effectors whose quantitative composition was similar to that of previously described *in vitro*-formed NETs. The effector proteins were further enriched in DNA-protein complexes isolated in native PAGE gels. Immunofluorescence microscopy revealed a flattened morphology of neutrophils associated with decondensed chromatin, remnants of granules in the cell periphery, and myeloperoxidase co-localized with extracellular DNA, features consistent with early-phase NETs. Nuclear staining revealed that a considerable fraction of bacterial cells in these structures were dead. The proteomes of two pathogens, Staphylococcus aureus and Escherichia coli, were indicative of adaptive responses to early-phase NETs, specifically the release of virulence factors and arrest of ribosomal protein synthesis. Finally, we discovered patterns of proteolysis consistent with widespread cleavage of proteins by neutrophil elastase, proteinase 3 and cathepsin G and evidence of citrullination in many nuclear proteins.

## Author Summary

Urinary tract infections (UTIs) are one of the world’s most widespread infectious diseases, with an estimated number of 150 million cases per year. Neutrophils play an important role in the defense of human patients against microbes causing UTIs. Molecules produced by neutrophils that migrate into the urinary tract can kill the invading microbes and resolve an infection, often without a need to treat patients with an antibiotic. Our work shows strong support for a mechanism called the formation of neutrophil extracellular traps (NETs), previously described for other infections and autoimmune conditions, which are involved in killing pathogens that have invaded the urinary tract. We show evidence of extracellular chromatin-containing structures using immunofluorescence microscopy and identified proteins that bind to the chromatin DNA and have functions to damage and kill bacterial cells or stop their growth.

## Introduction

Urinary tract infections (UTIs) are common bacterial infections estimated to cause disease in 150 million patients globally per year [1]. They are classified as uncomplicated when they affect the lower urinary tract, are not linked to structural and functional urinary tract abnormalities or catheterization, and occur in immunocompetent hosts [2]. All other UTIs which frequently occur in hospitals and long-term care centers are classified as complicated and have increased risks of pyelonephritis and urosepsis [1,2]. Typical guidelines for UTI diagnosis include a minimum of 10^5^ colony-forming units (cfus) per ml urine and symptoms such as high urgency or frequency of urination and abdominal or pelvic discomfort [2]. The most frequent cause of UTI is uropathogenic Escherichia coli (UPEC), which accounts for 74% of the cases among ambulatory care patients [2]. Pathogens have adapted to form biofilms on urethral catheters. Many, such as Klebsiella pneumoniae, Pseudomonas aeruginosa, Proteus mirabilis and Enterococcus species, are resistant to killing by normal sanitary measures and cause UTIs in the nosocomial environment [2,3]. The high rates of antibiotic prescription to treat UTIs and widespread acquisition of antibiotic resistance genes by the aforementioned pathogens have added to the concern of emerging bacterial strains that are no longer susceptible to any major class of antibiotic drugs [2].

Most UTIs are ascending infections that require the pathogen’s adherence to urothelial cells. Various types of fimbriae are critically important to the adherence of UPEC and P. mirabilis cells to urothelial surface receptors. These include CD11, CD44, uroplakins, and distinct glycosphingolipids, the latter of which activate urothelial cells via Toll-like receptor 4 (TLR4) [4,5]. When lipopolysaccharide (LPS)-binding proteins recognize bacterial LPS surface molecules, they also engage TLR4 and initiate signaling pathways that activate the transcription factor NF-kB. The result is the eventual secretion of chemokines and antimicrobial peptides [4,5]. Infected urothelial cells also express CXCR1, a surface receptor binding the chemokine CXCL8. CXCL8 and the interleukins IL-6 and IL-8 are considered important chemo-attractants for neutrophils and facilitate migration of the latter into the urothelial tissue [4,5]. Macrophages with distinct functionalities also recognize pathogens colonizing the urinary tract and promote the recruitment of neutrophils to the site of invasion [6]. Neutrophils are crucial effector cells for the innate immune response and the main determinants of the pathophysiology of pyuria [3,4]. There is some evidence that UPEC forms bacterial communities inside human urothelial cells, allowing evasion from neutrophil phagocytosis, a process that is potentially linked to recurrence of UTI [7].

Neutrophils are important as the first line of defense against invading pathogens. As recently reviewed [8], their antimicrobial strategies include phagocytosis, degranulation, and the formation of extracellular neutrophil traps (NETs). In the order listed, the strategies require increased amounts of time to respond to the microbial insults. Neutrophil granules have important functional roles in all three processes and are classified into azurophilic, specific, and gelatinase granules, each of which has distinct protein contents. The granules form sequentially during cell maturation, and the time-dependent release of their contents into phagosomes and the extracellular environment influences the inflammatory response overall, including the recruitment of other immune cells [9,10]. During the formation of NETs chromatin scaffolds are released as neutrophil nuclear and plasma membranes disintegrate [11]. The scaffolds are capable of containing the invading microbial cells while minimizing host cell damage. Immunofluorescence microscopy (IF) experiments have revealed that the DNA scaffold is dotted with histones, granular proteins such as myeloperoxidase (MPO) and elastase (NE), and the cytosolic calprotectin proteins S100-A8 and S100-A9 [12–14]. The proteins have direct antimicrobial and pro-inflammatory activities. The generation of reactive oxygen species (ROS) produced by NADPH oxidase is also implicated in the NET formation process and thus synergizes in the killing of the trapped pathogens. [12]. NE is important in early and intermediate stages of NET formation. At an early stage, H_2_O_2_ release triggers the dissociation of a complex in azurophilic granule membranes consisting of several effector proteins followed by the translocation of NE into the cytoplasm. Here, the enzyme binds to, and degrades, the F-actin cytoskeleton [15]. NE is freed to translocate into the nucleus where it degrades several core histones and triggers the decondensation of chromatin [13].

Pathogenic Streptococci and Staphylococcus aureus have evolved mechanisms to escape from NETs, in particular via the secretion of nucleases capable of degrading the extracellular DNA scaffold [16–18]. The formation of NETs has not been previously determined in the context of UTIs. Recently, we surveyed patient immune responses in more than 100 cases of UTI and asymptomatic bacteriuria via proteomics and found that urine sediment proteomes were rich in neutrophil contents [19,20]. Here, we examined such urine sediment samples further to assess the occurrence of necrosis and formation of NETs. Newly gained knowledge may lead to insights into the prevention of pathogen escape from neutrophil-associated defense strategies.

## Results

### Neutrophil-rich urine sediments form aggregates and are enriched for low M_r_ proteins

Leukocytes, in particular neutrophils, infiltrate the infected human urinary tract to eliminate invading pathogens. Proteomic analyses suggest that neutrophil proteins derived from urine sediments often exceed 50% of the total protein mass in UTI cases [19]. Cell-free urine fractions are also enriched in neutrophil-specific proteins in such cases [21]. This data indicates that phagocytosis is not the only strategy by which neutrophils combat uropathogens. Processes such as neutrophil degranulation, necrosis, and extracellular trap formation may play a role in the host defense. To examine whether any of these mechanisms are relevant in the context of UTIs, we examined 21 urinary pellet (UP) samples with large sediments, high leukocyte counts, and high neutrophil protein contents. Many of these UP samples revealed cellular aggregation prior to or after centrifugation at 1,500 x g and resuspension in PBS. From here on, we use the term aggregated UP (AUP) samples, most of which also had a viscous appearance. We refer to non-aggregated UP samples, which typically had lower leukocyte counts based on microscopy data, as easily dispersed UP (DUP) samples. Hematuria was not a factor in the extent to which the cellular aggregation phenotype was observed.

**Fig 1:**
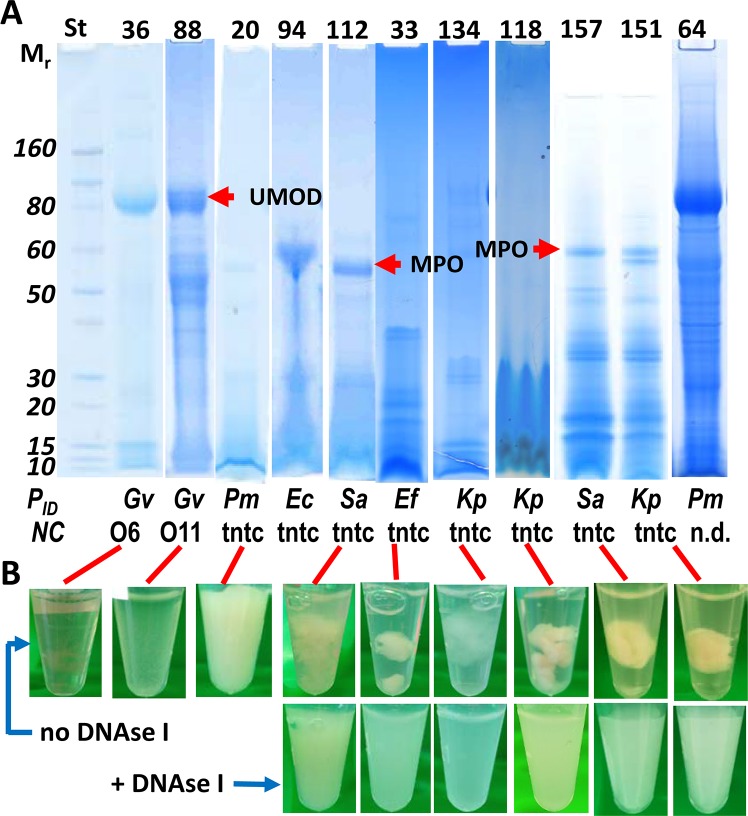
Urinary pellet lysates and solubilization of UP aggregates incubating with deoxyribonuclease I (DNase I). (A) SDS-PAGE gels with 4–12% acrylamide gradients were stained with Coomassie Brilliant Blue G-250. The left lane contains M_r_ standards. The protein extracts displayed here are #36, #88, and #64 (DUP samples), #20 and #118 (AUP samples with a phenotype showing complete or moderate loss of aggregation after 2 freeze-thaw cycles), and six AUP samples where DNase I treatment was required to homogenize the pellets under moderate agitation. The proteins UMOD and MPO marked in the gel image were identified by LC-MS/MS. The bars at the bottom show proteomic identifications (P_ID_) of microbial species and, if available, leukocyte counts/ml in the original urine sediments. Acronyms denote the following: *NC*, neutrophil counts in a high power field per ml urine; *tntc*, leukocytes too numerous to count; Gv, Gardnerella vaginalis; Pm, Proteus mirabilis; Ec, Escherichia coli; Sa, Staphylococcus aureus; Ef, Enterococcus faecalis; Kp, Klebsiella pneumoniae; *n*.*d*., not determined. (B) Photos of UP samples prior to and after incubation with DNase I in PBS at 37°C for 15 to 60 min.

AUP lysates analyzed in SDS-PAGE gels showed a decreased abundance of uromodulin (UMOD), an 80 kDa protein highly abundant in the uninflamed UP proteome, and intense protein bands in the M_r_ range lower than 20 kDa (Fig 1). This is in contrast to reports on protein profiles of resting and activated neutrophil lysates where broad distributions of M_r_ values were observed [22,23] and DUP sample profiles. In the latter, UMOD was frequently detected as a dominant SDS-PAGE band. Some AUP protein profiles featured a high M_r_ band, in the 55–60 kDa range, that was identified as the heavy chain of MPO by LC-MS/MS (Fig 1), thus supporting the occurrence of urinary tract infiltration by neutrophils. While several proteins highly abundant in activated neutrophils, e.g. calprotectin, which consists of the proteins S100-A8 and S100-A9, cathelicidin, α-defensin 1 and lysozyme (LYZ), naturally have low M_r_ values, the enrichment of low M_r_ bands in SDS-PAGE gels for AUP samples was indicative of intense proteolysis. Proteolytic processes occur during necrosis of neutrophils and formation of NETs [15,24]. As shown in Fig 1, analysis by LC-MS/MS and/or urine culture confirmed that AUP samples and some DUP samples contained proteins expressed by pathogens. Detailed data are provided in S1 Data.

### The AUP phenotype is associated with neutrophil disintegration

**Fig 2:**
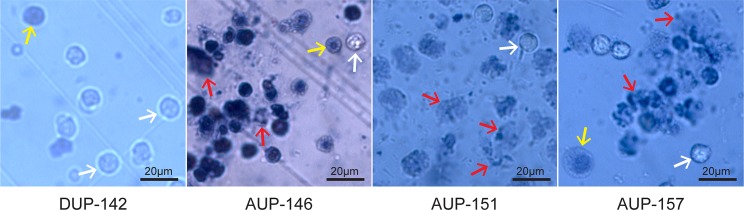
Evidence of neutrophil death, disintegration and multi-cellular aggregation in AUP samples. Urine sediment samples were centrifuged at 1,300 x g for 5 min and gently resuspended in PBS. Aliquots were stained with Trypan Blue (1:10 dilution) and subjected to phase-contrast microscopy (40-fold magnification). White arrows denote bright viable cells; yellow arrows denote dead cells, some with a flattened morphology and larger and some with visible nuclei stained blue; red arrows denote ruptured cells with membrane borders no longer clearly visible, some with dark patches of apparently released chromatin (especially in AUP samples #151 and #157).

To assess neutrophil viability in freshly collected urine samples (stored for maximally six hours at 4°C), their sediments were stained with Trypan Blue and inspected microscopically. AUP samples featured many dead cells and a high level of cellular disintegration and aggregation (Fig 2). DUP sample #142 showed some cell debris, but more viable neutrophils. While the image resolution was modest, microscopic data for samples #151 and #157 were consistent with the morphological changes reported for neutrophils undergoing NETosis [24]. Further experiments at the molecular level were necessary to determine whether UTI-associated neutrophil fates followed a path of necrosis or NETosis. Given that clinical samples were limited in availability and quantity and many experiments were time-sensitive, distinct experiments were performed on subsets of samples in this study. The table presented in S2 Data provides a list of experiments performed with 21 AUP and DUP samples.

### Proteins and DNA fragments are released by digestion of AUP samples with DNase

**Fig 3:**
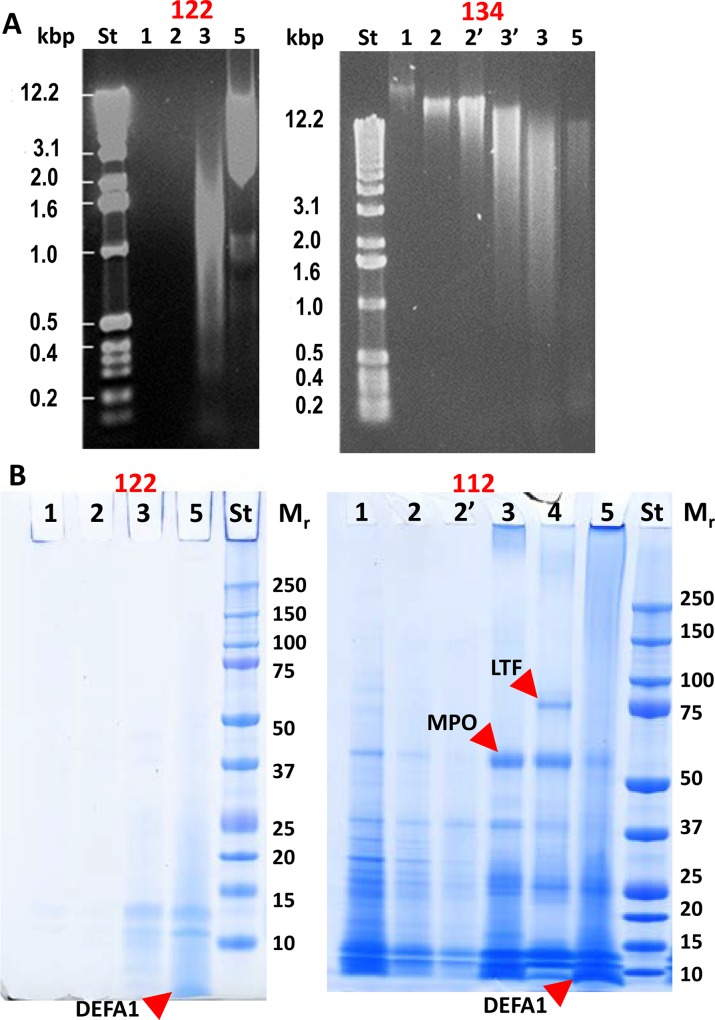
Sequential extraction of DNA and proteins from AUP samples. (A) Samples #122 and #134 were analyzed in 0.5% agarose gels and stained with ethidium bromide. DNA standards (St) are denoted in kilobase pairs (kbp). Lane numbers 1–5 match fraction numbers UP_sol_1, UP_sol_2, etc. (1 μl extract each). In the gel image for #134, lane 2’ pertains to a repeated incubation step with PBS and DTT, and lane 3’ pertains to a shorter 10 min incubation step with DNase I. UP_sol_3 (lane 3) represents incubation for 75 min. AUP sample #122 resisted disintegration of the pellet prior to the addition of DNase I; large size DNA was even retained even in fraction UP_sol_5. The aggregate in sample #134 released DNA in a decreasing size range with each incubation step. DNase I can cleave nucleic acids to nucleosome monomers (~ 0.2 kbp). (B) Protein extracts of samples #112 and #122 visualized in SDS-PAGE gels. Lane numbers 1 to 5 match fraction numbers UP_sol_1, UP_sol_2, etc. Ten μl extract were used in each lane. Low M_r_ proteins were abundant suggesting protein degradation in AUP samples. The positions of LTF, α-defensin 1, and MPO (all identified by LC-MS/MS) are marked in the gel image.

Similar to a method previously applied to characterize *in vitro* formation of NETs [14], we used incubations with DNase to determine whether extracellular DNA was present that could be degraded and afford the solubilization of proteins in AUP samples. In a previous report, neutrophil cultures stimulated to form NETs *in vitro* were shown to release abundant histones and azurophilic granule proteins, apparently bound to the DNA based on their alkaline p*I* values [14]. We expected the DNA digestion experiments, performed for 20 AUP and DUP samples, to discern NET-like structures from necrotic neutrophils. Proteins and DNA fragments were separated and visualized in gels, as shown in Fig 3 and S1 Data, following a sequential extraction process including PBS, PBS supplemented with 50 mM DTT, incubation with DNase, and detergent-mediated extraction and sonication to disintegrate residual cells, cytoskeletal and lipid membrane structures. In this order, the fractions UP_sol_1 to UP_sol_5 were generated. There was no measurable release of proteins after the DNase incubation from the DUP samples. In contrast, AUP samples released proteins into the fraction UP_sol_3. There was variation in the relative quantities of proteins in fraction UP_sol_3 compared to prior extraction steps, as shown for #112 and #122 in Fig 3. The release of DNA fragments following the incubation with DNase was a gradual process and stopped upon addition of the inhibitor Na-EDTA. While there was no visible release of DNA fragments prior to that incubation step in some cases (#122, Fig 3), other cases suggested that the AUP structures were fragile. Large DNA fragments were released from AUP sample #134 prior to the addition of DNase. Technical rather than biological reasons may account for this observation because several AUP samples were freeze-thawed prior to extraction (e.g., samples #20, #118, and #134). MPO and LTF, neutrophil granule effectors with high M_r_ values, were abundant in UP_sol_3 extracts in some cases (#112, Fig 3). The volume of insoluble matter retained after DNase incubation was markedly reduced for AUP samples, but not for DUP samples. Microscopic analysis of DNase-treated samples showed evidence of extensive cell degradation, but also of remaining intact neutrophils, thus supporting the notion that the DNase incubation itself did not lyse the cells.

Subsequent extraction steps (fractions UP_sol_4 and UP_sol_5) solubilized additional proteins. Calprotectin subunits (S100-A8, S100-A9) and α-defensin 1 were enriched in the low M_r_ range as shown in Fig 3. Why α-defensin 1 was so abundant in these extracts is unclear; the antimicrobial peptide is, however, known to bind to lipid membranes, which would explain the partitioning into a fraction with less soluble proteins. In summary, the extraction data provided preliminary evidence for structures consistent with the formation of NETs in AUP samples. Contributions from necrotic neutrophils to the release of DNA and proteins could not be ruled out based on the evidence that membranes in necrotic cells are likely permeable for enzymes such as DNase.

### LC-MS/MS analysis reveals protein profiles in UP_sol_3 fractions similar to *in vitro*-formed NETs

**Fig 4:**
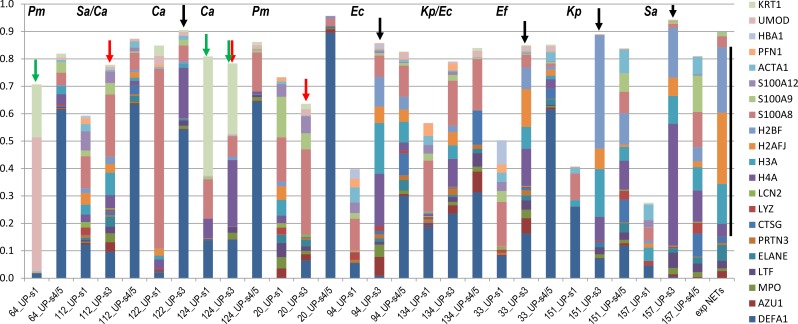
Protein profiles of UP_sol_ fractions show molecular evidence of NET formation in UTI cases. Proteomic quantification for UP_sol_3 fractions shows consistent similarities with profiles observed for *in vitro* generated NETs. Twenty-one highly abundant proteins were included in the graphic. Each colored segment in a stacked bar represents the relative amount of a protein in the total proteome of a fraction (including the UP_sol_1 and combined UP_sol_4/5 fractions; terms UP-s1, UP-s3, and UP-s4/5 are used on the x-axis). Each protein segment has a color corresponding to the color code displayed on the very right in the order of occurrence in the stacked bars. Protein names are UniProt short names. The bar displayed on the right (term ‘exp NETs’) represents the relative quantity of 13 proteins from a publication on *in vitro* generated NETs [14]. Black arrows on top of stacked bars denote samples in which the retention of insoluble DNA in UP fractions prior to the enzymatic digestion was high. Red arrows denote samples with partial release of DNA fragments prior to DNase incubation, which was in agreement with the gradual release of histones in the UP_sol_1 fraction. Green arrows denote samples with high cytokeratin (KRT1) and/or UMOD contents. The y-axis value of 1 represents 100% of the proteome using the proteomic quantification tool MaxQuant (all identified protein quantities are listed in S3 Data).

The degradation of DNA scaffolds from *in vitro*-generated NETs was previously shown to solubilize neutrophil effector proteins including histones [12–14]. We characterized UP_sol_3 fractions, in comparison with UP_sol_1 and UP_sol_4/5 fractions and *in vitro*-formed NETs, via shotgun proteomics. Ten AUP and five DUP samples were analyzed; data are shown for a subset of the analyses in Fig 4. All proteins found to be abundant in *in vitro* NETs [14] were identified, and often highly enriched, in the proteome of UP_sol_3 fractions derived from AUP samples. For the samples #94, #134, #33, #151, and #157 (Fig 4), the proteomic similarity to *in vitro* NETs was striking, especially when considering the fact that AUP samples also contain proteins soluble in urine and epithelial cell debris. Four histones, marked by the black line to the right of the stacked bar of *in vitro* (exp) NETs, and nine granule proteins located at the bottom in the stacked bar columns (Fig 4) contributed significantly to the overall UP_sol_3 proteomes. The data were consistent with an enzymatic process triggering protein release from extracellular chromatin. To demonstrate that the aggregation phenotype was not related to bacterial biofilms encased in a sticky extracellular matrix, the DUP sample #64 which was extracted from a urethral catheter biofilm was included here (Fig 4). Total protein release into the UP_sol_3 fraction was minimal. For sample #64, the twenty proteins dominating the proteomes of AUP samples and *in vitro*-formed NETs amounted to less than 5% to the total proteome of UP_sol_1 or UP_sol_4/5 fractions.

**Fig 5:**
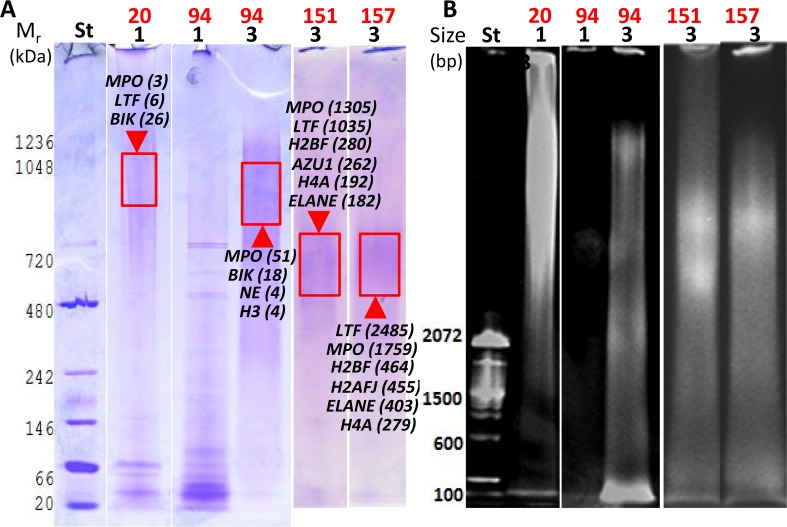
Analysis of DNA-protein complexes in solubilized extracts of four AUP samples using native PAGE. Non-denaturing 3% acrylamide gels were stained with CBB to visualize proteins **(A)** and ethidium bromide to visualize DNA **(B)** in the same gel. Stained areas with high M_r_ values (protein stain) and large sizes (DNA stain) match and are thus indicative of DNA-protein complexes. These areas were excised, digested with trypsin, and analyzed by LC-MS/MS. Proteins identified are denoted in the gel image with UniProt short names that were also used in Fig 4. BIK is the urinary protein bikunin. Lane and UP_sol_ fraction numbers listed below the sample ID (1 and 3) match. Sample #20 showed DNA release without adding DNase I into the fraction UP_sol_1. In contrast, the enzyme was responsible for the release of DNA fragments into the fraction UP_sol_3 for the samples #94, #151, and #157. The lack of DNA solubilization in prior extraction steps is shown for #94 (fraction UP_sol_1), and proteins in this fraction have generally lower M_r_ values forming sharper bands, consistent with the absence of association with DNA.

**Fig 6:**
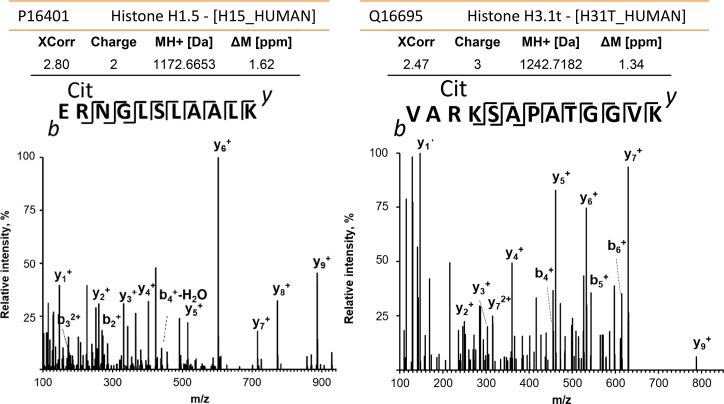
Citrullinated proteins in NET structures. Mass spectral data for the histone H1 peptide E_55_rNGLSLAALK_65_ (left) and the histone H3 peptide V_24_ArKSAPATGGVK_36_ (right), r = citrulline; m/z values for the y- and b-ion series confirmed R_56_ and R_26_ deamidations, respectively. Both spectra, derived from sample #112, were acquired using high-energy collisional dissociation MS/MS in the Orbitrap mass analyzer with a resolution of 17,500 and a mass accuracy of ~1 ppm. The human protein database was searched in the Proteome Discoverer software (v1.4) using a FDR ≤ 1%. The XCorr scores were 2.80 and 2.47 for the H1 and H3 peptide spectra, respectively. Analysis with the MaxQuant software tool confirmed the peptide citrullination sites.

Proteomic differences comparing the UP_sol_3 and UP_sol_1 fractions (for a given AUP sample) support the presence of an extracellular DNA scaffold that often remains intact until the digestion step with DNase and that mostly binds histones, particularly H4A, H3A, H2A and H2B, the cytosolic proteins S100-A8 and S100-A9, and the granule proteins MPO, LTF, NE, cathepsin G (CTSG), and azurocidin (AZU). In cases such as AUP sample #20 where DNA is released prior to DNase treatment (Fig 5) and freeze-thaw cycles appeared to have degraded the DNA scaffold, the aforementioned proteins were present in higher quantities in the fractions with disintegrating DNA. Fractions UP_sol_4 and UP_sol_5 revealed higher abundances for neutrophil plasma and granule membrane proteins such as stomatin, subunits of cytochrome b-245 which contribute to the NADPH oxidase complex, and subunits integrin αM and β2 which form neutrophil surface receptors stimulating chemotaxis. Nucleophosmin was fairly abundant in some UP_sol_3 fractions, suggesting that nuclear proteins other than histones remain bound to DNA during the process of NETosis. A specific biomarker of NETs is citrullinated histone H3 [25]. We identified histone H3 peptide V_24_ArKSAPATGGVK_36_ with a deamidated R_26_ residue that is a known citrullinated site [26] and three citrullinated histone H1 peptides (Fig 6). One peptide, E_55_rSGVSLAALK_65_, contained a deamidated R_56_ residue that was reported to regulate chromatin binding [27]. Interestingly, citrullinated peptides were also identified for two histone-binding phosphoproteins, nucleophosmin and acidic leucine-rich nuclear phosphoprotein 32 family member A, and several cytoskeletal proteins. The peptides were often identified in AUP samples (#112, #118, and #157) with additional lines of evidence for early-phase NET formation. A total of 32 mass spectra were consistent with peptides that had citrullination sites (S4 Data). A frequently identified arginine deiminase in AUP samples was PADI2 suggesting that this enzyme catalyzed the citrullination reactions. To summarize, the results pertaining to the specimen susceptibility to digestion with DNase, the release of protein effectors and evidence of citrullinated histones encouraged the notion that NETs were formed in AUP samples.

### Evidence of DNA-protein complexes in AUP extracts

To support the concept that proteins enriched in DNA-containing fractions bound to host genomic DNA in AUP extracts, we used non-denaturing PAGE analysis and evaluated gel regions simultaneously stained for DNA and protein. As shown in Fig 5, there was evidence of partially degraded DNA co-migrating with proteins, including fragile UP aggregates (UP_sol_1 fraction, sample #20) and more stable UP aggregates (UP_sol_3 fractions, samples #94, #151 and #157). The complexes migrated over a wide M_r_ range with the most intense DNA and protein staining in the 0.6–1.2 mDa range. Native PAGE often preserves macromolecular assemblies. For samples #151 and #157, more than 99% of the total protein extracted from the gel regions shown in Fig 5 consisted of antibacterial NET effector proteins. A protein unexpectedly identified by LC-MS/MS from the gel extracts of samples #20 and #94 was bikunin (BIK). BIK is a protease inhibitor with anti-inflammatory activity secreted into urine by tubular cells of the kidneys. The protein-DNA co-migration pattern was not observed for samples with low DNA content (UP_sol_1 fraction, #94; Fig 5). Well-resolved protein bands supported the notion that the observed complexes of protein and DNA did not result from unspecific protein retention in a high M_r_ range of the gels.

### Proteins in the NET-like structures undergo proteolytic processing

**Fig 7:**
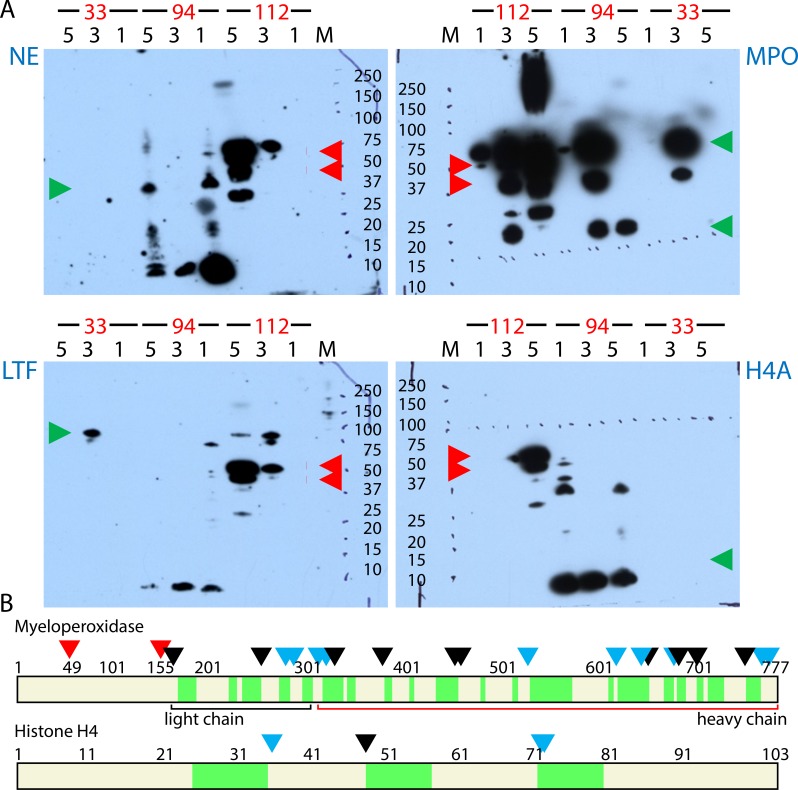
Proteolytic degradation in extracts of AUP samples. **(A)** Western Blots were performed with polyclonal antibodies specific for NE, MPO, LTF, and histone H4A. The lane numbers match fraction numbers (UP_sol_1, UP_sol_3, and UP_sol_5) derived from samples #33, #94, and #112. The M_r_ standard consists of ten proteins denoted with kDa values. Green arrowheads point to M_r_ values observed for full-length proteins, including the heavy and light chains of MPO. Full-length NE (29 kDa) and a H4A fragment (8–10 kDa) were detected only in fractions of sample #94. LTF and MPO were represented by full length protein bands in UP_sol_3 fractions. The S. aureus protein A (SpA) was detected in a M_r_ range corresponding to its post-translationally modified, cell wall-immobilized forms for sample #112 (red arrowheads). **(B)** Cleavage sites identified in the peptide sequences of MPO and H4A that were in agreement with the preferred P1 site specificities of the proteases NE and PRTN3. This data is deduced from peptide termini identified via LC-MS/MS from five AUP samples. The N- and C-termini of peptide segments shaded in green include experimentally generated trypsin-specific cleavage sites and PRTN3/NE-specific sites apparently formed as a consequence of the *in vivo* inflammatory process. Red arrowheads denote sequence positions resulting from protein maturation of precursors. Other arrowheads denote PRTN3 and NE cleavage sites (C-terminal to A, V, L, I, S, T, C, M); if colored black, the site was identified in three or more of the five examined AUP datasets.

Intense staining for low M_r_ proteins in many AUP samples suggested proteolytic degradation. Indeed, the main neutrophil granule proteases, ELANE, CTSG, and proteinase 3 (PRTN3), were among the 30 most abundant proteins in UP_sol_3 fractions. To detect evidence of proteolysis in fractions of AUP samples occurred, we performed western blots for MPO, LTF, histone H4A, and ELANE (NE). The protein least affected by proteolysis was MPO (Fig 7A), which is consistent with data presented in Fig 4. Heavy and light chains of MPO were detected with the predicted M_r_ values in western blots. High peroxidase activities in the UP_sol_3 fractions derived from the samples #94 and #112 and the UP_sol_1 fraction derived from sample #20 using 3,3′,5,5′-tetramethylbenzidine as the substrate in colorimetric assays confirmed that MPO was enzymatically active. LTF and NE were visualized with bands corresponding to full-length and truncated versions. Western blot bands for histone H4A were only detected for sample #94 with a M_r_ value of approximately 9 kDa (Fig 7A) suggesting partial degradation of histones, as reported for *in vitro*-NETs [13]. The *S*. *aureus* protein A (Spa) was detected as a full-length protein in immunoblots (sample #112, red arrowheads in Fig 7A). Spa binds to the heavy chain Fc region of IgG at the bacterial surface and interferes with the opsonization and phagocytosis of *S*. *aureus* cells. The activity of Spa is apparently retained despite the use of denaturing methods and a host environment with high protease activities. In the experiments shown, Spa bound to the anti-rabbit IgG antibody conjugate. Spa was identified in the same UP_sol_ fractions by LC-MS/MS. Immunoblots using purified Spa (0.1 μg) and lysates from two *S*. *aureus* strains (S1 Data) revealed bands that matched the M_r_ variants in the 40–50 kDa range. We hypothesize that, in infections with *S*. *aureus* where the immune response implicates NETosis, Spa binds to IgG and shields the bacterial surface from the binding of neutrophil effectors.

The activities of NE, PRTN3, and CTSG derived from *in vitro*-NETs were recently characterized by peptide profiling using synthetic peptide libraries [28]. With this information and the proteolytic events observed in western blots, we assessed LC-MS/MS data for the proteolysis in sequences of 41 proteins in AUP and DUP samples by NE or PRTN3. The range of peptide IDs in AUP samples, but not DUP samples, showed evidence of widespread proteolytic cleavage. The peptide site maps are provided in S5 Data. Large proteins such as MPO (Fig 7B), F-actin, actinin α-1 and gelsolin featured more NE/PRNT3 cleavage sites. Small proteins such as H4A (Fig 7B), H2B and profilin had fewer cleavage sites. Since the experimental approach included digestion with trypsin, an enzyme sharing the cleavage site C-terminal to K and R residues with CTSG, we could not detect CTSG-mediated proteolysis. All three proteases had peptide IDs consistent with their own cleavage by NE and PRTN3, potentially involving autolysis. Cytoskeletal proteins had a high number of cleavage sites. To verify that the probabilistic approaches used for peptide identification did not result in incorrect conclusions, we checked for peptide cleavage sites in precursor protein segments that are rapidly degraded to allow activation or targeted localization in the cell. Indeed, no peptides were detected for precursor regions including the N-terminal 163 amino acids of MPO. We identified the known PRNT3-specific cleavage site for cathelicidin (A_136_/L_137_) that results in the generation of the antimicrobial peptide LL-37 [29]. We identified the core sequence of LTF with antibacterial properties (F_171_-A_201_), termed kaolicin-1, although the peptide boundaries did not rule out trypsin cleavage. We conclude that protein degradation in AUP samples implicates major neutrophil proteases and is a widespread phenomenon.

### Peptidomic data suggest that proteolysis is not limited to AUP samples

**Fig 8:**
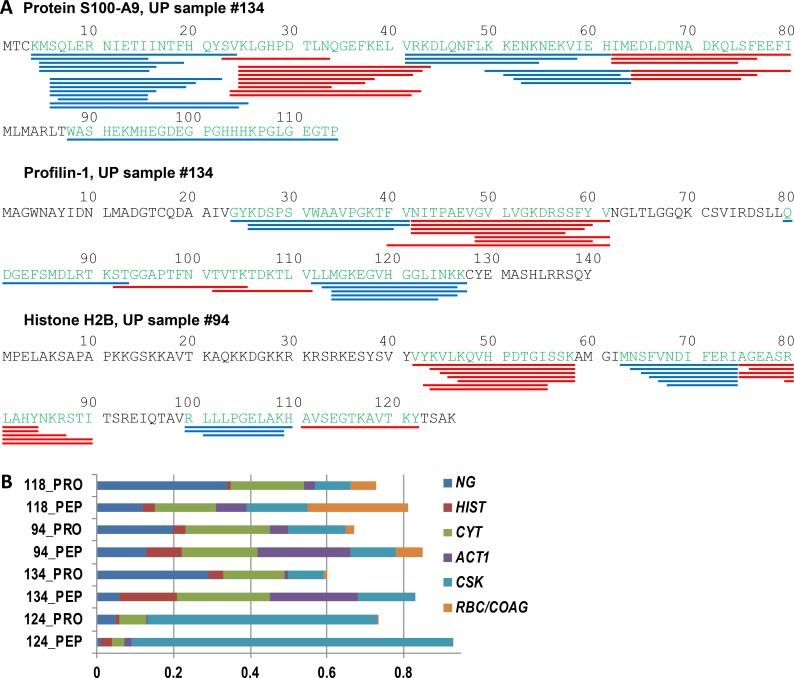
Proteins in AUP samples are degraded to the level of peptides consistent with activities of cathepsin G, proteinase 3 and, elastase. **(A)** Peptide maps for protein S100-A9, prolifin-1, and histone H2B. This data is derived from peptidome analyses combining the fractions UP_sol_1, UP_sol_2, and UP_sol_3 from both sample #94 and sample #134. No enzymatic cleavage sites were pre-selected in the database searches. The NE, PRTN3, and CTSG specific cleavage sites determined from peptide termini are mapped along each the respective protein sequence. Peptide termini not consistent with preferred cleavage sites of the three proteases were rare. The peptides, which are highlighted in the form of red and blue bars along the amino acid sequence to mark where they end, revealed peptide clusters around common cores. **(B)** Relative abundances of peptides associated with protein localization or functional groups comparing peptidome (PEP) and equivalent shotgun proteome (PROT) datasets. Quantification of peptides is based here on peptide-spectral counts. Protein groups denoted on the right of the graphic have color codes pertaining to the following names, functions, and localizations: NG, neutrophil granules; HIST, histones; CYT, cytosol; ACT1, actin; CSK, cytoskeleton (except actin) and keratins; RBC/COAG, red blood cells and coagulation.

We modified the technical approach to examine whether activities of NE, PRTN3, and CTSG are limited to the truncation of proteins to larger fragments rather than degradation to the level of oligopeptides. Analyzing peptide-enriched filtrates with a M_r_ cutoff of 10 kDa from six AUP samples without tryptic digestion, we found that up to 1,050 unique peptides with a length of 6 to 32 amino acids, assigned to 60 to 220 proteins, were identified. The frequency of small peptides derived from cytoskeletal proteins and histones, hemoglobin, likely derived from hematuria, and cytokeratin variants, likely derived from exfoliated urothelial cells, were particularly high in all AUP samples. The representation of neutrophil granule effector peptides was lower in peptidome datasets (S6 Data). Protein sequence maps with locations for the peptide IDs pertaining to the protein S100-A9, the cytoskeleton-associated protein profilin-1 and histone H2B are displayed in Fig 8A. The peptides clustered in distinct parts of the protein around a core peptide segment flanked by peptide overhangs varying in length. The data suggest that specific sites in the target protein are more susceptible to enzymatic cleavage than others. Greater than 98% of the peptide termini corresponded to the preferred P1 sites of CTSG (R, K, Y, and F) and of NE/PRTN3, again implicating these enzymes in such proteolytic events. The data are consistent with previous reports linking histone and actin degradation to NETosis [13,15]. We compared the peptide IDs derived from shotgun proteome and peptidome datasets for specific function- or localization-related protein groups as shown in Fig 8B. The analysis supported the notion that neutrophil granule proteins were less susceptible to degradation by CTSG, NE, and PRTN3 than other protein groups.

Peptidome data derived from DUP samples were also examined. Only a few peptides were identified from two DUP samples, while numerous peptides were identified from two other samples (#28 and #36). While the latter samples had peptide IDs consistent with lower neutrophil representation, histone and cytoskeletal protein-derived peptides were frequently observed. The findings suggest that the proteolysis in UP samples is not limited to those that have evidence of necrotic neutrophils or NETs. Furthermore, it highlights the possibility that a larger set of peptides is bio-activated in processes similar to those known for α-defensin and LL-37.

### Immunofluorescence microscopy data support formation of early-phase NETs in AUP samples

**Fig 9:**
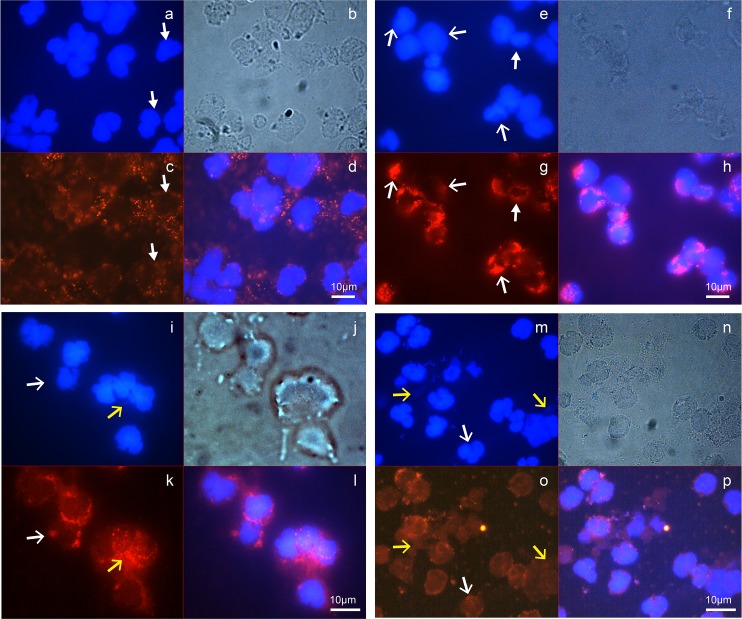
Detection of early-phase NETs by IF microscopy. AUP aliquots were paraformaldehyde-fixed on glass slides on the day of specimen collection and stored at 4°C until further use. IF staining was performed with an MPO-specific polyclonal antibody followed by an anti-rabbit IgG conjugate to the dye CFl-555, and counterstaining with DAPI. Oil immersion microscopy (not confocal) was used for imaging with phase contrast and in blue and red channels. (a-d) sample #142; (e-h) sample #146; (i-l) sample #151; (m-p) sample #157. Sample #142 shows evidence of lobulated nuclei and no evidence of extracellular chromatin release (a), intact granular structures and well-defined cell perimeters (b), MPO staining in accordance with intact granules (c), and no co-localized MPO/chromatin staining (d). Sample #146 has intact neutrophils, but also some cells where nuclei fill the entire cell space (e) and granules are diminished in the perimeter of cells according to staining for MPO (g). Co-localization of nuclei and MPO is visible in the cell perimeter suggesting the emerging loss of nuclear membranes (h). Sample #151 shows less regularly shaped nuclei with fainter staining in their perimeters suggesting nuclear membrane disintegration (i), and patchy granular staining as described above (k); MPO and nuclear staining with DAPI overlap (l). Sample #157 shows areas of flattened and disintegrating cells (m, n) and streaks of extracellular DNA (m) that co-localizes with MPO staining (o, p). The closed white arrows point to cells with intact nuclei and well-distributed cytoplasmic granules. Open white arrows point to cells filled with chromatin and a patchy staining pattern for granules (MPO). Open yellow arrows point to disintegrated cells releasing chromatin from nuclei that co-localizes with MPO.

To obtain cellular evidence for the presence of necrotic neutrophils and/or NETs in AUP samples, immunofluorescence experiments were performed. The flattened, rounded morphology of enlarged neutrophils present in freshly collected AUP samples was presented (Fig 2). DAPI staining consistent with decondensed chromatin and the loss of nuclear lobules is visible in samples #146, #151, and #157 (open white arrows, Fig 9E, 9I and 9M). MPO staining reveals a decreased number and distribution of granules in contrast to those of intact neutrophils in sample #142 (closed white arrows, Fig 9C). The nuclear membrane border is less distinct with chromatin released into the cytoplasmic space (samples #151 and 157) and visibly expelled chromatin in the extracellular space (#157). MPO co-localizes with chromatin (open yellow arrows, Fig 9I, 9K, 9M and 9O). We did not observe stretches of laminar DNA associated with bona fide NETs. Together with the biochemical experiments, IF data for samples where cell fixation was performed right after collection are consistent with early-phase NET structures. In Fig 9M and 9N, S. aureus cells are visualized as small blue circles (chromatin, DAPI stain) and bright circles of the same size (phase contrast), respectively, suggesting extracellular entrapment.

### Potential role of NETs in the immune defense towards pathogens in UTI context

**Fig 10:**
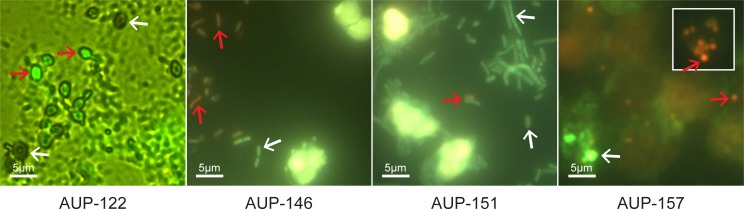
Microbial cell viability staining for infectious agents in AUP samples. Images are from oil immersion microscopy with the green (fluorescein) channel for sample #122, and green and red (rhodamine) channels for samples #146, #151 and #157. Image #122: AUP sample aliquot was incubated with SYTOX-green (5 μM in TBS) in the dark for 15 min, fixed on a glass slide at low heat (40°C), washed with water, and air-dried. C. albicans yeast forms are clearly visible. Red arrows point to dead cells (SYTOX-green stained), white arrows point to intact cells (unstained). Images #146 to #157: Sample aliquots were incubated with the live/dead differential staining kit (5 μM SYTO9 and 55 μM propidium iodide in TBS) in the dark for 15 min followed by centrifugation at 800 x g for 3 min, re-suspension in TBS, a 2^nd^ centrifugation step, and fixation with 4% paraformaldehyde for 15 min. #146: rod-shaped E. coli cells propidium iodide-stained (red arrow) are dead. #151: filamentous K. pneumoniae rods stained with SYTO9 are living cells (white arrow). Neutrophils (bright yellow stain) are surrounded by bacterial cells suggesting a failure of phagocytosis. #157: S. aureus cocci are trapped in NET-like structures with red and green dots indicating death and survival (red and white arrows, respectively). The #157 insert shows a cluster of dead bacterial cells.

Differentiating among neutrophil-based immune defense strategies that include degranulation, phagocytosis and NETs is difficult because the strategies largely rely on the same molecules that eventually kill invading pathogens or arrest their growth arrest. Neutrophil granule proteins and reactive oxygen species produced by MPO and NADPH oxidase play key roles. For several AUP samples we associate here with early-phase NET formation, we recovered few microbial colonies upon LB or BHI agar culture (#20, P. mirabilis; #33, E. faecalis; #122, C. albicans; #118 and #134, K. pneumoniae and E. coli). This suggests but does not prove killing by NETs. To assess bacterial viability in microscopy experiments, several samples were stained with a live/dead bacterial cell staining kit or SYTOX-green which stains only nuclei in damaged cells. The images for the samples #122, #146, and #157 (Fig 10) revealed more dead than living microbial cells, and the samples #122 and #157 had evidence of NET-like structures in which dead and living microbial cells were visible. In contrast, K. pneumoniae cells in the image for sample #151 were mostly viable and formed bacterial filaments that have previously been associated with subverting innate immune defenses by *E*. *coli* during UTI [30]. This data does not prove, but supports a contributing role of NETs in the defense against the uropathogens.

### S. aureus and E. coli proteomes: evidence of growth-arrested cells under attack by neutrophil effectors

**Fig 11:**
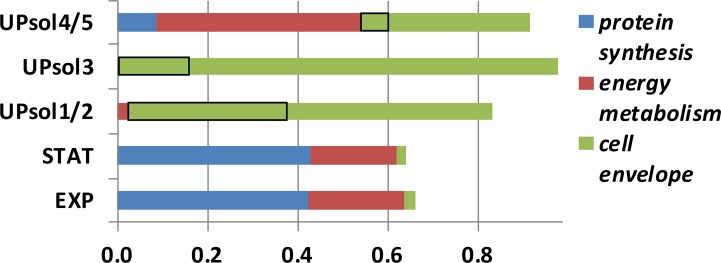
*S*. *aureus* proteome comparing *in vitro* cultures of the isolate from AUP sample #112 to *in vivo* data derived from sequential extraction of the clinical specimen. The UP_sol_ fractions were defined in the main text. The isolate was grown in LB media to mid-exponential (EXP) and stationary (STAT) phases. Protein quantification was based on MaxQuant analysis, and proteins were assigned to three groups based on their roles in ribosomal protein synthesis, energy metabolism and cell envelope localization as annotated in the database UniProt. Black-rimmed boxes are the combined quantities of the cell wall enzymes Atl and IsaA. Detailed bacterial protein profiles are provided in S7 Data.

Analyzing the bacterial proteome from AUP samples (*in vivo*), compared to their growth to mid-exponential or stationary phase in culture (*in vitro*), provides information on the adaptation to the hostile host environment associated with the early-phase NETs and activated neutrophils in general. We analyzed the proteome from samples #94 (E. coli) and #112 (S. aureus). For AUP sample #112, the survey included the cell-free fractions (UP_sol_1/2 and UP_sol_3) and those that contained cells and cell debris (UP_sol_4/5). The cell-free fractions were markedly increased for S. aureus cell envelope proteins including the autolysin Atl and the transglycosylase IsaA, which made up more than 17% of the entire proteome (Fig 11). Other cell wall-localized proteins were also identified, including the aforementioned Spa protein, the SsaA antigen, proteins adhering to neutrophil, endothelial, and epithelial cell surfaces (Eap, Efb, IsdA, and IsdB), and transporters for metal ions and siderophores (IsdB, SirA, Cnt, and ZnuA). Two secreted virulence factors, the complement system inhibitor SCIN and hemolysin-γ, were also identified. Neutrophil protease activities and cell envelope damage may have directly triggered the release of these proteins from the bacterial cell. The autolysin Atl is involved in cell division, suggesting that its removal from the bacterial cell may have resulted in the arrest of cell division and growth. In fraction UP_sol_4/5, the contribution of cell envelope proteins to the proteome was lower, but low ribosome and translation-associated protein contents suggested markedly decreased ribosomal protein synthesis activity compared to *in vitro* grown S. aureus cells (Fig 11). This observation is consistent with the need to repair and regenerate cellular structures in bacteria facing neutrophil attack and the arrest of cell growth. Similarly decreased ribosomal protein synthesis was observed for E. coli in the sample #94.

## Discussion

Our analysis of AUP samples resulted in molecular and cellular support for the formation of early-phase NETs in the context of UTIs. Using specimens subjected to freeze-thaw cycles prior to experiments characterizing neutrophil defense strategies, it was evident that the structures in most AUP samples lost their aggregated consistency and were susceptible to disintegration. The fragility of NETs in the context of various pathologies was described [12,31,32]. Phase contrast microscopy for a set of freshly collected samples revealed the presence of aggregates of mostly non-viable and lysed neutrophils consistent with necrosis or NETosis. Visualization by IF using DAPI staining showed that some but not all neutrophils had a flattened, rounded morphology. Chromatin filled the entire cellular space with no distinction between euchromatin and heterochromatin. These feature are consistent with necrotic neutrophils [24] and early-phase NETs [12,32]. Visualization by IF with an MPO antibody supported the notion that NET-like structures had formed. In clusters of disintegrating neutrophils, extracellular chromatin streaks that co-localized with MPO were visible, especially for sample #157 (Fig 9). Together, the morphological and DNA/protein co-localization traits are more in line with early-phase NETs than necrotic neutrophils [12,24]. The MPO staining pattern was different in samples with many viable neutrophils (#146, well distributed granules) compared to those dominated by dead and lysing neutrophils (#146, #151, #157; Fig 9). These samples revealed remnants of granules in the cell periphery and co-localized staining with chromatin. The caveat of the interpretations is that the images were not confocal, thus not permitting visualization in confocal slices. Unambiguous co-localization would require confocal IF microscopy. The data justify the term NET-like structures. More mature stages of NETs (bona fide NETs) implicate the spread of chromatin from the cell of origin with elongated spikes protruding into the extracellular space and bridge formation to other neutrophils [12,13,32]. These were not detected for freshly prepared AUP samples.

A set of molecular analyses, with emphasis on proteomics, was performed to corroborate the evidence of NETs as a defense strategy in UTIs. To do this, we benefitted from publications on the protein composition of NETs generated *in vitro* [14] and on mechanisms of NET formation [13,15]: NETosis requires cytoskeletal degradation and nuclear decondensation, enabled by proteolytic pathways that result in the disappearance of cellular membranes and eventual expulsion of chromatin and proteins into the extracellular milieu. First, we observed that DNase effectively homogenized AUP samples. Second, DNase treatment released proteins consistent with the existence of a DNA-protein network not protected by intact cellular membranes. Third, comparative analysis of the proteome of DNase-digested fractions from AUP samples with that of *in vitro*-NETs [14] revealed strikingly similar profiles. Such profiles were different in other fractions and in DUP samples. Forth, 0.6 to 1.2 mDa DNA-protein complexes isolated from partially DNase-digested fractions via native PAGE had a composition highly enriched in proteins that are attributed to have antibacterial effector functions in NETs (histones, MPO, NE, AZU and LTF). This data supported the physical binding of such proteins to DNA expelled from lysing neutrophils.

In further support of early-phase NETs, we identified citrullinated peptides for histones H1 and H3. The site in the histone H3 peptide (R_26_) matched one of the four known sites of citrullination sites for this protein [26]. Citrullinated peptides were also detected in other nuclear and several cytoskeleton-associated proteins raising the question as to whether this modification impacts protein function and degradation in NETs. A study on rheumatoid arthritis determined that NETs are a source of various citrullinated auto-antigens that stimulate inflammatory responses [33]. Considering the abundance of the enzyme PADI2 in AUP samples, apparently responsible for the deamidation, and several highly active proteases, we hypothesize that citrullination modulates protein binding to DNA (loss of a positive charge) and alters the susceptibility to cleavage by CTSG. A cleavage site of CTSG is the C-terminus of arginine.

Although massive proteolysis was not unique to AUP samples analyzed here and is known to occur in necrotic neutrophils, the observation of extensive protein degradation, particularly for known substrates in NETs [13,15], supported the presence of NETs in urine sediments associated with UTI. The role of the azurosome including NE in the degradation of cytoskeletal proteins, specifically F-actin, was reported [15]. While the proteolysis of cytoskeletal proteins was extensive in our analyzed AUP samples, neutrophil granule proteins were less frequently degraded to small peptides. We hypothesize that, like citrullination, proteolysis contributes to NETosis and the formation of new antimicrobial peptides, perhaps not limited to those emerging from histones, cathelicidin and α-defensin-1. Consistent with published data [28], our data support the notion that this proteolysis is driven by three neutrophil proteases, NE, PRTN3 and CTSG. Their autolytic activities may constitute another level of control over pro- and anti-inflammatory activities in the process of UTI resolution. Likewise, proteolysis of chemokines by enzymes released into NETs was previously linked to a decrease in inflammation in gout [34]. Another interesting observation was the high quantity of insoluble α-defensin-1 retained after DNase digestion of the AUP samples (UP_sol_4/5, Fig 4). It suggests that most α-defensin-1, unlike other neutrophil granule effectors, is not bound to chromatin but to lipid structures also present in early-phase NETs. Previously, α-defensins were reported to be released from necrotic and apoptotic neutrophils and to have a potent anti-inflammatory role [35]. It is of interest to elucidate the role of α-defensin-1 in inflammatory signaling pertaining to early-phase NETs associated with UTI.

To our knowledge, we characterized proteome-wide pathogen responses to potential entrapment in NETs for the first time. Analyzing 13 AUP samples, we identified seven different pathogens: E. faecalis, Serratia marcescens, P. mirabilis, C. albicans, K. pneumoniae, E. coli, and S. aureus. In some instances, a sufficient number of bacterial proteins were surveyed in UP extracts (*in vivo*) to allow comparative analysis with the proteome from lab cultures of the isolates (*in vitro*). Analyses performed for E. coli (sample #94) and S. aureus (sample #112) revealed strong decreases in proteins with functions in ribosomal translation and protein biosynthesis for *in vivo* bacteria while proteins with structural and functional roles in the cell enveloped were increased *in vivo*. These adaptations are consistent with defensive responses of the pathogens to perturbed integrity of their cell envelopes, which are under attack by a diversity of neutrophil effectors. A low level of ribosomal protein synthesis has been linked to the bacterial persistence state [36], thus offering a link of persister formation to survival in NETs. Ribosomal protein synthesis is a process consuming a lot of energy. Cellular energy sources such as ATP may instead be available to repair and regenerate damaged cell envelopes. Both E. coli and S. aureus cells produced ATP synthase *in vivo* and *in vitro*. The S. aureus proteome showed evidence of increased production of adhesins (e.g., Eap, Efb, and Spa), iron/siderophore transporters (e.g., IsdA and IsdB), proteins perturbing the function of the immune system (e.g., Spa and SCIN), and human cell toxins such as γ-hemolysin. The functional roles of these virulence factors are known [37] [38,39]. Finally, we observed two highly abundant proteins linked to cell wall autolysis (Atl and IsaA) [40,41] in the S. aureus proteome derived from the DNase-extracted AUP fraction, suggesting that their release modulates cell division and cell wall properties. The process may allow the bacteria to become more responsive to the starvation of nutrients (e.g., iron/siderophores) and improve the likelihood of immune evasion. Fluorescence-based viability assays indicated that some bacterial cells exposed to the structures we attribute to early-phase NETs survived while others were dead. We did not identify nucleases in the bacterial proteome of sample #112. Endonucleases such as EndA, produced by S. pneumoniae [16], Sda1, produced by S. pyogenes [17], and Nuc, produced by S. aureus [18], were shown to degrade chromatin and trigger bacterial escape from the entrapment in NETs. In the future, we intend to characterize the host-pathogen interactions in early-phase NETs associated with UTI in more depth. Among the most intriguing questions are proteolytic pathways that may generate novel bioactive peptides analogous to LL-37 and the strategies pathogens use to survive entrapment in NET structures.

## Methods

### Urine specimens

The human urine specimens analyzed in this study were either 1) de-identified samples collected for diagnostic purposes that otherwise would have been discarded, thus exempt from IRB review or 2) de-identified samples that were retained from an earlier IRB approved study and for which consent for future research purposes was given. Diagnostic specimens were obtained from the Pathology and Clinical Microbiology Laboratory of the Shady Grove Adventist Hospital (SGAH) in Rockville, Maryland.

### Preparation of urine specimens for fixation and microscopy experiments

The specimens were kept at 4°C for up to six hours after collection and centrifuged at 800 × g for 15 min to recover the cellular sediment. PBS was added in a 10- to 20-fold volume to the sediment and gently resuspended followed by another centrifugation step in a 1.5 ml tube for 5 min. Aliquots of this urinary pellet (UP) sample, resuspended in TBS (pH 7.5), were incubated with the *Live/Dead*-*BacLight* bacterial viability staining kit (#L7007; ThermoFisher Scientific) at SYTO9 and propidium iodide final concentrations of 5 and 55 μM, respectively or with the fluorescent dye SYTOX-Green (#L7020; ThermoFisher Scientific) for 15 min at 20°C in the dark to prepare the samples for fluorescence microscopy. Unbound dyes were removed by a 3 min centrifugation step, and pellets were gently suspended in TBS. These samples, as well as untreated UP samples suspended in PBS, were subjected to fixation adding paraformaldehyde in a 4% final concentration on a glass slide and incubating for 15 min at 20°C in the dark. Slides were rinsed with water, air-dried and stored at 4°C in the dark until used for IF staining and microscopy.

### Immunofluorescence staining experiments

Slides were viewed with an inverted Zeiss Axiovision microscope to ensure that cells and extracellular materials were immobilized. Blocking solution (2.2% BSA and 0.1% Tween-20 in PBS, 0.01% sodium azide) was added, incubating at 20°C for 75 min. The solution was replaced with a 1:25 dilution (~ 4 μg/ml) of the anti-MPO polyclonal antibody (#16128-R; Sta Cruz Biotech) in Ab diluent (PBS containing 1% BSA and 0.1% Tween-20) and incubated for 2 h at 20°C. The slide was washed five times with PBS containing 1% BSA and 0.1% Tween-20 for 3–5 min, tilting the slide occasionally to enhance exposure of fixed cells to the wash solution. Incubation with a 1:100 dilution (~ 1 μg/ml) of the goat anti-rabbit IgG antibody conjugated to the fluorescent dye CFL555 (#362271; Sta Cruz Biotech) in Ab diluent at 20°C for 75 min in the dark followed. The wash steps were repeated. Adding a drop of Vectashield Antifade Mounting Medium with DAPI (# H-1200; Vector Laboratories), the slide was incubated at 20°C for 2 h in the dark, sealed with a coverslip to stain nuclei and preserve fixed cells, and stored at 4°C in the dark until oil immersion IF microscopy was performed.

### Extraction of proteins and DNA from UP samples

Aliquots of PBS-washed UP samples, some of which underwent free-thaw cycles prior to extraction, were spun at 4000 × g for 8 min to recover the UP_sol_1 fraction. Eight UP samples were available for extractions without prior storage in the freezer at -80°C. The centrifugation conditions were also used to recover solubilized materials from subsequent extraction steps. The pellet was gently re-agitated in a 5-fold volume of PBS or TBS containing 50 mM DTT and left at 20°C for 10 min. The UP_sol_2 fraction was recovered. In some cases, this extraction step was repeated. The residual pellet was incubated with a 5- to 10-fold volume of PBS or TBS containing 2 μL bovine DNAse I (#D-4527; Sigma-Aldrich) in the presence of 5 mM MgCl_2_. One μl DNAse I corresponded to an activity of 10 Kunitz units. The suspension was incubated at 20°C for 45 min, gently agitating the tube occasionally, and in a shaker with 880 rpm at 37°C for 30 min. Upon centrifugation, the supernatant was termed the fraction UP_sol_3. The final extraction steps were performed to lyse and extract microbial cells and host cell debris. Samples containing S. aureus cells were incubated with 20 μg/ml lysostaphin in TBS (pH 8) using a shaker with 880 rpm at 37°C for 20 min. Other samples were incubated with a combination of 50 μg/ml mutanolysin and 100 μg/ml lysosome accordingly. Following addition of 5 mM EDTA and 0.4% m/V CHAPS (final concentrations), such samples were vortexed, left at 20°C for 10 min, and sonicated at the amplitude 6 in ten 30 sec on/off cycles using a Misonex 3000 sonicator ice bath. The supernatant (fraction UP_sol_4) was isolated. To the residual pellet, the SED solution containing 1% SDS, 0.3% Tween-20, 10 mM Na-EDTA, and 25 mM DTT was added. The homogenate was vortexed, left at 20°C for 10 min, and heat-denatured at 95°C for 3 min. This was followed by the aforementioned sonication step and centrifugation at 16,100 × g for 10 min to isolate the fraction UP_sol_5. The fractions were used for various molecular analysis procedures. Aliquots of unprocessed UP samples were also incubated with the SED solution at 20°C for 10 min and heat-denatured at 95°C for 3 min. Sonication and centrifugal isolation steps to recover the supernatants of these UP lysates were done as described for the fractions UP_sol_4 and UP_sol_5.

### SDS-PAGE, proteomic, and peptidomic sample preparations

Lysates and fractions derived from UP samples were subjected to SDS-PAGE in 4–12% acrylamide gradient gels and stained with the protein dye Coomassie Brilliant Blue G-250 (CBB). The gel staining intensity was used to estimate the overall protein amount in a given sample for the processing steps of filter-aided sample preparation (FASP), comparing the staining intensity with 2 μg BSA. For FASP analysis, where the intent is to separate and concentrate a fraction of denatured proteins larger than approximately 5 kDa from low M_r_ molecules including peptides, a membrane concentrator device (Sartorius, Germany) with a 10 kDa M_r_ cut-off was used. Using trypsin as the enzyme to digest a UP lysate or UP_sol_ fraction, we applied a published experimental approach [42,43]. For in-gel digestion of proteins, SDS-PAGE gel bands were excised and digested with trypsin as also previously reported [44]. Digestion products derived from FASP were desalted using the spinnable StageTip protocol [45]. Eluates containing enriched peptide mixtures were applied to LC-MS/MS. For the analysis of peptides contained in the small molecule fraction of UP samples, the combined fractions of UP_sol_1-UP_sol_3 were applied to the 10 kDa concentrator device recovering the filtrate by spinning at 16,100 × g. The filtrate was also subjected to the StageTip protocol to enrich for peptides [45].

### LC-MS/MS

Peptide mixtures were analyzed using an LC-MS/MS platform consisting of the Ultimate 3000-nano LC coupled via a FLEX nano-electrospray ion source to a Q-Exactive mass spectrometer (Thermo Scientific, USA). We have described the LC-MS/MS experimental and data acquisition methods in detail previously [19,43]. Parallel to an LC solvent gradient elution time of 110 min, peptide ions were analyzed in a MS^1^ data-dependent mode to select ions for the MS^2^ scans using the XCalibur software *v2*.*2* (Thermo Scientific). Survey scans were acquired at a resolution of 70,000 (m/Δm) over a mass range of m/z 250–1,800. In each cycle, the ten most intense ions were subjected to fragmentation applying normalized collision energy of 27%. The MS^2^ scans were performed at a resolution of 17,500. Ions that were unassigned or had a charge of +1 were rejected from further analysis. Two or three replicate LC-MS/MS experiments were run, and raw MS files were combined for database searches. The database was either a version of the human UniProtKB database (release 2013_6) reduced in protein redundancy (27,151 entries) or the former complemented by protein sequence entries of genomes representing the microbial species identified in the investigated UP samples. The microbial database identifiers were reported previously [19]. This set of methods was applied to analyze gel extract, proteome, and peptidome samples. The software tool Proteome Discoverer *v1*.*4* (Thermo Scientific) was used to interpret data qualitatively and confirm mass spectral qualities of individual peptides, using search parameters for mass tolerance, proteolytic cleavage sites for trypsin, and amino acid modifications as described [43]. To evaluate proteolytic cleavages by the enzymes NE, PRTN3, and CTSG, the parameters were modified selecting ‘elastase’ (C-terminal to A, V, I, L, M, S, C, T as reported in [28]) in addition to ‘trypsin’. For peptidome analyses, cleavage at any position in the amino acid sequence was allowed. The false discovery rate (FDR) was set at 1% for protein IDs.

### Quantitative proteome analysis and bioinformatics methods

Raw MS raw files were imported into the MaxQuant software suite (*v1*.*4*.*2*) [46] accepting the default settings for quantification via MS^1^ peak integration and normalization of proteomic data comparing multiple samples (S2 Data). We used the intensity-based absolute quantification (iBAQ) function enabled in MaxQuant to estimate protein abundances in all of the analyzed samples [47]. Processing data in Excel, the iBAQ quantity of a protein in a given sample was divided by the sum of all iBAQ quantities. This method of estimating relative iBAQ values was applied to human and microbial proteome datasets. One analysis pertained to the comparison of the human proteome present in UP_sol_ fractions and *in vitro*-NETs). Another analysis pertained to the *S*. *aureus* proteome in UP_sol_ fractions versus lysates from *in vitro* cultures. Information on protein functional role categories, their subcellular localizations and post-translational modifications reflected information provided for protein entries in the UniProt database [48].

### Native PAGE and western blotting

Native-PAGE gels (Life Technologies, Carlsbad, CA) with a 3% acrylamide concentration were used to examine the co-migration of proteins and DNA molecules. We used the experimental procedures recommended by the product manufacturer. Gels were stained with CBB and ethidium bromide to visualize proteins and DNA, respectively. SDS-PAGE in 4–12% acrylamide gradient gels was performed loading 5 to 20 μg total protein from several UP_sol_ fractions. The gels were blotted onto PVDF membranes and incubated with antibodies, washed, and developed using chemiluminescence, as previously described [49]. The following polyclonal antibodies raised against peptide segments (Santa Cruz Biotechnology, Dallas, Texas) were used: anti-MPO heavy chain C-16 (sc-16128-R), anti-LTF (H-65, sc-25622), anti-ELANE (H-57, sc-25621), anti-histone H4 (H-97, sc-10810), and a horseradish peroxidase conjugate of goat anti-rabbit IgG-HRP (sc-2004). Primary antibodies and secondary antibody conjugates were diluted 1:1,000 and 1:10,000, respectively, for 90 min incubation steps. The peroxidase activity of MPO was measured with the substrate 3,3’,5,5’-Tetramethylbenzidine (KPL, Gaithersburg, MD; #54-11-50) in a colorimetric endpoint assay over 4 min measuring the absorption of the product at 650 nm.

### Bacterial strains and cell cultures

Two isolates, a S. aureus strain from UP sample #112 and an E. coli strain from UP sample #134, were recovered from LB agar plates grown for 12–24 h aerobically at 37°C. After confirming species identities by Gram staining and LC-MS/MS analysis and an overnight pre-culture, 20–25 ml fresh LB media were inoculated at an OD_600_ of ca. 0.05 for suspension cultures in a shaker at 880 rpm set at 37°C. The cultures were stopped when the OD_600_ values reached 0.35–0.5 (mid-exponential phase; ~4–6 h) and 0.9–1.2 (stationary phase; ~14 h). Bacterial cells were lyzed by addition of the SED solution, letting the suspension sit for 10 min vortexing a few times, applying heat at 95°C for 3 min, and sonication as described for the UP_sol_5 extraction step. For S. aureus cell lysis, the initial incubation step was cell wall digestion with lysostaphin for 20 min followed by the cell lysis protocol. The S. aureus strain HIP5827 used for experiments to detect the cell surface protein Spa was described earlier [50].

## Supporting Information

S1 Data Protein and DNA extraction profiles from urinary pellet (UP) samples associated with UTIs. (PDF)

S2 Data Overview of experiments applied to urinary pellet (UP) samples to detect and characterize neutrophils extracellular traps (NETs). (XLSX)

S3 Data Neutrophil-enriched proteomic datasets for fractions derived from extractions of 13 UP samples to assess NET formation in UTI cases. (XLSX)

S4 Data Citrullinated peptides of proteins isolated from AUP samples. (PDF)

S5 Data Protein cleavage sites consistent with the P1 site specificities of neutrophil elastase and proteinase 3, identified for proteins abundant in AUP samples. (XLSX)

S6 Data Proteomic and peptidomic datasets for ten UP samples. (XLSX)

S7 Data The Staphylococcus aureus and Escherichia coli proteomes from AUP samples #112 and #94, respectively, in comparison with the *in vitro* proteomes of these two isolates. (XLSX)
