# Supplementary material for: Characterization of Early-Phase Neutrophil Extracellular Traps in Urinary Tract Infections
Source: PLoS Pathog. 2017 Jan 27;13(1):e1006151. doi: 10.1371/journal.ppat.1006151 (PMC5298345; doi:10.1371/journal.ppat.1006151)
Supplement: S1 Data — (PDF) [file ppat.1006151.s001.pdf]

Supplementary Data S1: Protein and DNA extraction profiles from urinary pellet (UP) samples associated with UTIs

Page 2 (Figure 1). SDS-PAGE analysis of urinary pellet (UP) lysates including aggregate-forming UP (AUP) samples and easily dispersed UP (DUP) samples

The lysates were generated by suspension of an aliquot of a urinary pellet in a 1% SDS solution containing 10 mM Na-EDTA and 20 mM DTT. Samples were boiled at 95ºC for 5 min, cooled to room temperature and spun at 16,100 x g for 5 min to remove any unsolubilized matter. The SDS-PAGE gels (4-25% NuPAGE gels) were stained with Coomassie Brilliant Blue G250 to visualize the proteins. The sample identifier (sample ID) is used throughout the Supplementary Data S1 and the main manuscript.

Pages 3-22 (Figures 2a to 2t). DNA and protein extraction profiles from UP samples with evidence of aggregation and protein solubilization during a DNAse I incubation step - suggesting NET formation - and control UP samples that did not form aggregates

Page 23 (Figure 3). Western blot confirming the reactivity of S. aureus protein A (Spa) with anti-rabbit IgG HRP conjugate

General descriptions for the data in Figures 2A to 2R:
This dataset pertains to the extraction of urinary pellet (UP) samples that were visually inspected for physical appearance and aggregation behavior. We examined aggregate-forming UP (AUP) samples. Such aggregates rich in neutrophils formed upon re-suspension of the urinary pellets in PBS or in PBS supplemented with dithiothreitol (DTT). We also examined a few UP samples that were not aggregating in the process of resuspension and extraction with PBS-DTT. The pellets remained dispersed after re-suspension (DUP samples). The extractions for one UP sample was repeated (#134 in this dataset). Freeze-thaw cycles caused contributed to the aggregation of cellular and extracellular matter in the AUP samples.

The first AUP sample (#33) on Page 3 contains extensive information of the fractionation methods and observations related to many AUP samples. The DUP samples (as controls) were from clean catch urine pellets with relatively low neutrophil counts (#9; #29; #36; #55; #88) and one sample derived from a catheter biofilm (#64): Pages 4 to 9. I many but not all cases, a DNA agarose gel was run together with an SDS-PAGE gel to visualize the released DNA in the isolated fractions.

Please note that the sample identifiers (IDs) are the same on Page 1 (UP lysates) and on Pages 3-22 (fractions of the lysates).

Figure 1

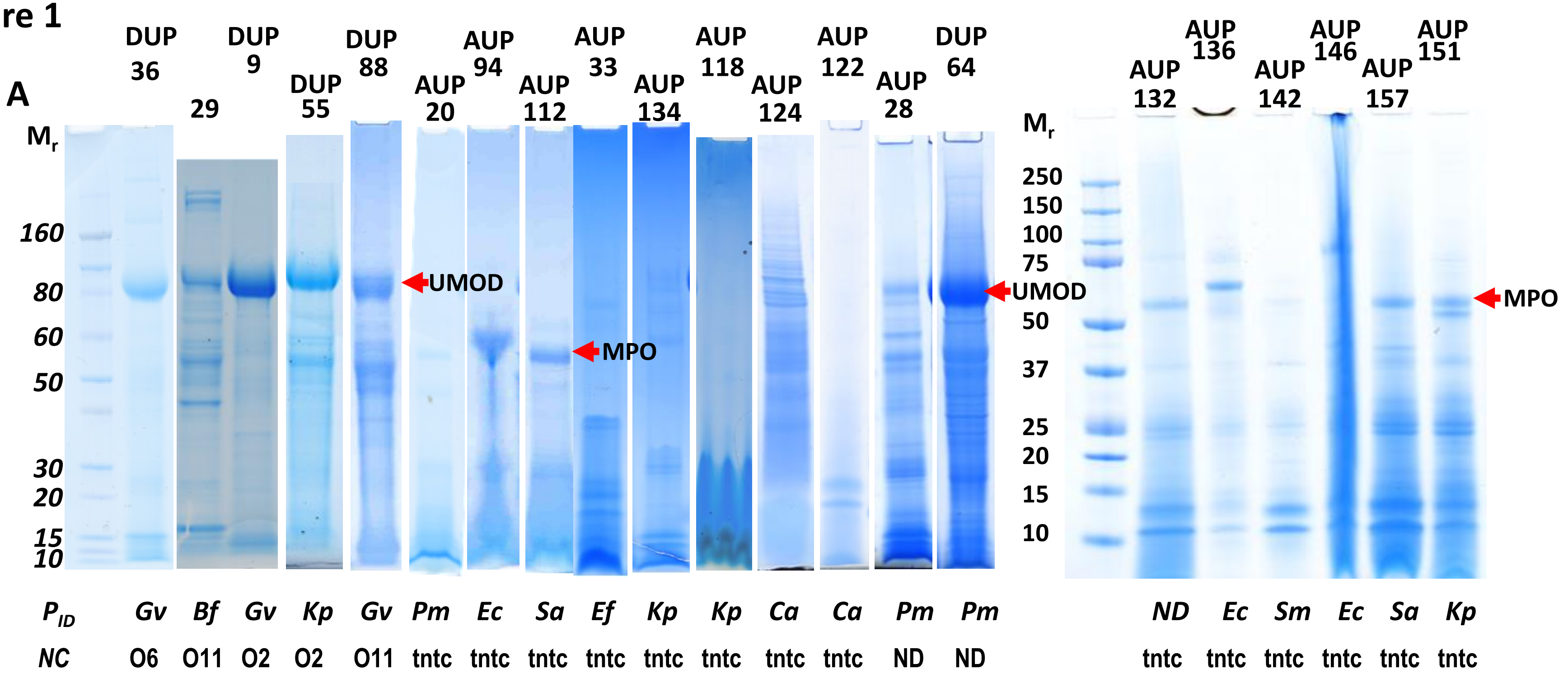

P_{ID}, species identfied by LC-MS/MS and microbial culture
Gv, Gardnerella vaginalis
Bf, Bacteroides fragilis
Kp, Klebsiella pneumoniae
Pm, Proteus mirabilis
Ec, Escherichia coli
Sa, Staphylococcus aureus
Ef, Enteroccocus faecalis
Ca, Candida albicans
Sm, Serratia marcescens
ND, not determined
(please not that these were the dominant microbial organisms; a few samples had evidence of up to three pathogens / commensals of the urogenital tract)

NC: leukocyte (neutrophil) counts assessed by standard urine sediment microscopy with a hemocytometer per high-power field
O2: more than 2 per HPF
O6: more than 2 per HPF
O11: more than 2 per HPF
tntc: leukocytes too numerous to count

Figure 2a

Date: 04-15-15

NuPAGE gel, 4-12% acrylamide, Coomassie Brilliant Blue G250 stained

AUP sample 33

1: $UP_{sol}1$, ca. 11 ul extract
2: $UP_{sol}2a$, ca. 11 ul extract
3: $UP_{sol}2b$, ca. 11 ul extract
4: $UP_{sol}3$, ca. 11 ul extract
5: $UP_{sol}4$, ca. 11 ul extract
6: $UP_{sol}5$, ca. 11 ul extract
7: M_r standard, 5 ul solution

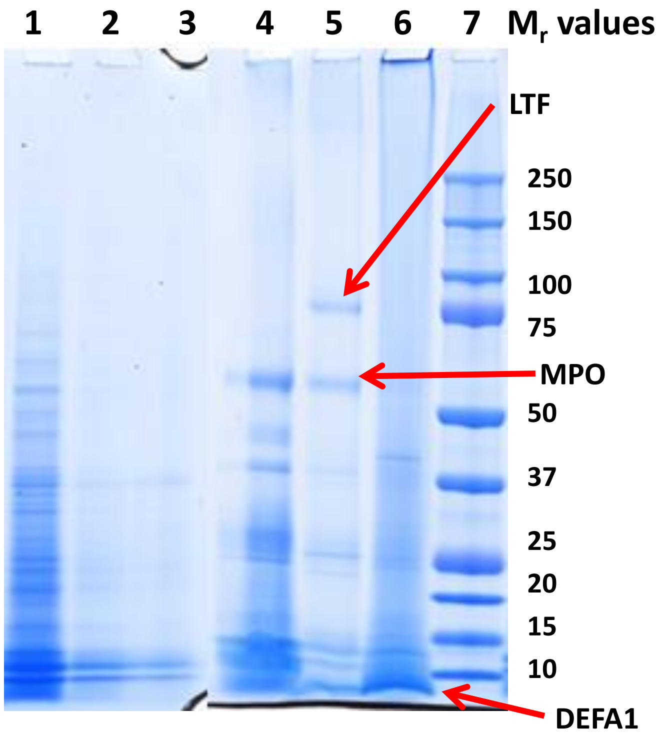

LTF: lactotransferrin
MPO: myeloperoxidase
DEFA1: neutrophil α-defensin 1
(abbreviations also used in other images)

● Sample 33 was viscous and clumpy. *In vitro* culture and proteomic analysis revealed *Enterococcus faecalis* as the infectious agent. The extractions related to this experiment were performed after a freeze/thaw cycle. The sample 33 remained clumpy and viscous and was not homogenized upon incubation with PBS and PBS + 50 mM DTT ($UP_{sol}1$ and $UP_{sol}2$ fractions, respectively). The pellet derived from the $UP_{sol}2$ extraction step was incubated with deoxyribonuclease I (DNAse I)for 60 min, resulting in the fraction $UP_{sol}3$.
● Incubation steps with 50 µg/ml mutanolysin and 100 µg/ml lysosome in PBS or TBS followed and 10 mM EDTA and 0.4% CHAPS were added. Samples were vortexed, left at 20°C for 10 min, and sonicated at the amplitude 6 in ten 30 sec on/off cycles using a Misonex 3000 sonicator while cooling on ice. Following centrifugation for 6 min at 8,000 x g, a supernatant termed $UP_{sol}4$ was isolated. The insoluble pellet was incubated with the SED solution (1% SDS, 0.3% Tween-20, 10 mM EDTA, 25 mM DTT) for 30 min followed by sonication at the amplitude 6 for 5 min (Misonex 3000 sonicator). The homogenate was vortexed, left at 20°C for 10 min, and heat-denatured at 95°C for 3 min. This was followed by repeated sonication step and centrifugation at 16,100 × g for 10 min to isolate the fraction $UP_{sol}5$.

Summary:
Step 1: extraction with PBS ($UP_{sol}1$);
Step 2: two successive extraction steps with PBS + 50 mM DTT ($UP_{sol}2a$ and $UP_{sol}2b$);
Step 3: incubation/extraction with PBS and DNAse I ($UP_{sol}3$);
Step 4: extraction/incubation with lysozyme and mutanolysin in the presence of the partially membrane-solubilizing reagents EDTA and CHAPS ($UP_{sol}4$);
Step 5: incubation/heat extraction with denaturing solution ($UP_{sol}5$);

Figure 2b

Date: 04-22-16
NuPAGE gel, 4-12% acrylamide, Coomassie Brilliant Blue G250 stained
DUP sample 9

1: $UP_{sol}1$, ca. 10 ul extract
2: $UP_{sol}2$, ca. 10 ul extract
3: $UP_{sol}3$, ca. 10 ul extract
4: $UP_{sol}5$, ca. 10 ul extract
5: M_r standard, 5 ul solution

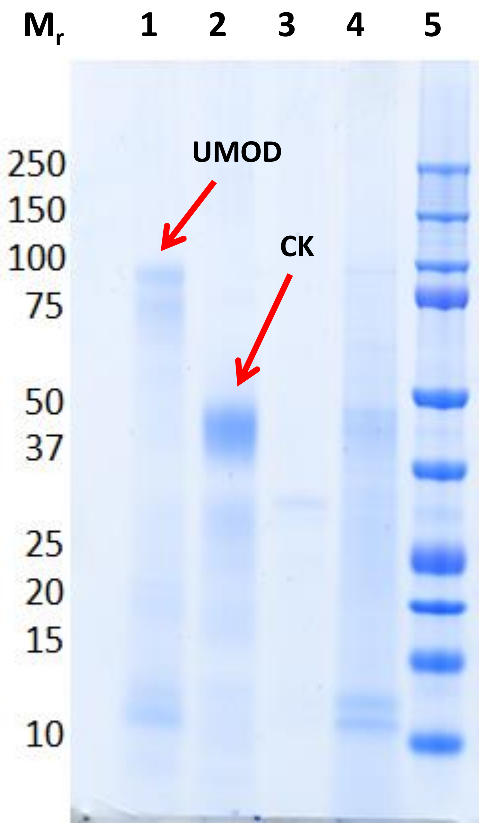

● Sample 9 was neither aggregated nor clumpy after resuspension in PBS. Proteomic analysis revealed presence of *Gardnerella vaginalis* with no evidence that the bacterium caused a urinary tract infection although some neutrophils were microscopically identified. The extraction experiments shown here were performed after a freeze/thaw cycle. The pellet of sample 9 was homogenized upon incubation with PBS and PBS + 50 mM DTT ($UP_{sol}1$ and $UP_{sol}2$ fractions, respectively). The pellet derived from the $UP_{sol}2$ extraction step was small and was incubated with DNAse I for 60 min, resulting in the fraction $UP_{sol}3$.
● In this experiment, the following extraction steps were identical to those described in Figure 1A, with the exception that the extraction step resulting in $UP_{sol}4$ was not performed.

Summary:
Step 1: extraction with PBS ($UP_{sol}1$);
Step 2: extraction with PBS + 50 mM DTT ($UP_{sol}2$);
Step 3: incubation/extraction with PBS and DNAse I ($UP_{sol}3$);
Step 4: incubation/heat extraction with denaturing solution ($UP_{sol}5$);

Figure 2c

Date: 12-17-13
NuPAGE gel, 4-12% acrylamide, Coomassie Brilliant Blue G250 stained
DUP sample 29

1: M_r standard, 5 ul solution
2: $UP_{sol}1$, ca. 11 ul extract
3: $UP_{sol}2a$, ca. 11 ul extract
4: $UP_{sol}2b$, ca. 11 ul extract
5: $UP_{sol}3$, ca. 11 ul extract
6: DNAse I, 2 ug
7: $UP_{sol}5$, ca. 6 ul extract

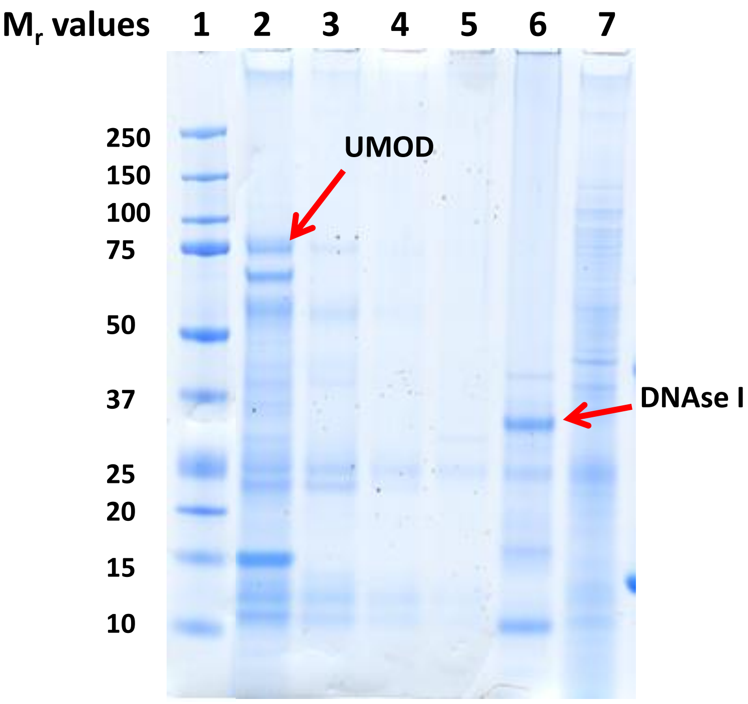

UMOD: uromodulin
DNAse I: deoxyribonuclease I
(abbreviations also used in other images)

● Sample 29 was not aggregated or clumpy after resuspension in PBS. Proteomic analysis revealed the presence of *Bacteroides fragilis* with no evidence that the bacterium caused a urinary tract infection although some neutrophils were microscopically identified. The extraction experiments shown here were performed after a freeze/thaw cycle. The pellet of sample 29 was homogenized upon incubation with PBS and then PBS + 50 mM DTT ($UP_{sol}1$ and $UP_{sol}2$ fractions, respectively). The pellet derived from $UP_{sol}2$ extraction was small. Incubation with DNAse I for 60 min, resulting in fraction $UP_{sol}3$.
● In this experiment, the following extraction steps were equivalent to those described in Figure 1A, with the exception that the extraction step resulting in $UP_{sol}4$ was not performed.

Step 1: extraction with PBS ($UP_{sol}1$);
Step 2: two successive extraction steps with PBS + 50 mM DTT ($UP_{sol}2a$ and $UP_{sol}2b$);
Step 3: incubation/extraction with PBS and DNAse I ($UP_{sol}3$);
Step 4: incubation/heat extraction with denaturing solution ($UP_{sol}5$);

Figure 2d

Date: 05-24-16
NuPAGE gel, 4-12% acrylamide, Coomassie Brilliant Blue G250 stained
<u>DUP sample 36</u>

1: M_r standard, 5 ul solution
2: $UP_{sol}3$, ca. 10 ul extract
3: $UP_{sol}2$, ca. 10 ul extract
4: $UP_{sol}1$, ca. 10 ul extract
5: $UP_{sol}5$, ca. 10 ul extract
6: Bovine serum albumin, ca. 2 ug

● Sample 20 was neither aggregated nor clumpy after resuspension in PBS. Proteomic analysis revealed presence of *Gardnerella vaginalis* with no evidence that the bacterium caused a urinary tract infection although some neutrophils were microscopically identified. The extraction experiments shown here were performed after a freeze/thaw cycle. The pellet of sample 36 was homogenized upon incubation with PBS and PBS + 50 mM DTT ($UP_{sol}1$ and $UP_{sol}2$ fractions, respectively). The pellet derived from the $UP_{sol}2$ extraction step was small and was incubated with DNAse I for 60 min, resulting in the fraction $UP_{sol}3$.
● In this experiment, the following extraction steps were identical to those described in Figure 1A, with the exception that the extraction step resulting in $UP_{sol}4$ was not performed.

Summary:
Step 1: extraction with PBS ($UP_{sol}1$);
Step 2: extraction with PBS + 50 mM DTT ($UP_{sol}2$);
Step 3: incubation/extraction with PBS and DNAse I ($UP_{sol}3$);
Step 4: incubation/heat extraction with denaturing solution ($UP_{sol}5$);

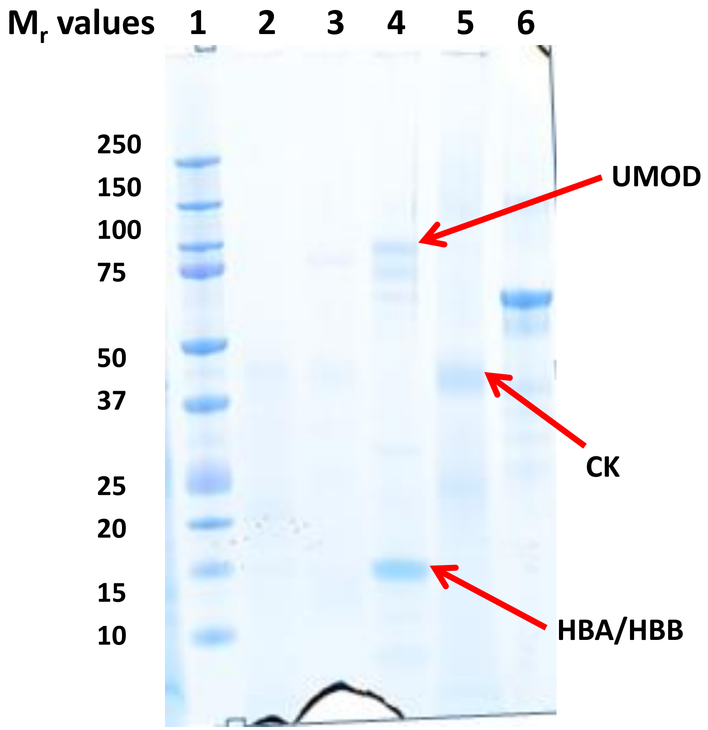

HBA/HBB: hemoglobin α and β
CK: cytokeratins
(abbreviations also used in other images)

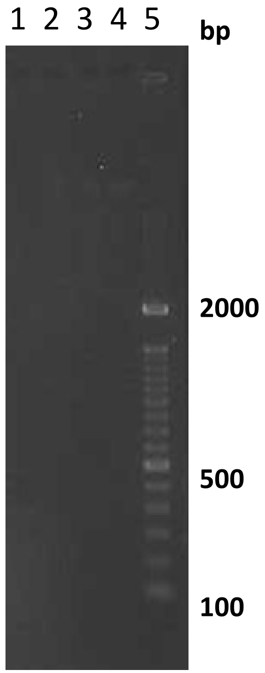

Date: 06-22-16
0.5% agarose gel, ethidium bromide stained
Sample 36
1: $UP_{sol}1$, ca. 5 ul extract
2: $UP_{sol}2$, ca. 5 ul extract
3: $UP_{sol}3$, ca. 5 ul extract
4: $UP_{sol}5$, ca. 5 ul extract
5: DNA standard, 1 ul solution

Figure 2e

Date: 12-19-13
NuPAGE gel, 4-12% acrylamide, Coomassie Brilliant Blue G250 stained
<u>DUP sample 55</u>

1: M_r standard, 5 ul solution
2: UP_{sol}1a, ca. 11 ul extract
3: UP_{sol}1b, ca. 11 ul extract
4: UP_{sol}2a, ca. 11 ul extract
5: UP_{sol}2b, ca. 11 ul extract
6: UP_{sol}3, ca. 11 ul extract
7: DNAse I, 2 ug
8: UP_{sol}5, ca. 6 ul extract

● Sample 55 was neither aggregated nor clumpy after resuspension in PBS. Proteomic analysis revealed presence of the uropathogen *Klebsiella pneumoniae* with no evidence that the bacterium elicited an immune response and urinary tract infection. Few neutrophils were microscopically identified. The extraction experiments shown here were performed after a freeze/thaw cycle. The pellet of sample 55 was homogenized upon incubation with PBS and PBS + 50 mM DTT (UP_{sol}1 and UP_{sol}2 fractions, respectively). The pellet derived from the UP_{sol}2 extraction step was small and was incubated with DNAse I for 60 min, resulting in the fraction UP_{sol}3.
● In this experiment, the following extraction steps were identical to those described in Figure 1A, with the exception that the extraction step resulting in UP_{sol}4 was not performed.

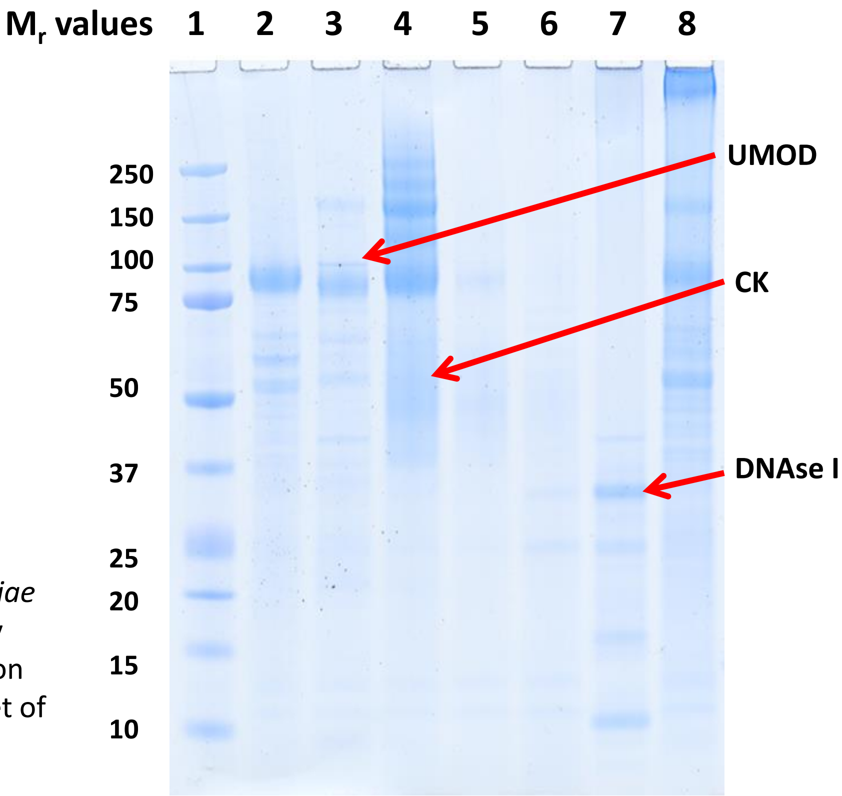

Summary:
Step 1: two extraction steps with PBS (UP_{sol}1a and UP_{sol}1b);
Step 2: two extraction steps with PBS + 50 mM DTT (UP_{sol}2a and UP_{sol}2b);
Step 3: incubation/extraction with PBS and DNAse I (UP_{sol}3);
Step 4: incubation/heat extraction with denaturing solution (UP_{sol}5);

Figure 2f

Date: 05-24-16
NuPAGE gel, 4-12% acrylamide, Coomassie Brilliant Blue G250 stained
DUP sample 64

1: M_r standard, 5 ul solution
2: UP_{sol}1a, ca. 11 ul extract
3: UP_{sol}2a, ca. 11 ul extract
4: UP_{sol}2b, ca. 11 ul extract
5: UP_{sol}3, ca. 11 ul extract
6: DNAse I, 2 ug
7: UP_{sol}5, ca. 6 ul extract

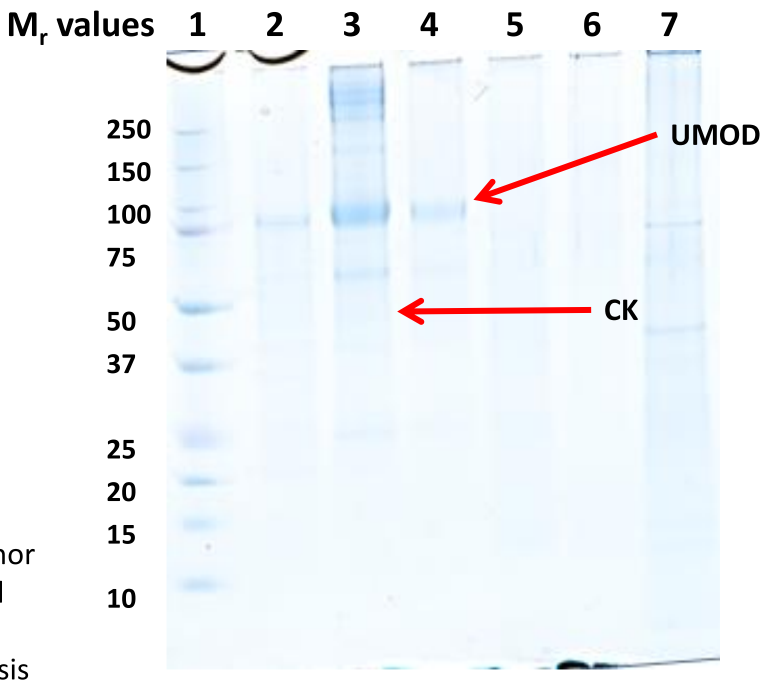

● Sample 64 was derived from a urinary sediment that was neither aggregated nor clumpy after resuspension in PBS or PBS-DTT. The human subject had a urethral catheter-associated infection (CAUTI), and this sample was obtained after the catheter-associated biofilm dispersed into the urine sediment. Proteomic analysis revealed *Proteus mirabilis* as the infectious agent. The extraction experiments shown here were performed after a first freeze/thaw cycle. The pellet of sample 64 was homogenized upon incubation with PBS and PBS + 50 mM DTT (UP_{sol}1 and UP_{sol}2 fractions, respectively). The pellet derived from the UP_{sol}2 extraction was relatively large. Incubation with DNAse I for 60 min did not change the pellet volume and resulted in fraction UP_{sol}3.
● In this experiment, the following extraction steps were identical to those described in Figure 1A, with the exception that the extraction step resulting in UP_{sol}4 was not performed.

Summary:
Step 1: extraction step with PBS (UP_{sol}1a);
Step 2: two extraction steps with PBS + 50 mM DTT (UP_{sol}2a and UP_{sol}2b);
Step 3: incubation/extraction with PBS and DNAse I (UP_{sol}3);
Step 4: incubation/heat extraction with denaturing solution (UP_{sol}5);

Figure 2g

Date: 04-22-15
NuPAGE gel, 4-12% acrylamide, Coomassie Brilliant Blue G250 stained
<u>DUP sample 88</u>

1: M_r standard, 5 ul solution
2: $UP_{sol}1$, ca. 10 ul extract
3: $UP_{sol}2$, ca. 10 ul extract
4: $UP_{sol}3$, ca. 10 ul extract
5: $UP_{sol}5$, ca. 10 ul extract

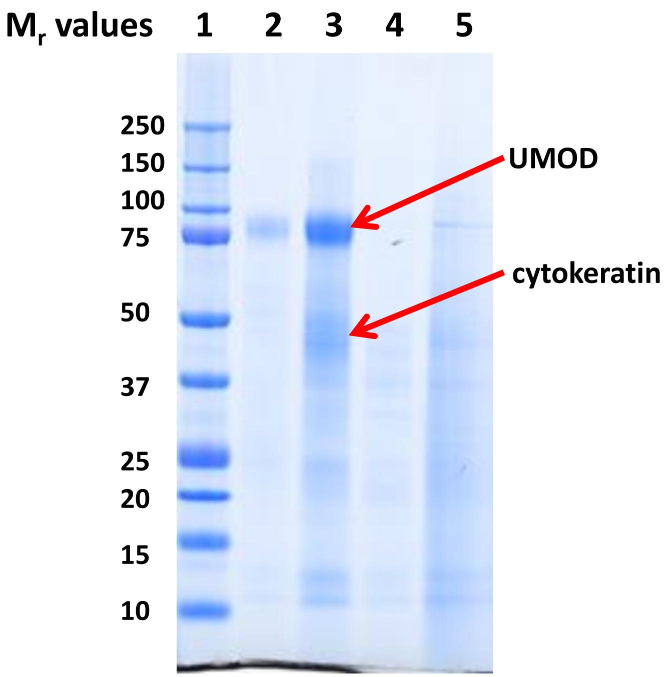

● Sample 88 was neither aggregated nor clumpy after resuspension in PBS. Proteomic analysis revealed presence of the vaginal commensals *Gardnerella vaginalis and Prevotella melanogenica* with no evidence that the bacterium elicited an immune response and urinary tract infection. Few neutrophils were microscopically identified. The extraction experiments shown here were performed after a freeze/thaw cycle. The pellet of sample 88 was homogenized upon incubation with PBS and PBS + 50 mM DTT ($UP_{sol}1$ and $UP_{sol}2$ fractions, respectively). The pellet derived from the $UP_{sol}2$ extraction step was small and was incubated with DNAse I for 60 min, resulting in the fraction $UP_{sol}3$.
● In this experiment, the following extraction steps were identical to those described in Figure 1A, with the exception that the extraction step resulting in $UP_{sol}4$ was not performed.

Summary:
Step 1: extraction with PBS ($UP_{sol}1$);
Step 2: extraction with PBS + 50 mM DTT ($UP_{sol}2$);
Step 3: incubation/extraction with PBS and DNAse I ($UP_{sol}3$);
Step 4: incubation/heat extraction with denaturing solution ($UP_{sol}5$);

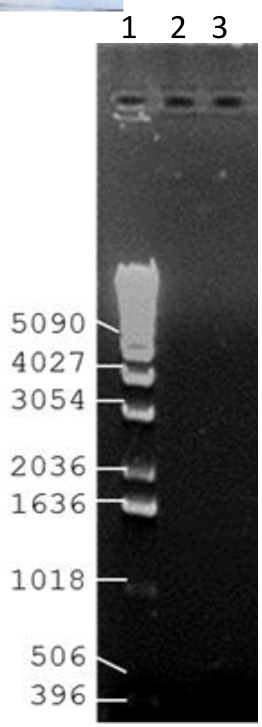

Date: 04-28-15
0.5% agarose gel, ethidium bromide stained
Sample 88

1: 5: DNA standard, 1 ul solution
2: $UP_{sol}1$, ca. 3 ul extract
3: $UP_{sol}3$, ca. 3 ul extract

Figure 2h

Date: 02-12-15
NuPAGE gel, 4-12% acrylamide, Coomassie Brilliant Blue G250 stained
<u>AUP sample 112</u>
(sample subjected to a freeze/thaw cycle prior to extraction of protein; sample for DNA analysis subjected to two freeze-thaw cycles)

1: $UP_{sol}1$, ca. 10 ul extract
2: $UP_{sol}2a$, ca. 10 ul extract
3: $UP_{sol}2b$, ca. 10 ul extract
4: $UP_{sol}3$, ca. 10 ul extract
5: $UP_{sol}5$, ca. 10 ul extract
6: $UP_{sol}5$, ca. 10 ul extract
7: M_r standard, 5 ul solution

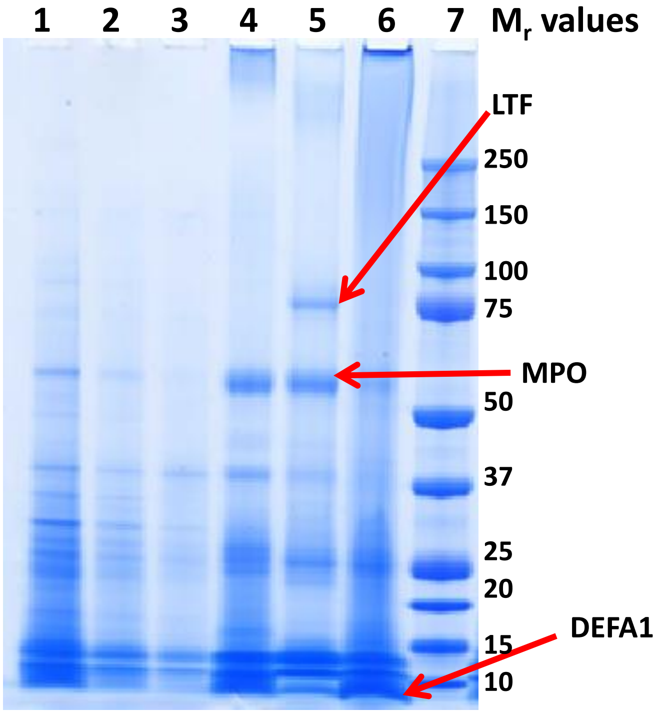

● Sample 112 was viscous and clumpy. *In vitro* culture and proteomic analysis revealed *Staphylococcus aureus* as the infectious agent. The extractions related to this experiment were performed after a freeze/thaw cycle. The pellet of sample 112 remained clumpy and viscous and was not homogenized upon incubation with PBS and PBS + 50 mM DTT ($UP_{sol}1$ and $UP_{sol}2$ fractions, respectively).
● In this experiment, the following extraction steps were equivalent to those described in Figure 1A.

Summary:
Step 1: extraction with PBS ($UP_{sol}1$);
Step 2: two successive extraction steps with PBS + 50 mM DTT ($UP_{sol}2a$ and $UP_{sol}2b$);
Step 3: incubation/extraction with PBS and DNAse I ($UP_{sol}3$);
Step 4: extraction/incubation with lysozyme and mutanolysin in the presence of the partially membrane-solubilizing reagents EDTA and CHAPS ($UP_{sol}4$);
Step 5: incubation/heat extraction with denaturing solution ($UP_{sol}5$);

Date: 06-23-16
0.5% agarose gel, ethidium bromide stained
Sample 112

1: DNA standard, 1 ul solution
2: $UP_{sol}1$, ca. 3 ul extract
3: $UP_{sol}2a$, ca. 3 ul extract
4: $UP_{sol}2b$, ca. 3 ul extract
5: $UP_{sol}3$, ca. 3 ul extract, incubation stopped with EDTA after 10 min
6: $UP_{sol}3$, ca. 3 ul extract, incubation stopped with EDTA after 60 min

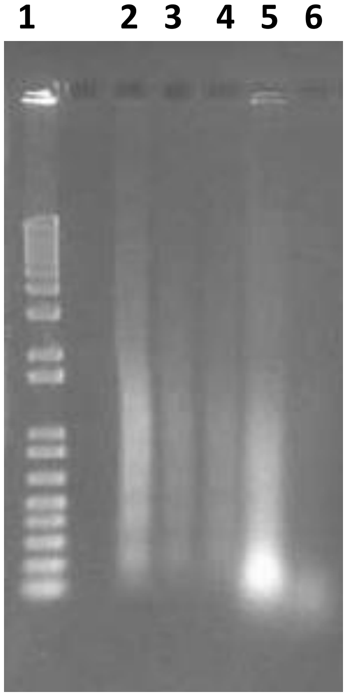

Figure 2i

Date: 04-30-15
NuPAGE gel, 4-12% acrylamide, Coomassie Brilliant Blue G250 stained
AUP sample 118

1: $UP_{sol}1$, ca. 10 ul extract
2: $UP_{sol}2a$, ca. 10 ul extract
3: $UP_{sol}2b$, ca. 10 ul extract
4: $UP_{sol}3$, ca. 10 ul extract
5: $UP_{sol}5$, ca. 10 ul extract
6: M_r standard, 5 ul solution

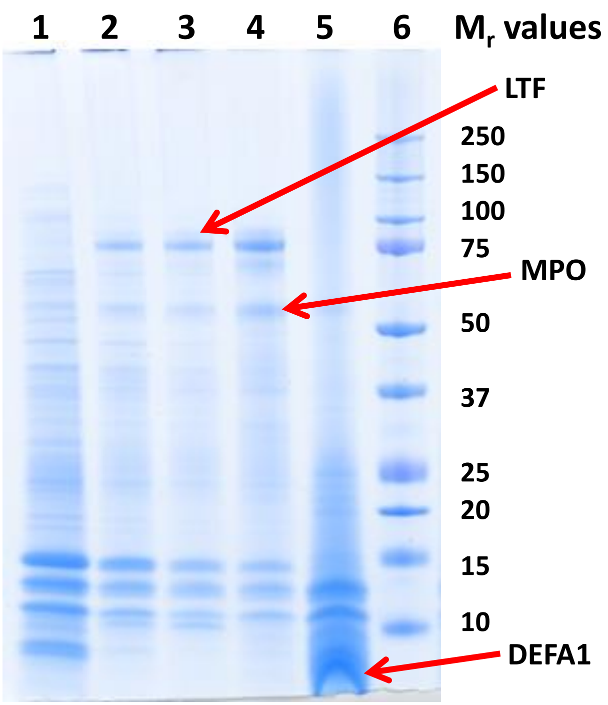

● Sample 118 was viscous and clumpy. *In vitro* culture and proteomic analysis revealed *Klebsiella pneumoniae* and Escherichia coli as the infectious agents. The extractions related to this experiment were performed after a freeze/thaw cycle. The pellet of sample 118 became less clumpy and viscous but was not completely homogenized upon incubation with PBS and PBS + 50 mM DTT ($UP_{sol}1$ and $UP_{sol}2$ fractions, respectively). Homogenization was achieved with the DNAse I incubation step.

● In this experiment, the following extraction steps were equivalent to those described in Figure 1A, with the exception that the extraction step resulting in $UP_{sol}4$ was not performed.

Summary:
Step 1: extraction with PBS ($UP_{sol}1$);
Step 2: two extraction steps with PBS + 50 mM DTT ($UP_{sol}2a$ and $UP_{sol}2b$);
Step 3: incubation/extraction with PBS and DNAse I ($UP_{sol}3$);
Step 4: incubation/heat extraction with denaturing solution ($UP_{sol}5$);

Figure 2j

Date: 04-15-15
NuPAGE gel, 4-12% acrylamide, Coomassie Brilliant Blue G250 stained
<u>AUP sample 94</u>

1: $UP_{sol}1$, ca. 10 ul extract
2: $UP_{sol}2$, ca. 10 ul extract
3: $UP_{sol}3$, ca. 10 ul extract
4: $UP_{sol}5$, ca. 10 ul extract
5: M_r standard, 10 ul solution

● Sample 94 was viscous and clumpy. *In vitro* culture and proteomic analysis revealed *Escherichia coli* as the infectious agent. The extractions related to this experiment were performed after a freeze/thaw cycle. The pellet of sample 94 remained clumpy and viscous and was not homogenized upon incubation with PBS and PBS + 50 mM DTT ($UP_{sol}1$ and $UP_{sol}2$ fractions, respectively).
● In this experiment, the following extraction steps were equivalent to those described in Figure 1A, with the exception that the extraction step resulting in $UP_{sol}4$ was not performed.

Summary:
Step 1: extraction with PBS ($UP_{sol}1$)
Step 2: extraction with PBS + 50 mM DTT ($UP_{sol}2a$);
Step 3: re-extraction with PBS + 50 mM DTT ($UP_{sol}2b$); gel lane not shown
Step 4: incubation/extraction with PBS and DNAse I ($UP_{sol}3$);
Step 5: incubation/heat extraction with denaturing solution ($UP_{sol}5$);

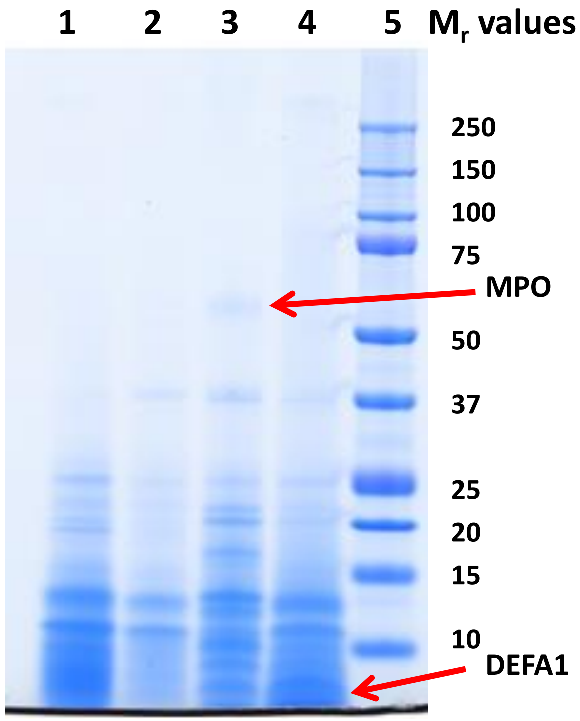

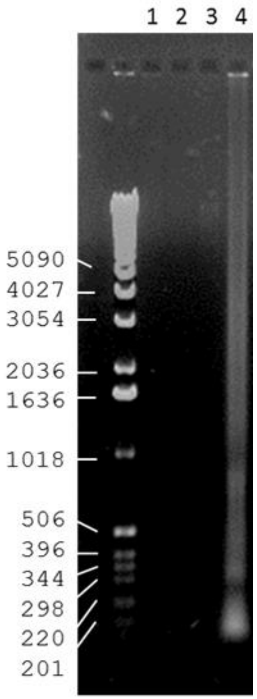

Date: 04-27-15
0.5% agarose gel, ethidium bromide stained
Sample 94

left: 5: DNA standard, 1 ul solution
1: $UP_{sol}1$, ca. 1 ul extract
2: $UP_{sol}2a$, ca. 1 ul extract
3: $UP_{sol}2b$, ca. 1 ul extract
4: $UP_{sol}3$, ca. 1 ul extract

Figure 2k

Date: 02-13-15
NuPAGE gel, 4-12% acrylamide, Coomassie Brilliant Blue G250 stained
<u>AUP sample 134</u>

1: M_r standard, 5 ul solution
2: $UP_{sol}1$, ca. 11 ul extract
3: $UP_{sol}2a$, ca. 11 ul extract
4: $UP_{sol}2b$, ca. 11 ul extract
5: $UP_{sol}3$, ca. 11 ul extract
6: $UP_{sol}4$, ca. 11 ul extract
7: $UP_{sol}5$, ca. 11 ul extract

● Sample 134 was viscous and clumpy. *In vitro* culture and proteomic analysis revealed *Klebsiella pneumoniae* as the infectious agent. The extractions related to this experiment were performed after a freeze/thaw cycle. The pellet of sample 134 remained clumpy and viscous and was not homogenized upon incubation with PBS and PBS + 50 mM DTT ($UP_{sol}1$ and $UP_{sol}2$ fractions, respectively).
● In this experiment, the following extraction steps were equivalent to those described in Figure 1A.

Step 1: extraction with PBS ($UP_{sol}1$);
Step 2: extraction with PBS + 50 mM DTT ($UP_{sol}2a$);
Step 3: re-extraction with PBS + 50 mM DTT ($UP_{sol}2b$);
Step 4: incubation/extraction with PBS and DNAse I ($UP_{sol}3$);
Step 5: extraction/ incubation with lysozyme, EDTA and CHAPS ($UP_{sol}4$)
Step 6: incubation/heat extraction with denaturing solution ($UP_{sol}5$);

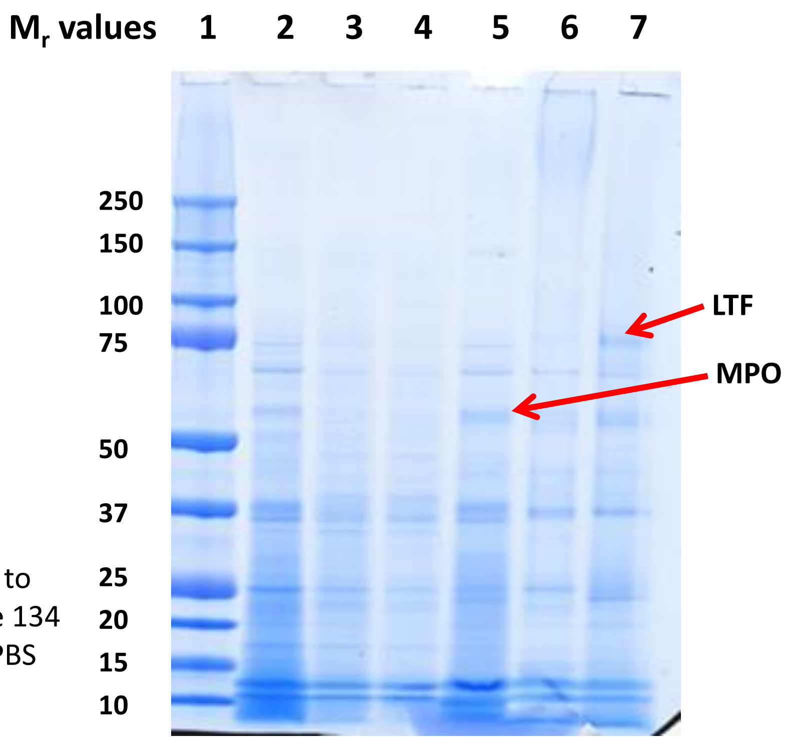

Figure 2I

Date: 11-03-15
NuPAGE gel, 4-12% acrylamide, Coomassie Brilliant Blue G250 stained
<u>AUP sample 134</u>
(sample was subjected to two freeze/thaw cycles prior to extraction of protein and DNA)

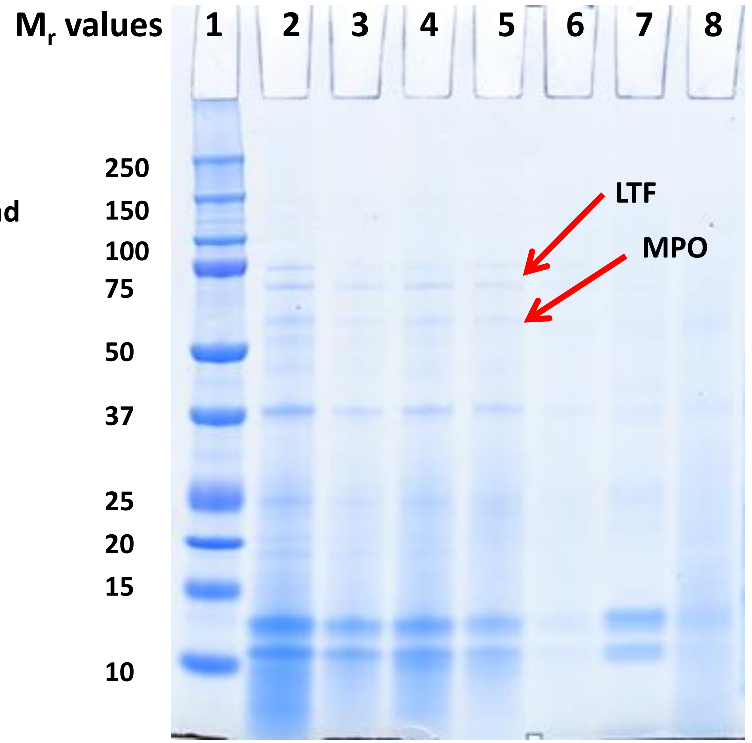

1: M$_r$ standard, 5 ul solution
2: UP$_{sol}$1, ca. 11 ul extract
3: UP$_{sol}$2a, ca. 11 ul extract
4: UP$_{sol}$2b, ca. 11 ul extract
5: UP$_{sol}$3a, ca. 11 ul extract; 6: wash step with PBS, ca. 11 ul extract
7: UP$_{sol}$4, ca. 11 ul extract
8: UP$_{sol}$5, ca. 11 ul extract

● Sample 134 was viscous and clumpy. *In vitro* culture and proteomic analysis revealed *Klebsiella pneumoniae* as the infectious agent. The extractions related to this experiment were performed after two freeze/thaw cycles. The pellet of sample 134 became less clumpy and viscous but was not completely homogenized upon incubation with PBS and PBS + 50 mM DTT (UP$_{sol}$1 and UP$_{sol}$2 fractions, respectively). Homogenization was achieved with the DNAse I incubation step.
● In this experiment, the following extraction steps were equivalent to those described in Figure 1A, except that an additional wash step after DNAse I incubation was added.

Summary:
Step 1: extraction with PBS (UP$_{sol}$1);
Step 2: two extraction steps with PBS + 50 mM DTT (UP$_{sol}$2a and UP$_{sol}$2b);
Step 3: incubation/extraction with PBS and DNAse I (UP$_{sol}$3);
Step 4: pellet wash step with PBS;
Step 5: extraction/ incubation with lysozyme, EDTA and CHAPS (UP$_{sol}$4);
Step 6: incubation/heat extraction with denaturing solution (UP$_{sol}$5);

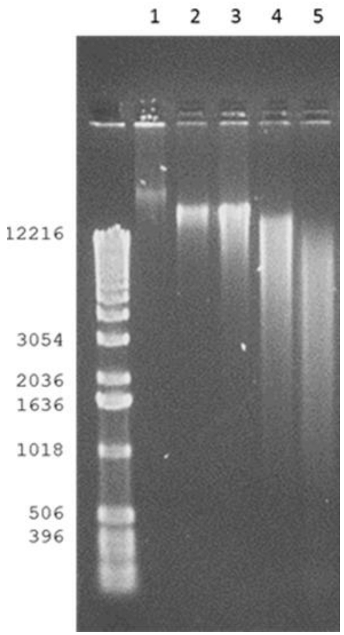

Date: 10-30-15
0.5% agarose gel, ethidium bromide stained
Sample 134

left: 5: DNA standard, 1 ul solution
1: UP$_{sol}$1, ca. 1 ul extract
2: UP$_{sol}$2a, ca. 1 ul extract
3: UP$_{sol}$2b, ca. 1 ul extract
4: UP$_{sol}$3a, ca. 1 ul, DNAse I activity stopped after 10 min
5: UP$_{sol}$3, ca. 1 ul, DNAse I activity stopped after 45 min

Figure 2m

Date: 04-30-15
NuPAGE gel, 4-12% acrylamide, Coomassie Brilliant Blue G250 stained
<u>AUP sample 20</u>

1: M_r standard, 5 ul solution
2: $UP_{sol}1$, ca. 10 ul extract
3: $UP_{sol}2$, ca. 10 ul extract
4: $UP_{sol}3$, ca. 10 ul extract
5: $UP_{sol}5$, ca. 10 ul extract

● Sample 20 was originally viscous and clumpy. *In vitro* culture and proteomic analysis revealed *Proteus mirabilis* as the infectious agent. The extractions related to this experiment were performed after two freeze/thaw cycles. The pellet of sample 20 was less clumpy and viscous upon thawing for the 2nd time. It was homogenized upon incubation with PBS and PBS + 50 mM DTT ($UP_{sol}1$ and $UP_{sol}2$ fractions, respectively). DNA was released into the $UP_{sol}1$ and $UP_{sol}2$ fractions. No further homogenization was achieved with the DNAse I incubation step ($UP_{sol}3$ fraction). The DNA extraction was performed after a 3rd freeze-thaw cycle.
● In this experiment, the following extraction steps were equivalent to those described in Figure 1A, with that the extraction step resulting in $UP_{sol}4$ was not performed.

Summary:
Step 1: extraction with PBS ($UP_{sol}1$);
Step 2: extraction with PBS + 50 mM DTT ($UP_{sol}2$);
Step 3: incubation/extraction with PBS and DNAse I ($UP_{sol}3$);
Step 4: incubation/heat extraction with denaturing solution ($UP_{sol}5$);

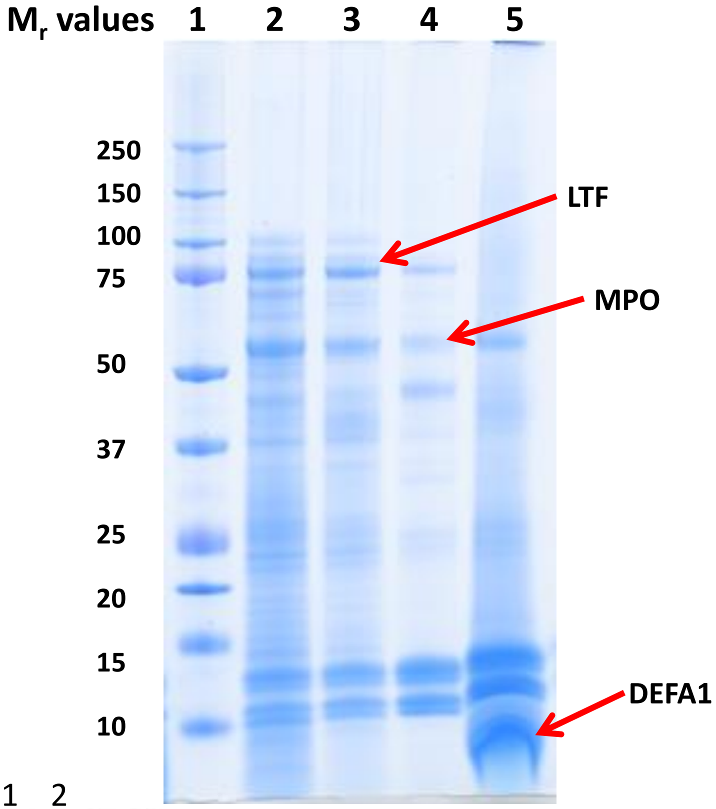

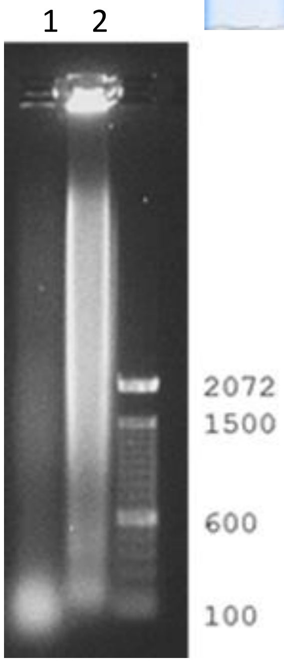

Date: 10-28-15
0.5% agarose gel, ethidium bromide stained
Sample 20

1: $UP_{sol}1$, ca. 1 ul extract, after a 45 min incubation with DNAse I
2: $UP_{sol}1$, ca. 1 ul extract
right: 5: DNA standard, 1 ul solution

Figure 2n

Date: 09-26-15
NuPAGE gel, 4-12% acrylamide, Coomassie Brilliant Blue G250 stained
<u>AUP sample 122</u>
(sample was not subjected to freeze/thaw cycle prior to extraction)

1: $UP_{sol}1$, ca. 10 ul extract
2: $UP_{sol}2$, ca. 10 ul extract
3: $UP_{sol}3$, ca. 10 ul extract
4: $UP_{sol}5$, ca. 10 ul extract
5: M_r standard, 10 ul solution

● Sample 122 was viscous and clumpy. *In vitro* culture and proteomic analysis revealed *Candida albicans* as the infectious agent. The extractions related to this experiment were performed without freezing. The pellet of sample 122 remained clumpy and viscous and was not homogenized upon incubation with PBS and PBS + 50 mM DTT ($UP_{sol}1$ and $UP_{sol}2$ fractions, respectively).
● In this experiment, the following extraction steps were equivalent to those described in Figure 1A, with that the extraction step resulting in $UP_{sol}4$ was not performed.

Summary:
Step 1: extraction with PBS ($UP_{sol}1$);
Step 2: extraction with PBS + 50 mM DTT ($UP_{sol}2$);
Step 3: incubation/extraction with PBS and DNAse I ($UP_{sol}3$);
Step 4: incubation/heat extraction with denaturing solution ($UP_{sol}5$);

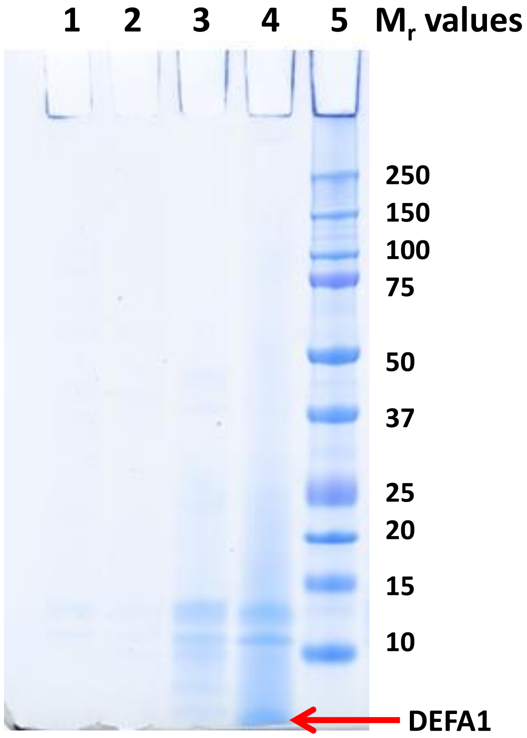

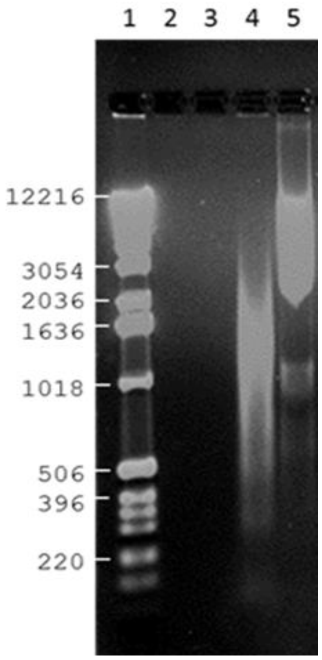

Date: 09-30-15
0.5% agarose gel, ethidium bromide stained
Sample 122

1: 5: DNA standard, 1 ul solution
2: $UP_{sol}1$, ca. 1 ul extract
3: $UP_{sol}2$, ca. 1 ul extract
4: $UP_{sol}3$, ca. 1 ul extract
5: $UP_{sol}5$, ca. 1 ul extract

Figure 2o

Date: 10-13-15
NuPAGE gel, 4-12% acrylamide, Coomassie Brilliant Blue G250 stained
<u>AUP sample 124</u>
(sample was not subjected to freeze/thaw cycle prior to extraction)

1: M_r standard, 5 ul solution
2: $UP_{sol}1$, ca. 15 ul extract
3: $UP_{sol}2$, ca. 15 ul extract
4: $UP_{sol}3$, ca. 15 ul extract
5: $UP_{sol}4$, ca. 15 ul extract
6: $UP_{sol}5$, ca. 15 ul extract

● Sample 124 was clumpy and had a very large volume. *In vitro* culture amd microscopy revealed *Candida albicans* as the infectious agent. The extractions related to this experiment were performed without freezing. The pellet of sample 124 remained clumpy, but was partially homogenized upon incubation with PBS + 50 mM DTT ($UP_{sol}1$ and $UP_{sol}2$ fractions, respectively).
● In this experiment, the following extraction steps were equivalent to those described in Figure 1A.

Summary:
Step 1: extraction with PBS ($UP_{sol}1$)
Step 2: extraction with PBS + 50 mM DTT ($UP_{sol}2a$);
Step 3: incubation/extraction with PBS and DNAse I ($UP_{sol}3$);
Step 4: extraction/ incubation with lysozyme, EDTA and CHAPS ($UP_{sol}4$)
Step 6: incubation/heat extraction with denaturing solution ($UP_{sol}5$);

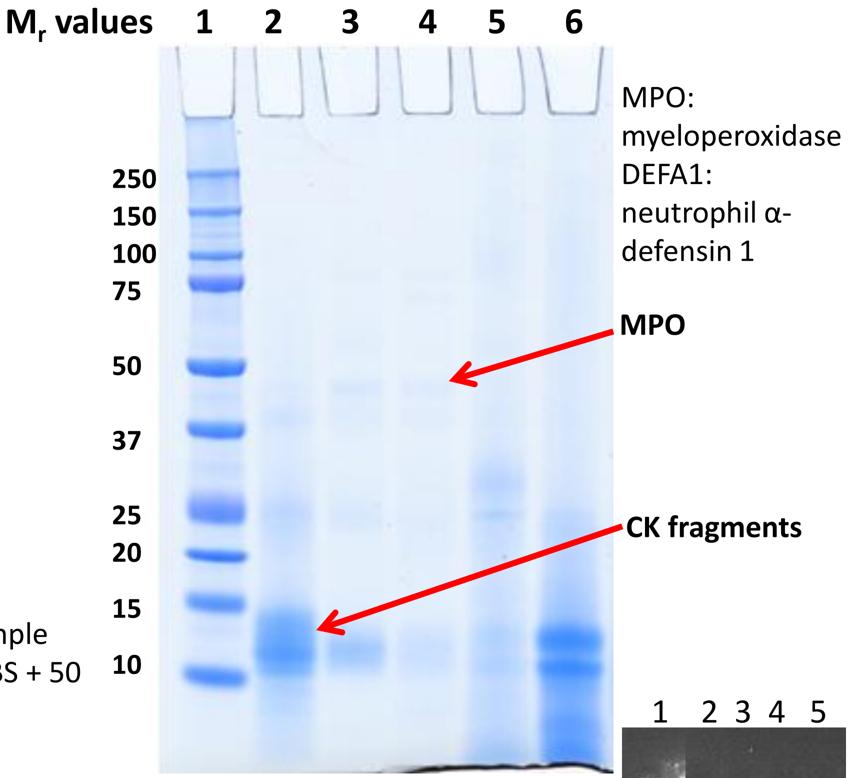

Date: 06-22-16
0.5% agarose gel, ethidium bromide stained
Sample 124

1: DNA standard, 1 ul solution
2: $UP_{sol}1$, ca. 3 ul extract
3: $UP_{sol}2$, ca. 3 ul extract
4: $UP_{sol}3$, ca. 3 ul extract, incubation stopped with EDTA after 10 min
5: $UP_{sol}3$, ca. 3 ul extract, incubation stopped with EDTA after 60 min

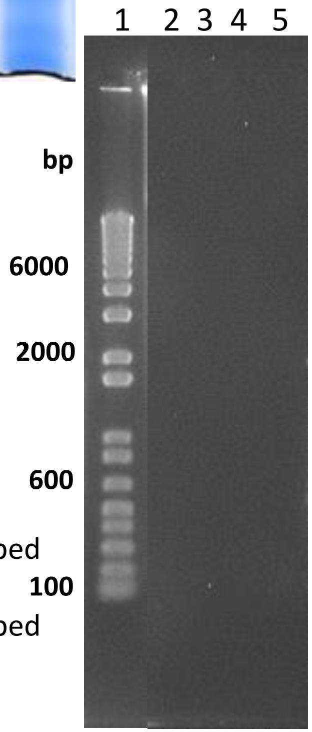

Figure 2p

Date: 07-12-16
NuPAGE gel, 4-12% acrylamide, Coomassie Brilliant Blue G250 stained
AUP sample 142

1: $UP_{sol}1$, ca. 10 ul extract
2: $UP_{sol}2$, ca. 10 ul extract
3: $UP_{sol}3$, ca. 10 ul extract, 10 min DNAse incubation
4: $UP_{sol}3$, ca. 10 ul extract , 60 min DNAse incubation
5: $UP_{sol}5$, ca. 10 ul extract
6: M_r standard, 10 ul solution

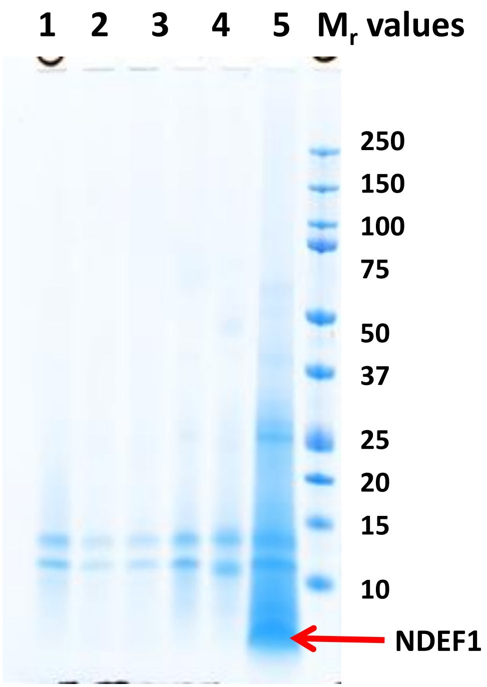

UP sample 142 was not clumpy. In vitro culture and proteomic analysis revealed *Serratia marcescens* as the infectious agent. The extractions related to this experiment were performed from a UP sample obtained approximately 2-5 hours after urine specimen collection without cooling or freezing. The UP sample 142 became clumpy and aggregated after centrifugation and addition of PBS + 50 mM DTT. The aggregate was not homogenized upon incubation with PBS and PBS + 50 mM DTT ($UP_{sol}1$ and $UP_{sol}2$ fractions, respectively). The pellet derived from $UP_{sol}2$ extraction was incubated with DNAse I for 10 min, and then another 50 min, resulting in fraction $UP_{sol}3$.

In this experiment, the following extraction steps were identical to those described in Figure 1A, with the exception that the extraction step resulting in $UP_{sol}4$ was not performed.

Summary:
Step 1: extraction with PBS ($UP_{sol}1$);
Step 2: extraction with PBS + 50 mM DTT ($UP_{sol}2$);
Step 3: incubation/extraction with PBS and DNAse I ($UP_{sol}3$);
Step 4: incubation/heat extraction with denaturing solution ($UP_{sol}5$);

Date: 07-15-16
0.5% agarose gel, ethidium bromide stained
Sample 142

1: DNA standard, 1 ul solution
2: $UP_{sol}1$, ca. 3 ul extract
3: $UP_{sol}2$, ca. 3 ul extract
4: $UP_{sol}3$, ca. 3 ul extract, incubation stopped with EDTA after 60 min

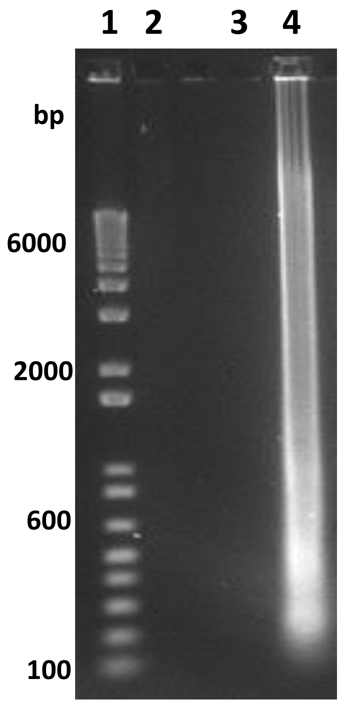

Figure 2q

Date: 07-19-16
NuPAGE gel, 4-12% acrylamide, Coomassie Brilliant Blue G250 stained
<u>AUP sample 151</u>
(sample was not subjected to freeze/thaw cycle prior to extraction)

1: $UP_{sol}1$, ca. 10 ul extract
2: $UP_{sol}2$, ca. 10 ul extract
3: $UP_{sol}3$, ca. 10 ul extract
4: $UP_{sol}5$, ca. 10 ul extract
5: M_r standard, 5 ul solution

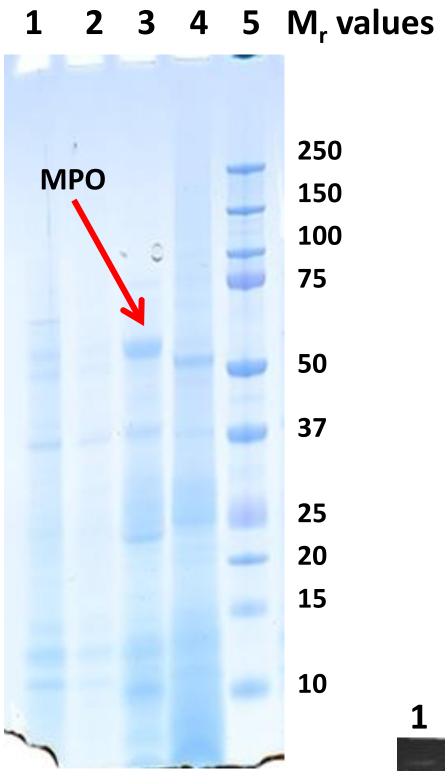

- Sample 151 was aggregated and clumpy. In vitro culture and proteomic analysis revealed *Escherichia coli* as the infectious agent. The extractions related to this experiment were performed from a UP sample obtained approximately 2-5 hours after urine specimen collection without cooling or freezing the neutrophils. The pellet of sample 151 remained clumpy and was not homogenized upon incubation with PBS and PBS + 50 mM DTT ($UP_{sol}1$ and $UP_{sol}2$ fractions, respectively). The pellet derived from $UP_{sol}2$ extraction was incubated with DNAse I for 60 min, resulting in fraction $UP_{sol}3$.
- In this experiment, the following extraction steps were identical to those described in Figure 1A, with the exception that the extraction step resulting in $UP_{sol}4$ was not performed.

Summary:
Step 1: extraction with PBS ($UP_{sol}1$);
Step 2: extraction with PBS + 50 mM DTT ($UP_{sol}2$);
Step 3: incubation/extraction with PBS and DNAse I ($UP_{sol}3$);
Step 4: incubation/heat extraction with denaturing solution ($UP_{sol}5$);

Date: 07-15-16
0.5% agarose gel, ethidium bromide stained
Sample 151

1: DNA standard, 1 ul solution
2: $UP_{sol}1$, ca. 3 ul extract
3: $UP_{sol}2$, ca. 3 ul extract
4: $UP_{sol}3$, ca. 3 ul extract, incubation stopped with EDTA after 10 min
5: $UP_{sol}3$, ca. 3 ul extract, incubation stopped with EDTA after 60 min

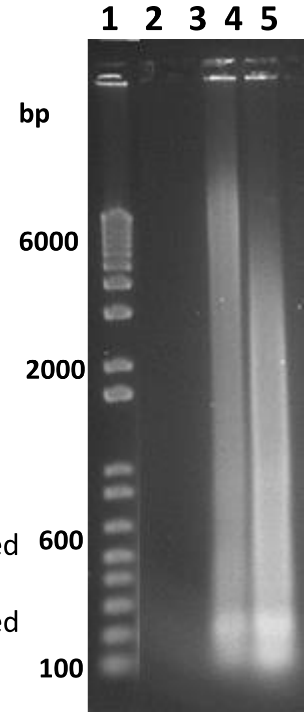

Figure 2r

Date: 07-19-16
NuPAGE gel, 4-12% acrylamide, Coomassie Brilliant Blue G250 stained
AUP sample 157
(sample was not subjected to freeze/thaw cycle prior to extraction)

1: M_r standard, 10 ul solution
2: $UP_{sol}1$, ca. 10 ul extract
3: $UP_{sol}2$, ca. 10 ul extract
4: $UP_{sol}3$, ca. 10 ul extract
5: $UP_{sol}5$, ca. 10 ul extract

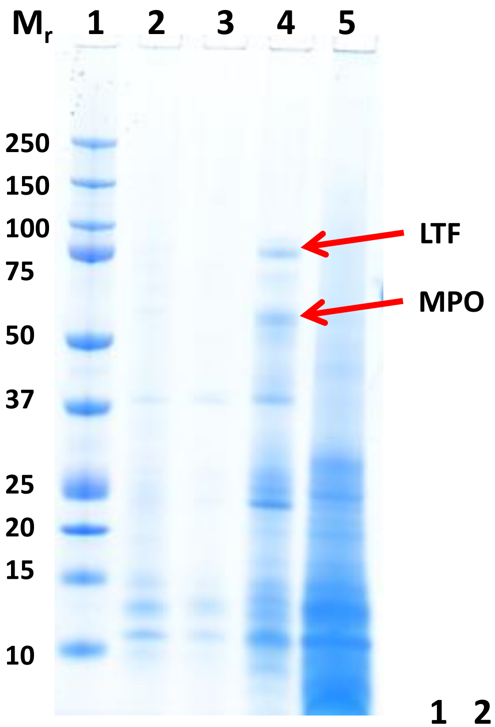

● Sample 157 was aggregated, moderately viscous and clumpy. In vitro culture and proteomic analysis revealed *Staphylococcus aureus* as the infectious agent. The extractions related to this experiment were performed from a UP sample obtained approximately 2-5 hours after urine specimen collection without cooling or freezing. The pellet of sample 157 remained clumpy and viscous and was not homogenized upon incubation with PBS and PBS + 50 mM DTT ($UP_{sol}1$ and $UP_{sol}2$ fractions, respectively). The pellet derived from $UP_{sol}2$ extraction was incubated with DNAse I for 60 min, resulting in fraction $UP_{sol}3$.
● In this experiment, the following extraction steps were identical to those described in Figure 1A, with the exception that the extraction step resulting in $UP_{sol}4$ was not performed.

Summary:
Step 1: extraction with PBS ($UP_{sol}1$);
Step 2: extraction with PBS + 50 mM DTT ($UP_{sol}2$);
Step 3: incubation/extraction with PBS and DNAse I ($UP_{sol}3$);
Step 4: incubation/heat extraction with denaturing solution ($UP_{sol}5$);

Date: 07-15-16
0.5% agarose gel, ethidium bromide stained
Sample 157

1: DNA standard, 1 ul solution
2: $UP_{sol}1$, ca. 3 ul extract
3: $UP_{sol}2$, ca. 3 ul extract
4: $UP_{sol}3$, ca. 3 ul extract, incubation stopped with EDTA after 10 min
5: $UP_{sol}3$, ca. 3 ul extract, incubation stopped with EDTA after 60 min

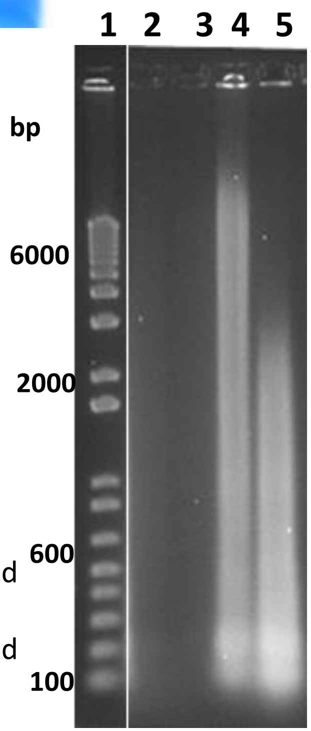

Figure 2s

Date: 07-07-16
NuPAGE gel, 4-12% acrylamide, Coomassie Brilliant Blue G250 stained
<u>AUP sample 136</u>

1: $UP_{sol}1$, ca. 10 ul extract
2: $UP_{sol}2$, ca. 10 ul extract
3: $UP_{sol}3$, ca. 10 ul extract , 60 min DNAse incubation
4: $UP_{sol}5$, ca. 10 ul extract
5: M_r standard, 10 ul solution

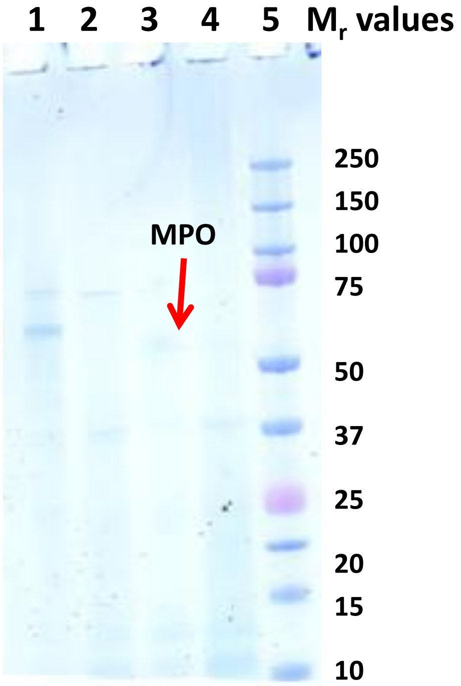

- Sample 136 was aggregated and clumpy. In vitro culture and proteomic analysis revealed *Escherichia coli* as the infectious agent. The extractions related to this experiment were performed from a UP sample obtained approximately 2-5 hours after urine specimen collection without cooling or freezing the neutrophils. The pellet of sample 136 remained clumpy and was not homogenized upon incubation with PBS and PBS + 50 mM DTT ($UP_{sol}1$ and $UP_{sol}2$ fractions, respectively). The pellet derived from $UP_{sol}2$ extraction was incubated with DNAse I for 60 min, resulting in fraction $UP_{sol}3$.
- In this experiment, the following extraction steps were identical to those described in Figure 1A, with the exception that the extraction step resulting in $UP_{sol}4$ was not performed.

Summary:
Step 1: extraction with PBS ($UP_{sol}1$);
Step 2: extraction with PBS + 50 mM DTT ($UP_{sol}2$);
Step 3: incubation/extraction with PBS and DNAse I ($UP_{sol}3$);
Step 4: incubation/heat extraction with denaturing solution ($UP_{sol}5$);

Date: 07-15-16
0.5% agarose gel, ethidium bromide stained
UP sample 136

1: DNA standard, 1 ul solution
2: $UP_{sol}1$, ca. 3 ul extract
3: $UP_{sol}2a$, ca. 3 ul extract
4: $UP_{sol}2b$, ca. 3 ul extract
5: $UP_{sol}3$, ca. 3 ul extract, incubation stopped with EDTA after 60 min

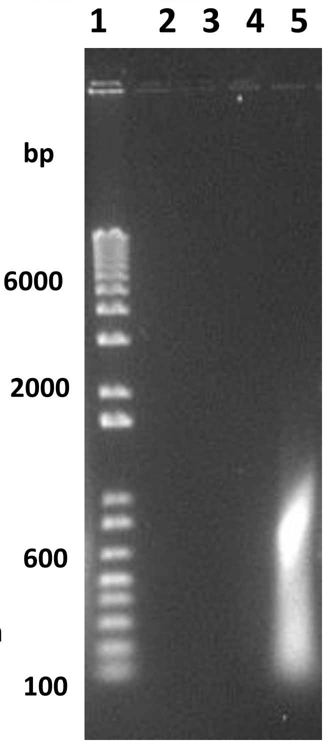

Figure 2t

Date: 08-30-16
NuPAGE gel, 4-12% acrylamide, Coomassie Brilliant Blue G250 stained
<u>AUP sample 146</u>

1: $UP_{sol}1$, ca. 10 ul extract
2: $UP_{sol}2$, ca. 10 ul extract
3: $UP_{sol}3$, ca. 10 ul extract, 10 min DNAse incubation
4: $UP_{sol}3$, ca. 10 ul extract , 60 min DNAse incubation
5: $UP_{sol}5$, ca. 10 ul extract
6: M_r standard, 10 ul solution

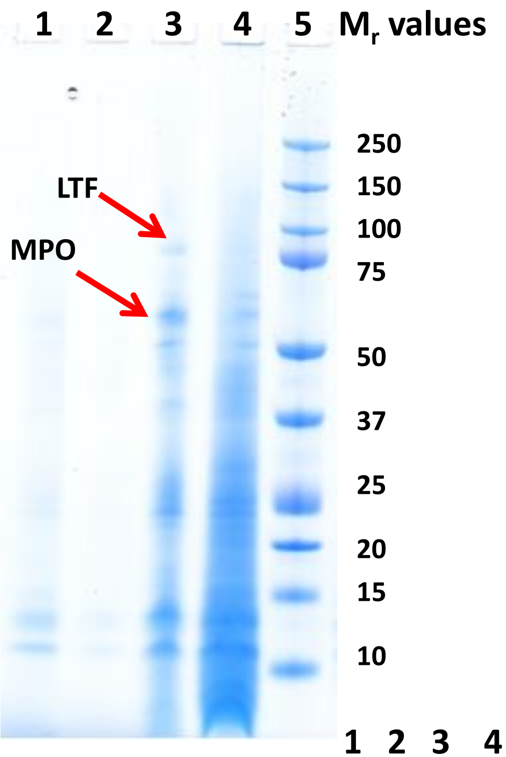

● Sample 146 was aggregated and clumpy. In vitro culture and proteomic analysis revealed *Escherichia coli* as the infectious agent. The extractions related to this experiment were performed from a UP sample obtained approximately 2-5 hours after urine specimen collection without cooling or freezing the neutrophils. The pellet of sample 146 remained clumpy and was not homogenized upon incubation with PBS and PBS + 50 mM DTT ($UP_{sol}1$ and $UP_{sol}2$ fractions, respectively). The pellet derived from $UP_{sol}2$ extraction was incubated with DNAse I for 60 min, resulting in fraction $UP_{sol}3$.

● In this experiment, the following extraction steps were identical to those described in Figure 1A, with the exception that the extraction step resulting in $UP_{sol}4$ was not performed.

Summary:
Step 1: extraction with PBS ($UP_{sol}1$);
Step 2: extraction with PBS + 50 mM DTT ($UP_{sol}2$);
Step 3: incubation/extraction with PBS and DNAse I ($UP_{sol}3$);
Step 4: incubation/heat extraction with denaturing solution ($UP_{sol}5$);

Date: 07-15-16
0.5% agarose gel, ethidium bromide stained
Sample 146

1: DNA standard, 1 ul solution
2: $UP_{sol}1$, ca. 3 ul extract
3: $UP_{sol}2a$, ca. 3 ul extract
4: $UP_{sol}2b$, ca. 3 ul extract
5: $UP_{sol}3$, ca. 3 ul extract, incubation stopped with EDTA after 60 min

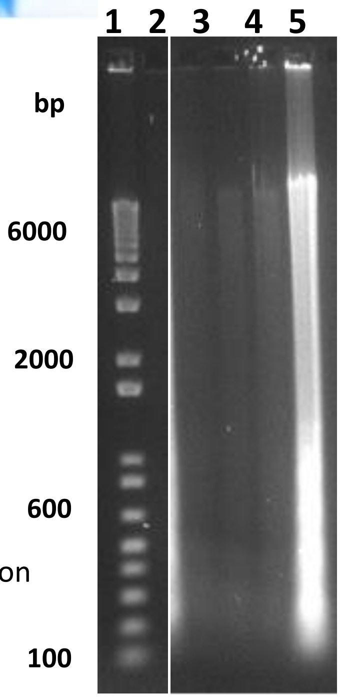

Figure 3. Western blot confirming the reactivity of S. aureus protein A (Spa) with anti-rabbit IgG HRP conjugate

Date: 08-30-16
NuPAGE gel, 4-12% acrylamide followed by western blot transfer to PVDF membrane

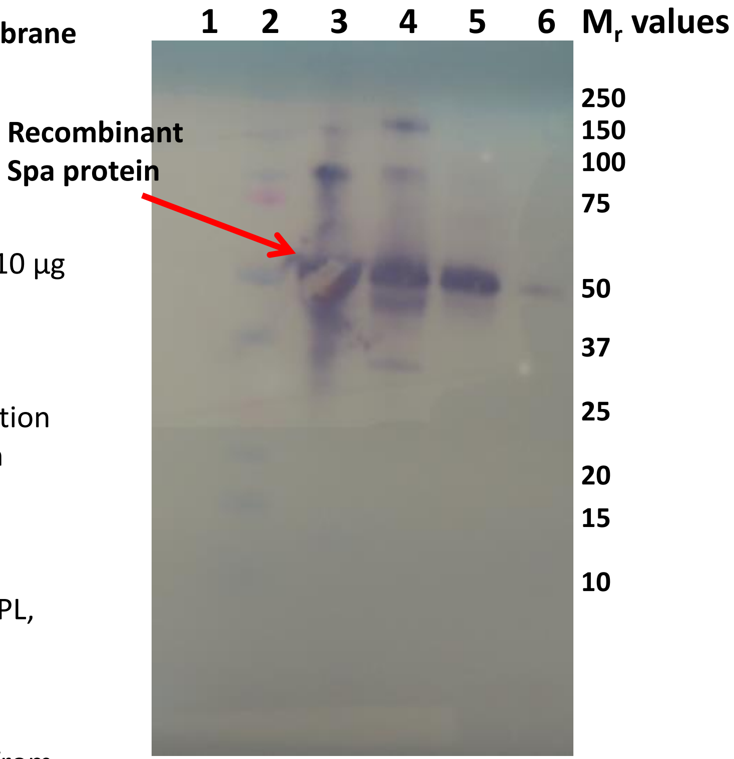

1: $UP_{sol}3$ from lysate of AUP sample 134, ca. 10 ul extract
2: Mr standard, 10 ul solution
3: recombinant S. aureus protein A (RPA-50, Cat. No. 1001-01, Repligen), 0.1 µg
4: lysostaphin digest of cell extract from S. aureus AUP sample #112 isolate, ca. 10 µg
5: lysostaphin digest of cell extract from S. aureus strain HIP5827, ca. 10 µg
6: $UP_{sol}3$ from lysate of AUP sample 112, ca. 10 ul extract

● The blot was performed using no primary antibody dilution (instead an incubation with 1% BSA in PBS-Tween 20, 0.05%, for 90 min was performed), followed by an incubation with a secondary antibody HRP conjugate (horseradish peroxidase conjugate of goat anti-rabbit IgG-HRP; Sta. Cruz Biotech, sc-2004) in a 1:10,000 dilution in 1% BSA in PBS-Tween 20, 0.05%, for 90 min and detection of protein recognition by 2nd antibody using the substrate 3,3',5,5'-Tetramethylbenzidine (KPL, Gaithersburg, MD; #54-11-50) in a colorimetric endpoint assay over 4 min.

Summary:
The reactivity of a major band at 50 kDa indicates that *S. aureus* protein A (Spa) from the recombinant protein (lane 3), two lysostaphin digests of S. aureus strains (lanes 4 and 5) and the $UP_{sol}3$ fraction of AUP sample #112 is recognized by the 2nd antibody-HRP conjugate. In conclusion, Spa from the clinical sample (AUP sample #112) actively binds IgG which is a relevant activity in the defense against immunoglobulins and opsonization.
