# Supplementary material for: Characterization of Early-Phase Neutrophil Extracellular Traps in Urinary Tract Infections
Source: PLoS Pathog. 2017 Jan 27;13(1):e1006151. doi: 10.1371/journal.ppat.1006151 (PMC5298345; doi:10.1371/journal.ppat.1006151)

Supplementary Data, Dataset S4: Citrullinated peptides of proteins isolated from AUP samples

MS/MS data consistent with citrullination of proteins

- citrullination on arginine (R) will cause a mass increase of 0.98 Da:
- neutral losses considered;
- matched peptides containing N/Q were excluded if fragmentation spectra did not allow differentiating between deamidation of N/Q and deamidation of R residues; all spectra were manually inspected

This data is derived from shotgun proteomic datasets using the Q-Exactive mass spectrometer and Proteome Discoverer or MaxQuant analysis software tools to assess a peptide citrullination site deamidation of arginine); MS methods are described in the method section of the main text.
Sequest HT/Proteome Discoverer: slides 1-6, 8-10, 12-32
Andromeda/MaxQuant: slides 7 and 11

AUP sample #157

Accession	Description
P16403	Histone H1.2 - [H12_HUMAN]

Sequence	Modifications	XCorr	Charge	MH+ [Da]	ΔM [ppm]	# Missed Cleavages
AGGTPrK	R6(Deamidated)	2.04	2	687.3762	-3.36	1

#1	b^+	b^{2+}	Seq.	y^+	y^{2+}	#2
1	72.04440	36.52584	A			7
2	129.06587	65.03657	G	616.34134	308.67431	6
3	186.08734	93.54731	G	559.31987	280.16357	5
4	287.13502	144.07115	T	502.29840	251.65284	4
5	384.18779	192.59753	P	401.25072	201.12900	3
6	541.27292	271.14010	R-Deamid...	304.19795	152.60261	2
7			K	147.11281	74.06004	1

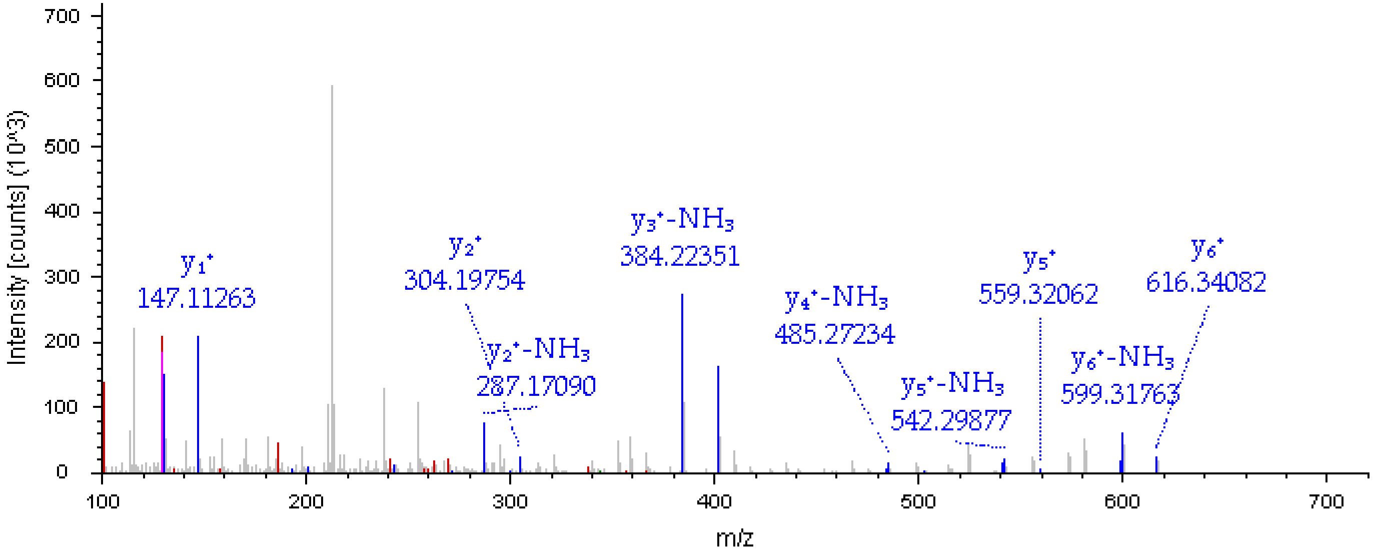

AUP sample #157

Accession	Description
P06748	Nucleophosmin - [NPM_HUMAN]

Sequence	Modifications	XCorr	Charge	MH+ [Da]	ΔM [ppm]	# Missed Cleavages
rSAPGGGSK	R1(Deamidated)	1.77	2	817.4139	-2.91	1

#1	b^+	b^{2+}	Seq.	y^+	y^{2+}	#2
1	158.09241	79.54984	R-Deamid...			9
2	245.12444	123.06586	S	660.33117	330.66922	8
3	316.16156	158.58442	A	573.29914	287.15321	7
4	413.21433	207.11080	P	502.26202	251.63465	6
5	470.23580	235.62154	G	405.20925	203.10826	5
6	527.25727	264.13227	G	348.18778	174.59753	4
7	584.27874	292.64301	G	291.16631	146.08679	3
8	671.31077	336.15902	S	234.14484	117.57606	2
9			K	147.11281	74.06004	1

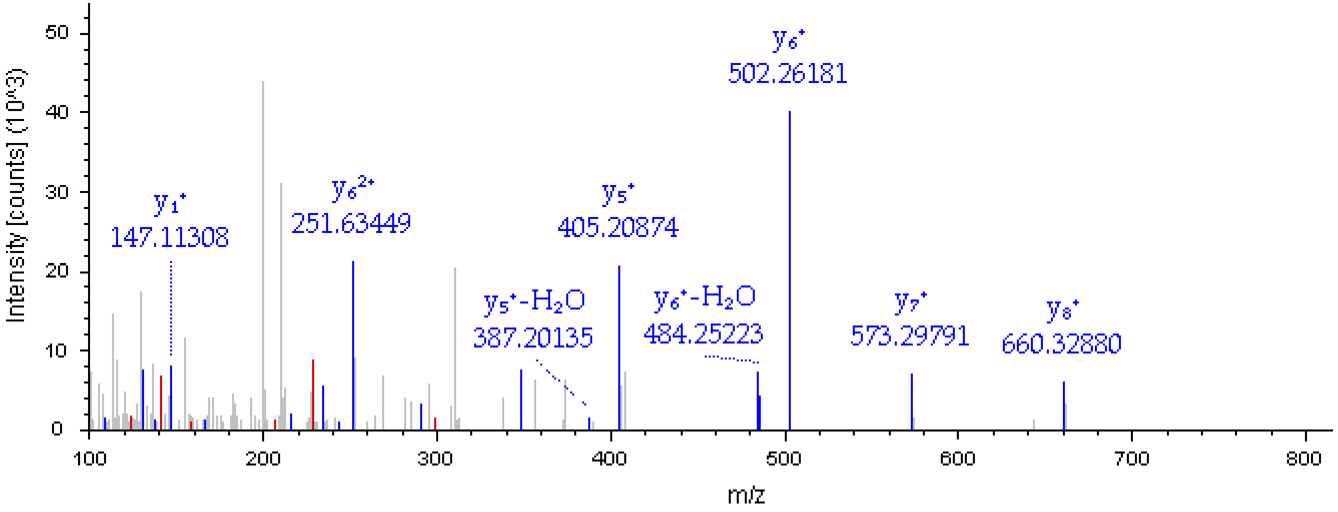

AUP sample #157

Accession	Description
P38159	RNA-binding motif protein, X chromosome - [RBMX_HUMAN]

Sequence	Modifications	XCorr	Charge	MH+ [Da]	ΔM [ppm]	# Missed Cleavages
GPPPrSGGPPPK	R5(Deamidated)	1.49	2	1144.6076	-2.98	1

#1	Immonium	a+	b+	Seq.	y+	#2
1	30.03383	30.03383	58.02875	G		12
2	70.06513	127.08660	155.08152	P	1087.58954	11
3	70.06513	224.13937	252.13429	P	990.53677	10
4	70.06513	321.19214	349.18706	P	893.48400	9
5	129.11348	478.27728	506.27219	R-Deamid...	796.43123	8
6	60.04439	565.30931	593.30422	S	639.34609	7
7	30.03383	622.33078	650.32569	G	552.31406	6
8	30.03383	679.35225	707.34716	G	495.29259	5
9	70.06513	776.40502	804.39993	P	438.27112	4
10	70.06513	873.45779	901.45270	P	341.21835	3
11	70.06513	970.51056	998.50547	P	244.16558	2
12				K	147.11281	1

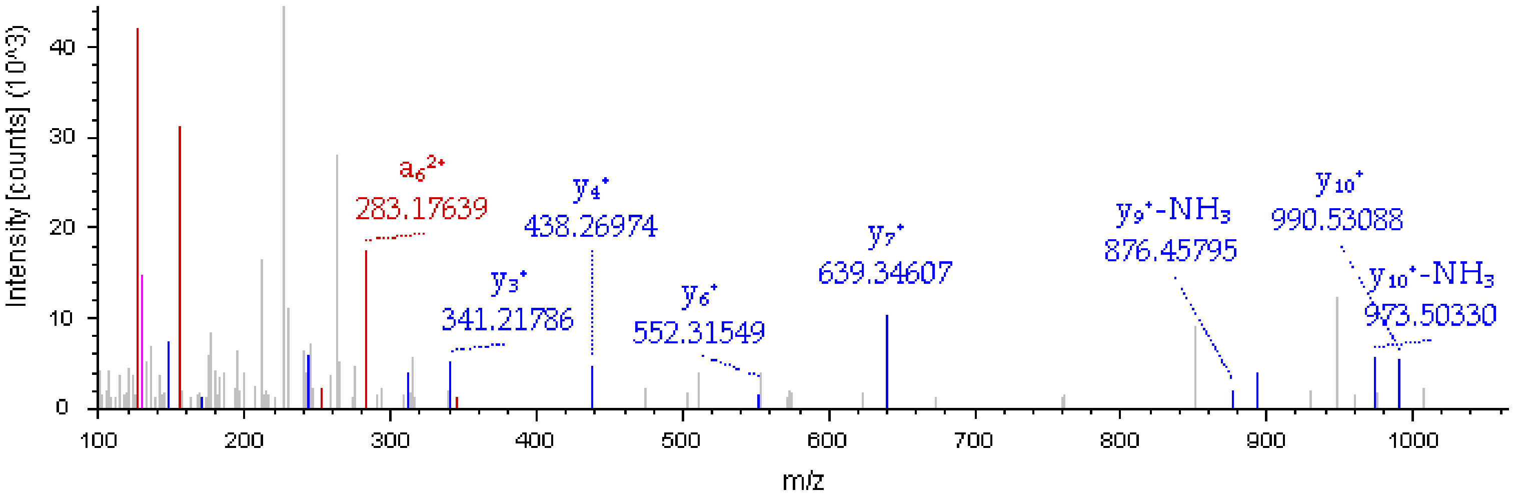

AUP sample #157

Sequence: IDSGLYLGSGYFTAIQNLR, R19-Deamidated (0.98402 Da)
Charge: +2, Monoisotopic m/z: 1045.03784 Da (+4.15 mmu/+3.97 ppm), MH+: 2089.06841 Da, RT: 49.83 min,
Identified with: Sequest HT (v1.3); XCorr:2.33, Ions matched by search engine: 0/0
Fragment match tolerance used for search: 0.02 Da
Fragments used for search: b; b-H_2O; b-NH_3; y; y-H_2O; y-NH_3

Protein references (1):
- Lactotransferrin OS=Homo sapiens GN=LTF PE=1 SV=6 - [TRFL_HUMAN]

#1	Immonium	a^+	a^{2+}	b^+	b^{2+}	Seq.	y^+	y^{2+}	#2
1	86.09643	86.09643	43.55185	114.09135	57.54931	I			19
2	88.03931	201.12338	101.06533	229.11830	115.06279	D	1975.97605	988.49166	18
3	60.04439	288.15541	144.58134	316.15033	158.57880	S	1860.94910	930.97819	17
4	30.03383	345.17688	173.09208	373.17180	187.08954	G	1773.91707	887.46217	16
5	86.09643	458.26095	229.63411	486.25587	243.63157	L	1716.89560	858.95144	15
6	136.07568	621.32427	311.16577	649.31919	325.16323	Y	1603.81153	802.40940	14
7	86.09643	734.40834	367.70781	762.40326	381.70527	L	1440.74821	720.87774	13
8	30.03383	791.42981	396.21854	819.42473	410.21600	G	1327.66414	664.33571	12
9	60.04439	878.46184	439.73456	906.45676	453.73202	S	1270.64267	635.82497	11
10	30.03383	935.48331	468.24529	963.47823	482.24275	G	1183.61064	592.30896	10
11	136.07568	1098.54663	549.77695	1126.54155	563.77441	Y	1126.58917	563.79822	9
12	120.08078	1245.61505	623.31116	1273.60997	637.30862	F	963.52585	482.26656	8
13	74.06004	1346.66273	673.83500	1374.65765	687.83246	T	816.45743	408.73235	7
14	44.04948	1417.69985	709.35356	1445.69477	723.35102	A	715.40975	358.20851	6
15	86.09643	1530.78392	765.89560	1558.77884	779.89306	I	644.37263	322.68995	5
16	101.07094	1658.84250	829.92489	1686.83742	843.92235	Q	531.28856	266.14792	4
17	87.05529	1772.88543	886.94635	1800.88035	900.94381	N	403.22998	202.11863	3
18	86.09643	1885.96950	943.48839	1913.96442	957.48585	L	289.18705	145.09716	2
19						R-Deamid...	176.10298	88.55513	1

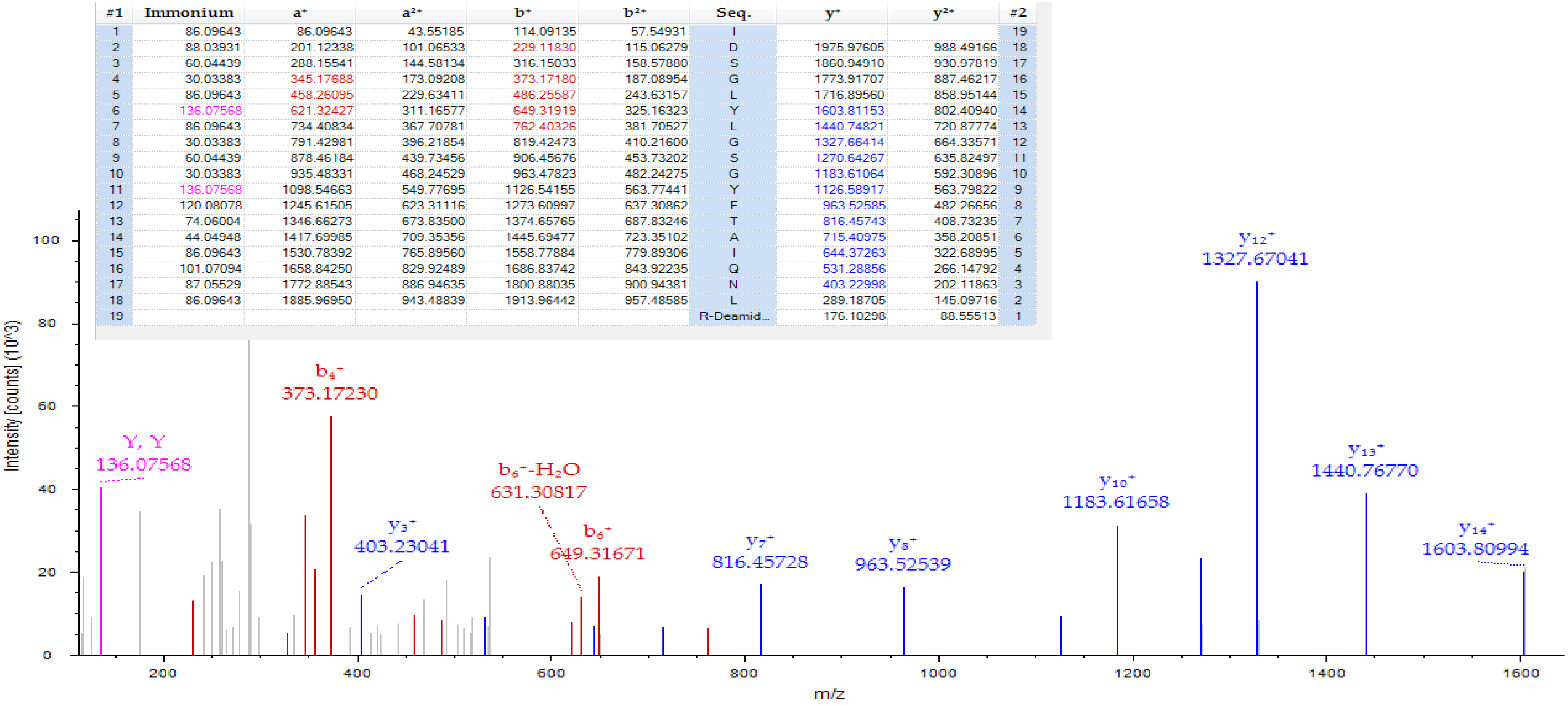

AUP sample #112

Sequence: VARKSAPATGGVK, R3-Deamidated (0.98402 Da)
Charge: +3, Monoisotopic m/z: 414.91092 Da (+0.56 mmu/+1.34 ppm), MH+: 1242.71820 Da, RT: 13.79 min,
Identified with: Sequest HT (v1.3); XCorr:2.47, Ions matched by search engine: 0/0
Fragment match tolerance used for search: 0.02 Da
Fragments used for search: b; b-H_2O; b-NH_3; y; y-H_2O; y-NH_3

Protein references (1):
- Histone H3.1t OS=Homo sapiens GN=HIST3H3 PE=1 SV=3 - [H31T_HUMAN]

#1	Immonium	a^+	a^{2+}	b^+	b^{2+}	Seq.	y^+	y^{2+}	#2
1	72.08078	72.08078	36.54403	100.07570	50.54149	V			13
2	44.04948	143.11790	72.06259	171.11282	86.06005	A	1143.64812	572.32770	12
3	129.11348	300.20304	150.60516	328.19795	164.60261	R-Deamid...	1072.61100	536.80914	11
4	101.10733	428.29801	214.65264	456.29292	228.65010	K	915.52586	458.26657	10
5	60.04439	515.33004	258.16866	543.32495	272.16611	S	787.43089	394.21908	9
6	44.04948	586.36716	293.68722	614.36207	307.68467	A	700.39886	350.70307	8
7	70.06513	683.41993	342.21360	711.41484	356.21106	P	629.36174	315.18451	7
8	44.04948	754.45705	377.73216	782.45196	391.72962	A	532.30897	266.65812	6
9	74.06004	855.50473	428.25600	883.49964	442.25346	T	461.27185	231.13956	5
10	30.03383	912.52620	456.76674	940.52111	470.76419	G	360.22417	180.61572	4
11	30.03383	969.54767	485.27747	997.54258	499.27493	G	303.20270	152.10499	3
12	72.08078	1068.61609	534.81168	1096.61100	548.80914	V	246.18123	123.59425	2
13						K	147.11281	74.06004	1

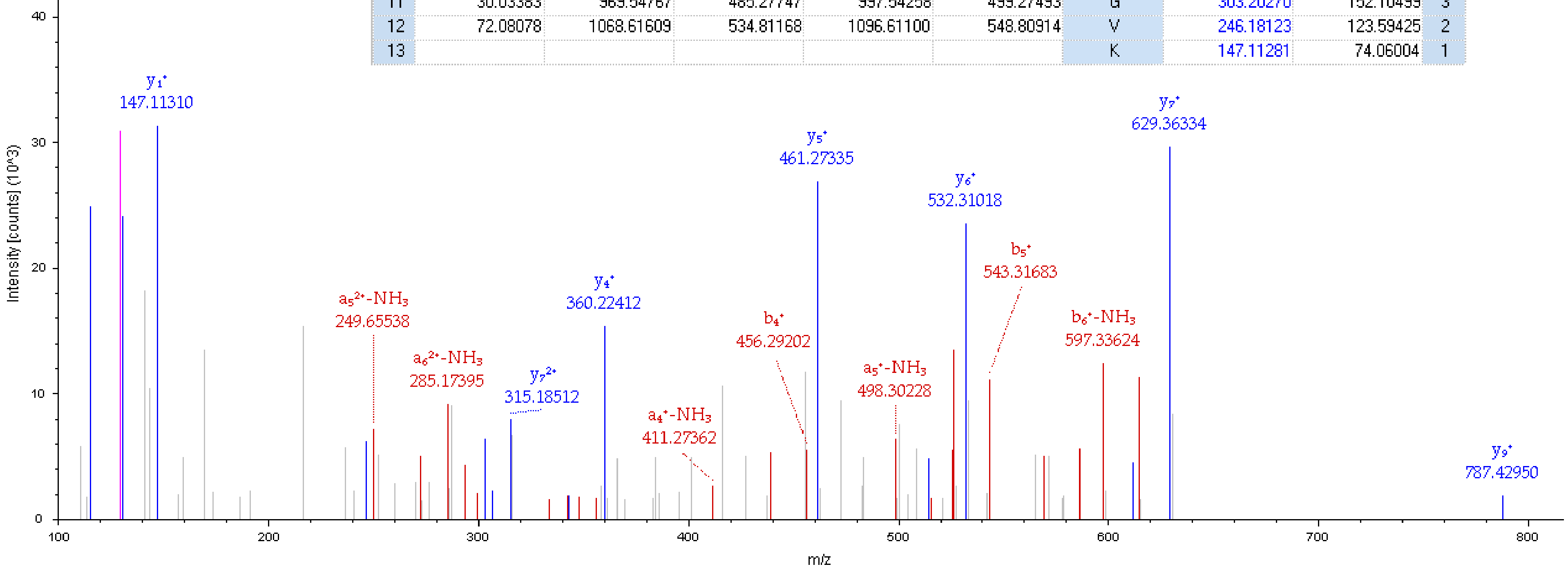

AUP sample #112

Accession	Description	Sequence	Modifications
Q16695	Histone H3.1t - [H31T_HUMAN]	VArKSAPATGGVK	R3(Deamidated)

Histone H3.1t peptide identified with the algorithm Andromeda using the MaxQuant software tool

Raw file	Scan	Method	Score	m/z	Gene names
1DLC022015QE_NET_#112_S1_rep3	3498	FTMS; HCD	66.99	414.91	HIST3H3

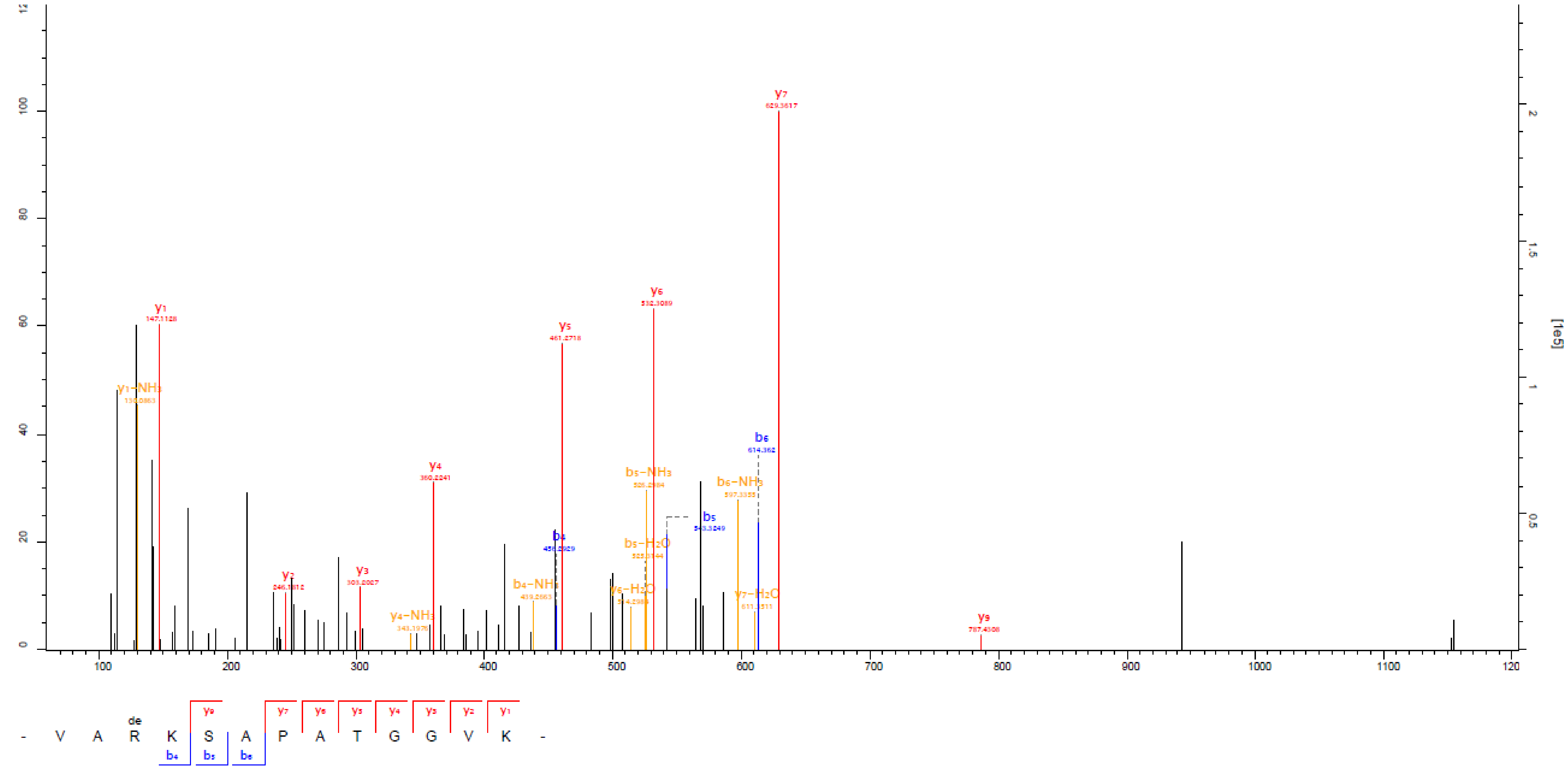

AUP sample #112

Sequence: ERSGVSLAALK, R2-Deamidated (0.98402 Da)
Charge: +2, Monoisotopic m/z: 566.32544 Da (+3.37 mmu/+5.94 ppm), MH+: 1131.64360 Da, RT: 65.30 min,
Identified with: Sequest HT (v1.3); XCorr:2.51, Ions matched by search engine: 0/0
Fragment match tolerance used for search: 0.02 Da
Fragments used for search: b; b-H_2O; b-NH_3; y; y-H_2O; y-NH_3

Protein references (3):
- Histone H1.3 OS=Homo sapiens GN=HIST1H1D PE=1 SV=2 - [H13_HUMAN]
- Histone H1.4 OS=Homo sapiens GN=HIST1H1E PE=1 SV=2 - [H14_HUMAN]
- Histone H1.2 OS=Homo sapiens GN=HIST1H1C PE=1 SV=2 - [H12_HUMAN]

#1	Immonium	a^+	a^{2+}	b^+	b^{2+}	Seq.	y^+	y^{2+}	#2
1	102.05496	102.05496	51.53112	130.04988	65.52858	E			11
2	129.11348	259.14010	130.07369	287.13501	144.07114	R-Deamid...	1002.59428	501.80078	10
3	60.04439	346.17213	173.58970	374.16704	187.58716	S	845.50914	423.25821	9
4	30.03383	403.19360	202.10044	431.18851	216.09789	G	758.47711	379.74219	8
5	72.08078	502.26202	251.63465	530.25693	265.63210	V	701.45564	351.23146	7
6	60.04439	589.29405	295.15066	617.28896	309.14812	S	602.38722	301.69725	6
7	86.09643	702.37812	351.69270	730.37303	365.69015	L	515.35519	258.18123	5
8	44.04948	773.41524	387.21126	801.41015	401.20871	A	402.27112	201.63920	4
9	44.04948	844.45236	422.72982	872.44727	436.72727	A	331.23400	166.12064	3
10	86.09643	957.53643	479.27185	985.53134	493.26931	L	260.19688	130.60208	2
11						K	147.11281	74.06004	1

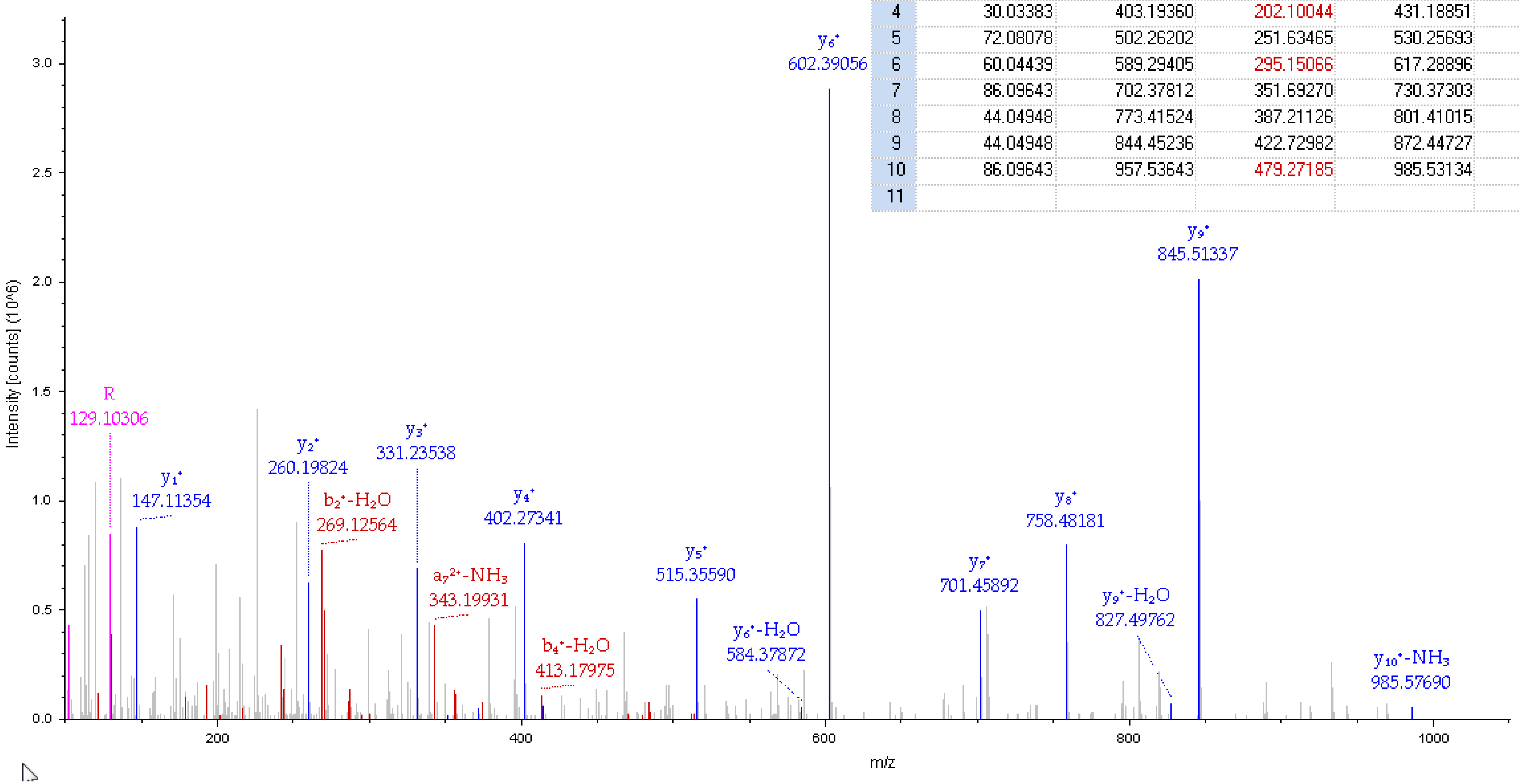

AUP sample #112

Accession	Description
P16401	Histone H1.5 -[H15_HUMAN]

Sequence	Modifications	XCorr	Charge	MH+ [Da]	ΔM [ppm]	# Missed Cleavages
ErNGLSLAALK	R2(Deamidated)	2.80	2	1172.6653	1.62	1

#1	b^+	b^{2+}	Seq.	y^+	y^{2+}	#2
1	130.04988	65.52858	E			11
2	287.13501	144.07114	R-Deamid...	1043.62083	522.31405	10
3	401.17794	201.09261	N	886.53569	443.77148	9
4	458.19941	229.60334	G	772.49276	386.75002	8
5	571.28348	286.14538	L	715.47129	358.23928	7
6	658.31551	329.66139	S	602.38722	301.69725	6
7	771.39958	386.20343	L	515.35519	258.18123	5
8	842.43670	421.72199	A	402.27112	201.63920	4
9	913.47382	457.24055	A	331.23400	166.12064	3
10	1026.55789	513.78258	L	260.19688	130.60208	2
11			K	147.11281	74.06004	1

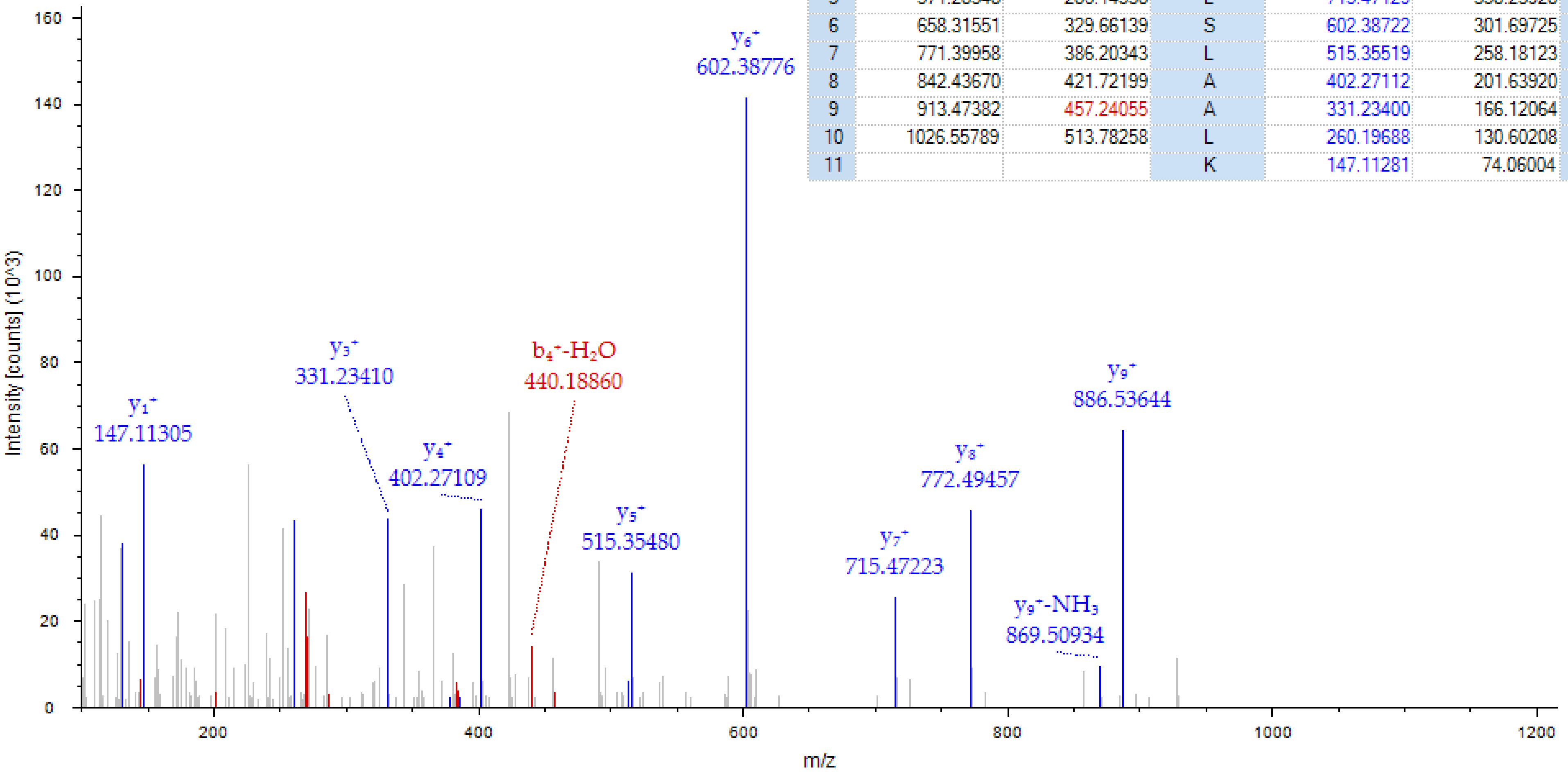

AUP sample #112

Accession	Description
P16401	Histone H1.5 - [H15_HUMAN]

Sequence	Modifications	XCorr	Charge	MH+ [Da]	ΔM [ppm]	# Missed Cleavages
ErnGLSLAALK	R2(Deamidated); N3(Deamidated)	2.56	2	1173.6504	2.55	1

#1	b^+	b^{2+}	Seq.	y^+	y^{2+}	#2
1	130.04988	65.52858	E			11
2	287.13501	144.07114	R-Deamid...	1044.60484	522.80606	10
3	402.16196	201.58462	N-Deamid...	887.51971	444.26349	9
4	459.18343	230.09535	G	772.49276	386.75002	8
5	572.26750	286.63739	L	715.47129	358.23928	7
6	659.29953	330.15340	S	602.38722	301.69725	6
7	772.38360	386.69544	L	515.35519	258.18123	5
8	843.42072	422.21400	A	402.27112	201.63920	4
9	914.45784	457.73256	A	331.23400	166.12064	3
10	1027.54191	514.27459	L	260.19688	130.60208	2
11			K	147.11281	74.06004	1

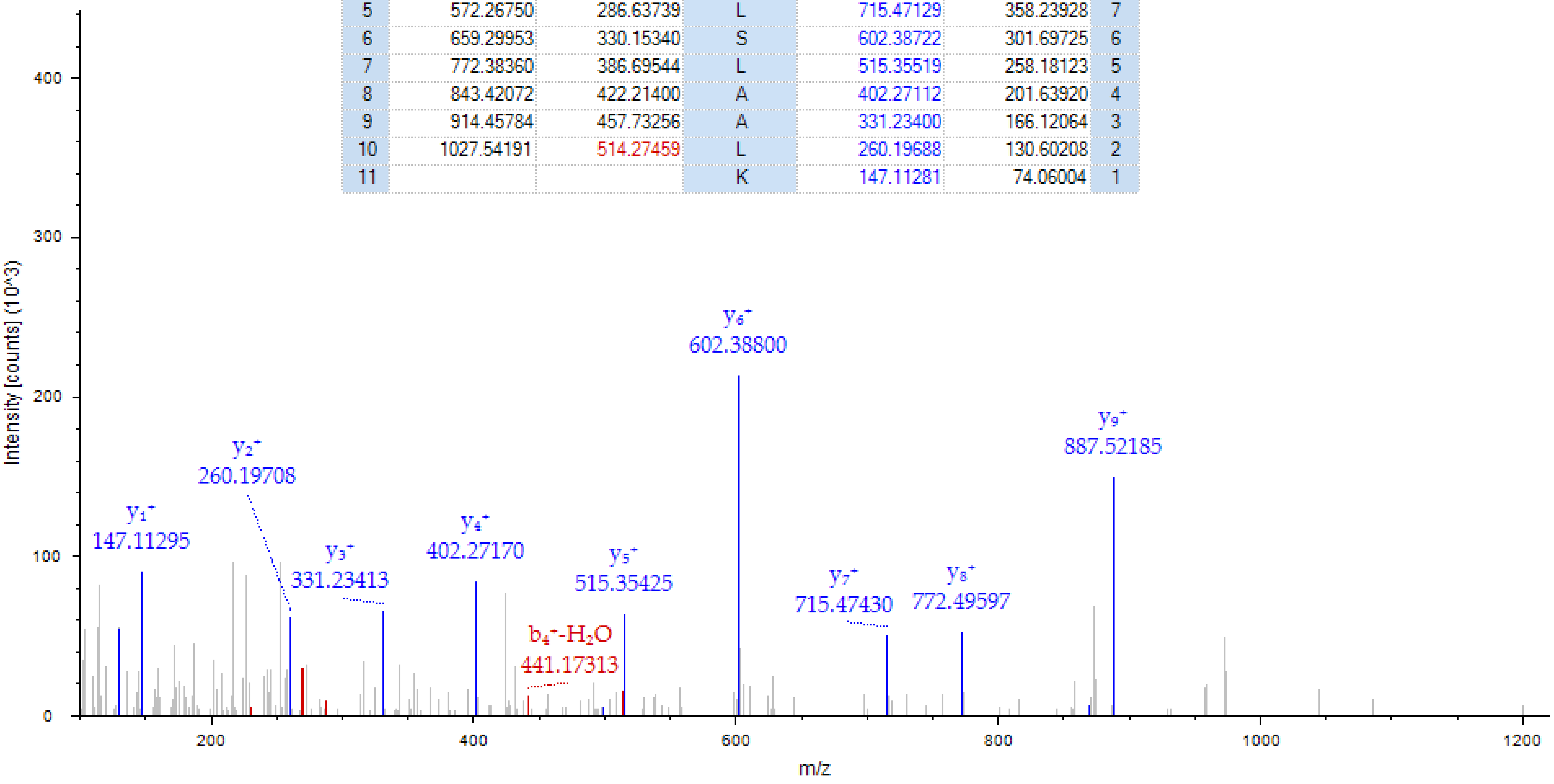

AUP sample #112

Histone H1.5 peptide identified with the algorithm Andromeda using the MaxQuant software tool

Accession	Description
P16401	Histone H1.5 - [H15_HUMAN]

Sequence	Modifications
ErNGLSLAALK	R2(Deamidated)

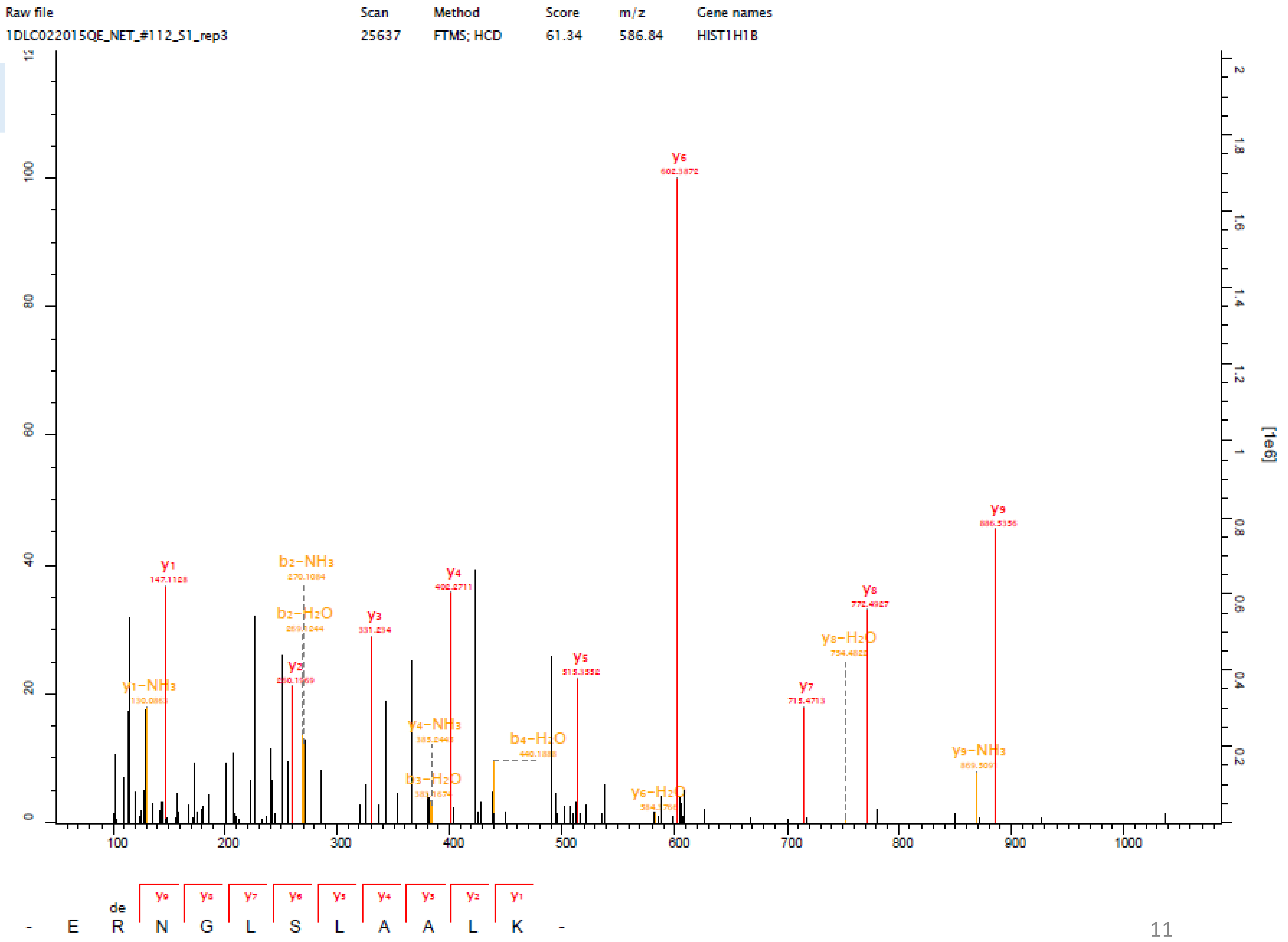

AUP sample #112

Accession	Description
P16401	Histone H1.5 - [H15_HUMAN]

Sequence	Modifications	XCorr	Charge	MH+ [Da]	ΔM [ppm]	# Missed Cleavages
ErNGLSLAALK	R2(Deamidated)	2.80	2	1172.6653	1.62	1

#1	b^+	b^{2+}	Seq.	y^+	y^{2+}	#2
1	130.04988	65.52858	E			11
2	287.13501	144.07114	R-Deamid...	1043.62083	522.31405	10
3	401.17794	201.09261	N	886.53569	443.77148	9
4	458.19941	229.60334	G	772.49276	386.75002	8
5	571.28348	286.14538	L	715.47129	358.23928	7
6	658.31551	329.66139	S	602.38722	301.69725	6
7	771.39958	386.20343	L	515.35519	258.18123	5
8	842.43670	421.72199	A	402.27112	201.63920	4
9	913.47382	457.24055	A	331.23400	166.12064	3
10	1026.55789	513.78258	L	260.19688	130.60208	2
11			K	147.11281	74.06004	1

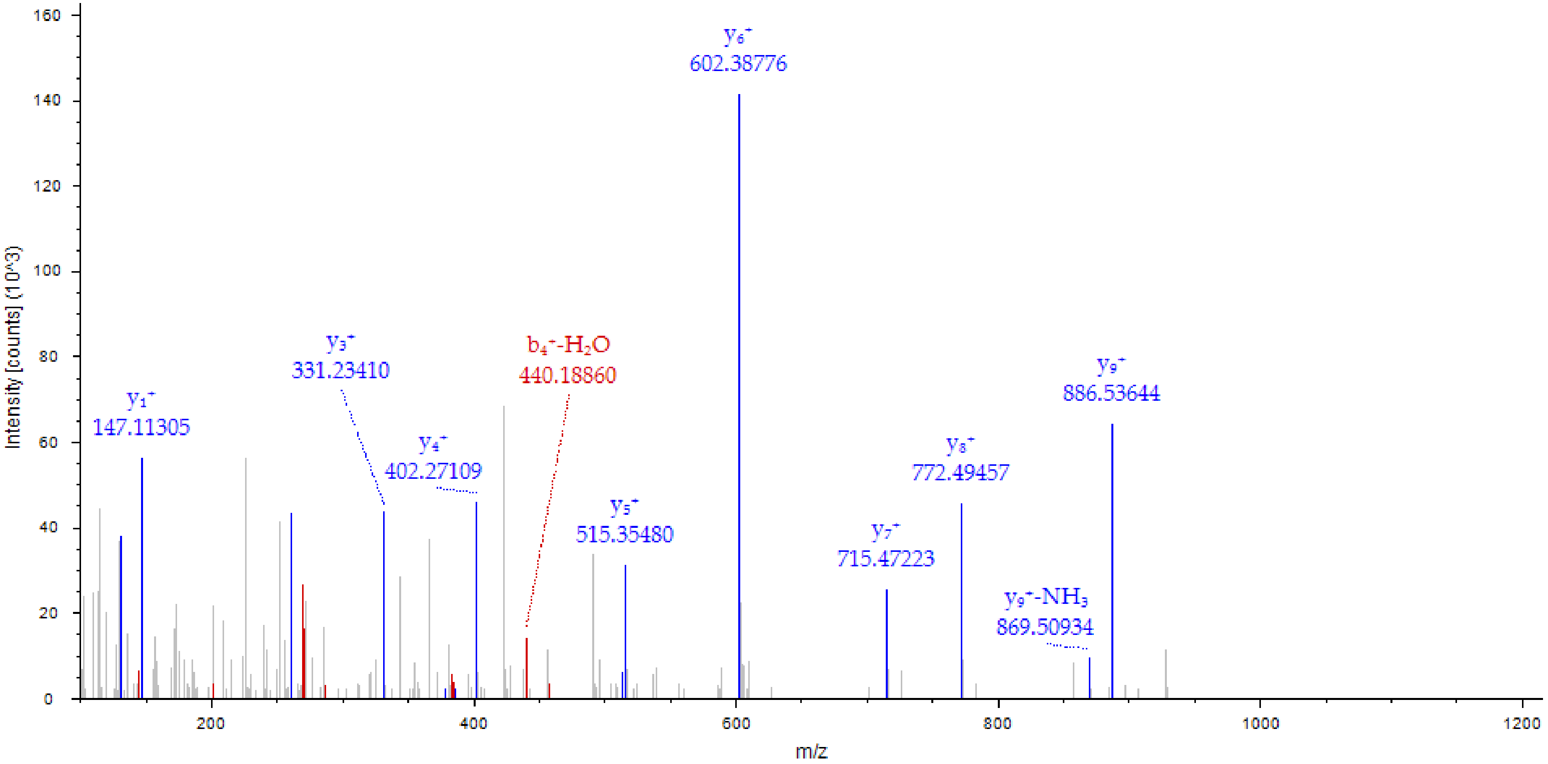

AUP sample #112

Sequence: GYSFTTTAE**R(206)**EIVR, R10-Deamidated (0.98402 Da)
Charge: +2, Monoisotopic m/z: 815.90991 Da (+2.68 mmu/+3.29 ppm), MH+: 1630.81255 Da, RT: 87.03 min,
Identified with: Sequest HT (v1.3); XCorr:2.79, Ions matched by search engine: 0/0
Fragment match tolerance used for search: 0.02 Da
Fragments used for search: b; b-H_2O; b-NH_3; y; y-H_2O; y-NH_3

Protein references (1):
- Actin, cytoplasmic 1 OS=Homo sapiens GN=ACTB PE=1 SV=1 - [ACTB_HUMAN]

neutral loss peptide

#1	Immonium	a^+	a^{2+}	b^+	b^{2+}	Seq.	y^+	y^{2+}	#2
1	30.03383	30.03383	15.52055	58.02875	29.51801	G			14
2	136.07568	193.09715	97.05221	221.09207	111.04967	Y	1573.78572	787.39650	13
3	60.04439	280.12918	140.56823	308.12410	154.56569	S	1410.72240	705.86484	12
4	120.08078	427.19760	214.10244	455.19252	228.09990	F	1323.69037	662.34882	11
5	74.06004	528.24528	264.62628	556.24020	278.62374	T	1176.62195	588.81461	10
6	74.06004	629.29296	315.15012	657.28788	329.14758	T	1075.57427	538.29077	9
7	74.06004	730.34064	365.67396	758.33556	379.67142	T	974.52659	487.76693	8
8	44.04948	801.37776	401.19252	829.37268	415.18998	A	873.47891	437.24309	7
9	102.05496	930.42036	465.71382	958.41528	479.71128	E	802.44179	401.72453	6
10	129.11348	1087.50550	544.25639	1115.50041	558.25384	R-Deamid...	673.39919	337.20323	5
11	102.05496	1216.54810	608.77769	1244.54301	622.77514	E	516.31405	258.66066	4
12	86.09643	1329.63217	665.31972	1357.62708	679.31718	I	387.27145	194.13936	3
13	72.08078	1428.70059	714.85393	1456.69550	728.85139	V	274.18738	137.59733	2
14						R	175.11896	88.06312	1

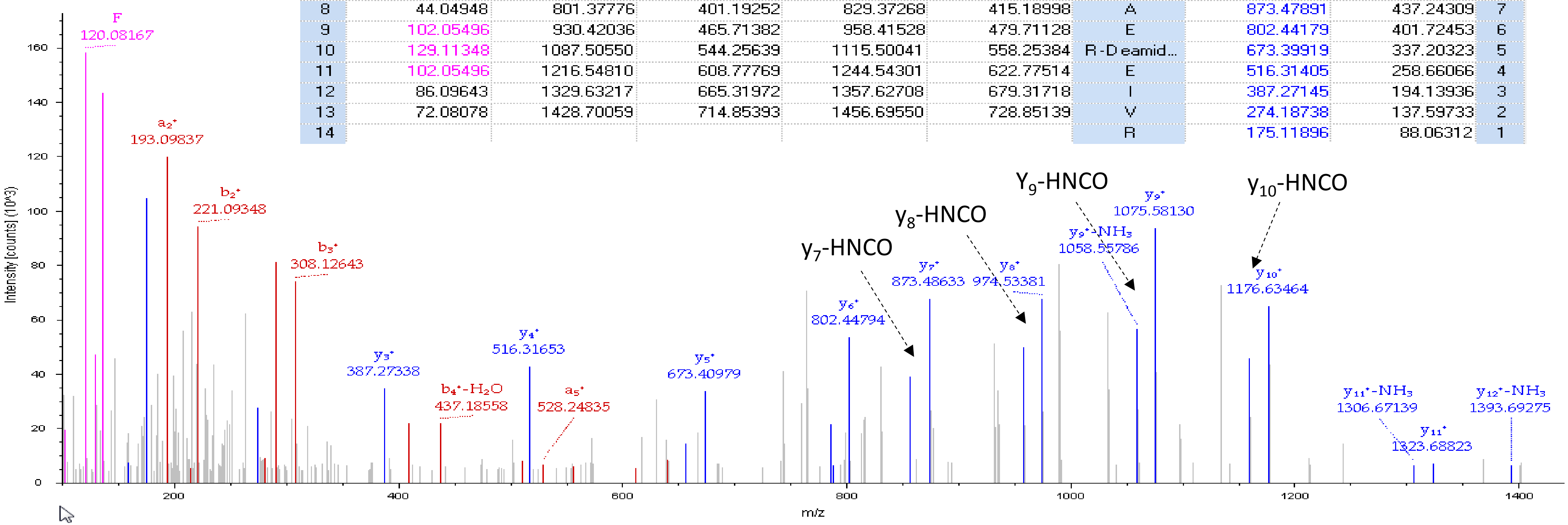

AUP sample #112

Sequence: **R(62)**GILTLK, R1-Deamidated (0.98402 Da)
Charge: +2, Monoisotopic m/z: 401.26373 Da (+0.44 mmu/+1.1 ppm), MH+: 801.52019 Da, RT: 59.34 min,
Identified with: Sequest HT (v1.3); XCorr:1.83, Ions matched by search engine: 0/0
Fragment match tolerance used for search: 0.02 Da
Fragments used for search: b; b-H_2O; b-NH_3; y; y-H_2O; y-NH_3

Protein references (1):
- Actin, cytoplasmic 1 OS=Homo sapiens GN=ACTB PE=1 SV=1 - [ACTB_HUMAN]]

#1	Immonium	a⁺	a²⁺	b⁺	b²⁺	Seq.	y⁺	y²⁺	#2
1	129.11348	130.09750	65.55239	158.09241	79.54984	R-Deamid...			7
2	30.03383	187.11897	94.06312	215.11388	108.06058	G	644.43417	322.72072	6
3	86.09643	300.20304	150.60516	328.19795	164.60261	I	587.41270	294.20999	5
4	86.09643	413.28711	207.14719	441.28202	221.14465	L	474.32863	237.66795	4
5	74.06004	514.33479	257.67103	542.32970	271.66849	T	361.24456	181.12592	3
6	86.09643	627.41886	314.21307	655.41377	328.21052	L	260.19688	130.60208	2
7						K	147.11281	74.06004	1

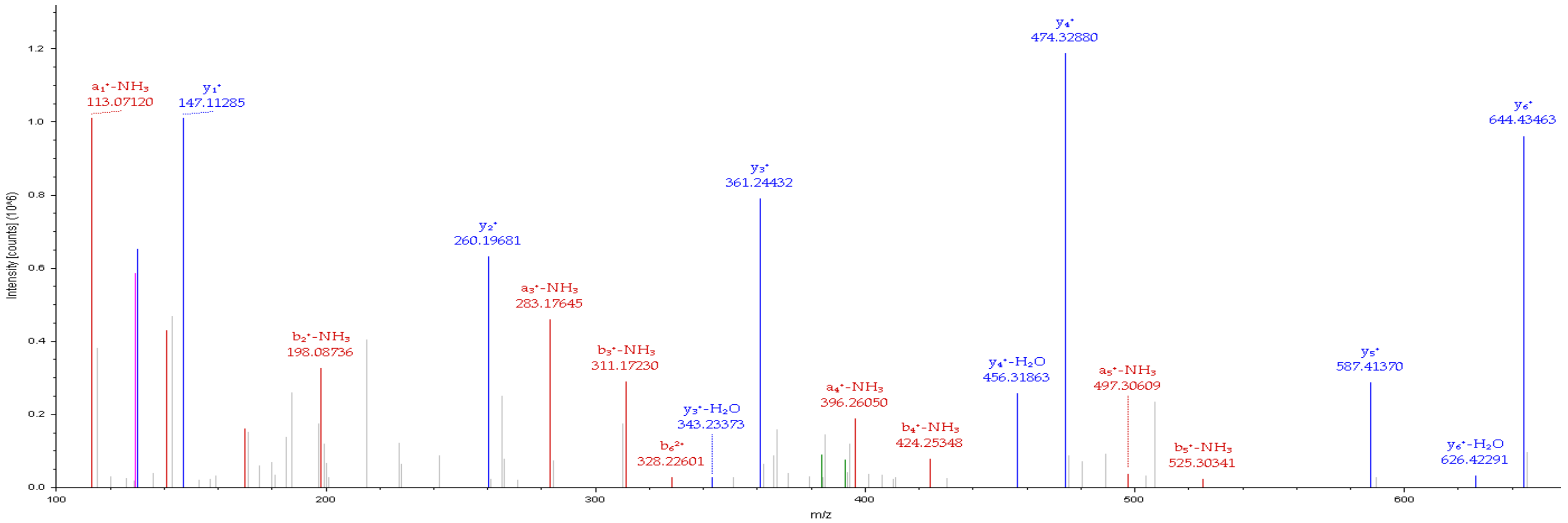

AUP sample #112

Sequence: RAAPEASGTPSSDAVSR, R1-Deamidated (0.98402 Da)
Charge: +2, Monoisotopic m/z: 830.40466 Da (+4.35 mmu/+5.24 ppm), MH+: 1659.80205 Da, RT: 41.06 min,
Identified with: Sequest HT (v1.3); XCorr:3.05, Ions matched by search engine: 0/0
Fragment match tolerance used for search: 0.02 Da
Fragments used for search: b; b-H_2O; b-NH_3; y; y-H_2O; y-NH_3

Protein references (1):
- Coronin-1A OS=Homo sapiens GN=CORO1A PE=1 SV=4 - [COR1A_HUMAN]

#1	Immonium	a⁺	a²⁺	b⁺	b²⁺	Seq.	y⁺	y²⁺	#2
1	129.11348	130.09750	65.55239	158.09241	79.54984	R-Deamid...			17
2	44.04948	201.13462	101.07095	229.12953	115.06840	A	1502.70822	751.85775	16
3	44.04948	272.17174	136.58951	300.16665	150.58696	A	1431.67110	716.33919	15
4	70.06513	369.22451	185.11589	397.21942	199.11335	P	1360.63398	680.82063	14
5	102.05496	498.26711	249.63719	526.26202	263.63465	E	1263.58121	632.29424	13
6	44.04948	569.30423	285.15575	597.29914	299.15321	A	1134.53861	567.77294	12
7	60.04439	656.33626	328.67177	684.33117	342.66922	S	1063.50149	532.25438	11
8	30.03383	713.35773	357.18250	741.35264	371.17996	G	976.46946	488.73837	10
9	74.06004	814.40541	407.70634	842.40032	421.70380	T	919.44799	460.22763	9
10	70.06513	911.45818	456.23273	939.45309	470.23018	P	818.40031	409.70379	8
11	60.04439	998.49021	499.74874	1026.48512	513.74620	S	721.34754	361.17741	7
12	60.04439	1085.52224	543.26476	1113.51715	557.26221	S	634.31551	317.66139	6
13	88.03931	1200.54919	600.77823	1228.54410	614.77569	D	547.28348	274.14538	5
14	44.04948	1271.58631	636.29679	1299.58122	650.29425	A	432.25653	216.63190	4
15	72.08078	1370.65473	685.83100	1398.64964	699.82846	V	361.21941	181.11334	3
16	60.04439	1457.68676	729.34702	1485.68167	743.34447	S	262.15099	131.57913	2
17						R	175.11896	88.06312	1

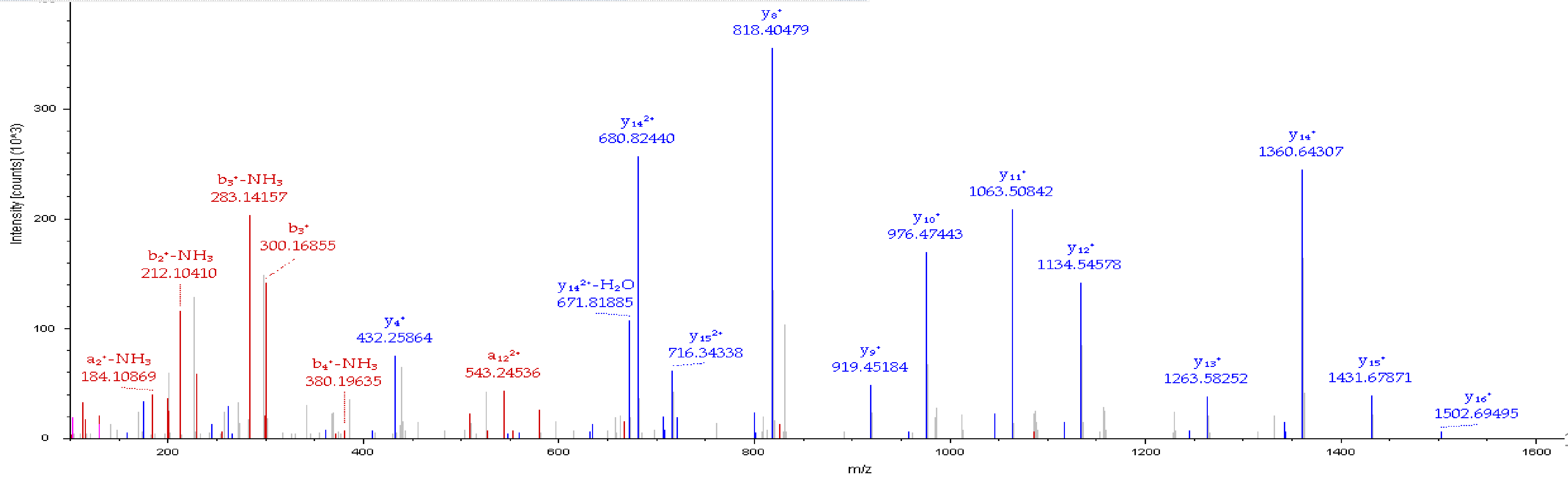

AUP sample #112

Sequence: SRLGDLYEEEMR, R2-Deamidated (0.98402 Da)
Charge: +2, Monoisotopic m/z: 749.85101 Da (+5.22 mmu/+6.97 ppm), MH+: 1498.69475 Da, RT: 85.96 min,
Identified with: Sequest HT (v1.3); XCorr:2.52, Ions matched by search engine: 0/0
Fragment match tolerance used for search: 0.02 Da
Fragments used for search: b; b-H_2O; b-NH_3; y; y-H_2O; y-NH_3

Protein references (1):
- Vimentin OS=Homo sapiens GN=VIM PE=1 SV=4 - [VIME_HUMAN]

#1	Immonium	a^+	b^+	Seq.	y^+	#2
1	60.04439	60.04439	88.03931	S		12
2	129.11348	217.12953	245.12444	R-Deamid...	1411.65228	11
3	86.09643	330.21360	358.20851	L	1254.56714	10
4	30.03383	387.23507	415.22998	G	1141.48307	9
5	88.03931	502.26202	530.25693	D	1084.46160	8
6	86.09643	615.34609	643.34100	L	969.43465	7
7	136.07568	778.40941	806.40432	Y	856.35058	6
8	102.05496	907.45201	935.44692	E	693.28726	5
9	102.05496	1036.49461	1064.48952	E	564.24466	4
10	102.05496	1165.53721	1193.53212	E	435.20206	3
11	104.05286	1296.57771	1324.57262	M	306.15946	2
12				R	175.11896	1

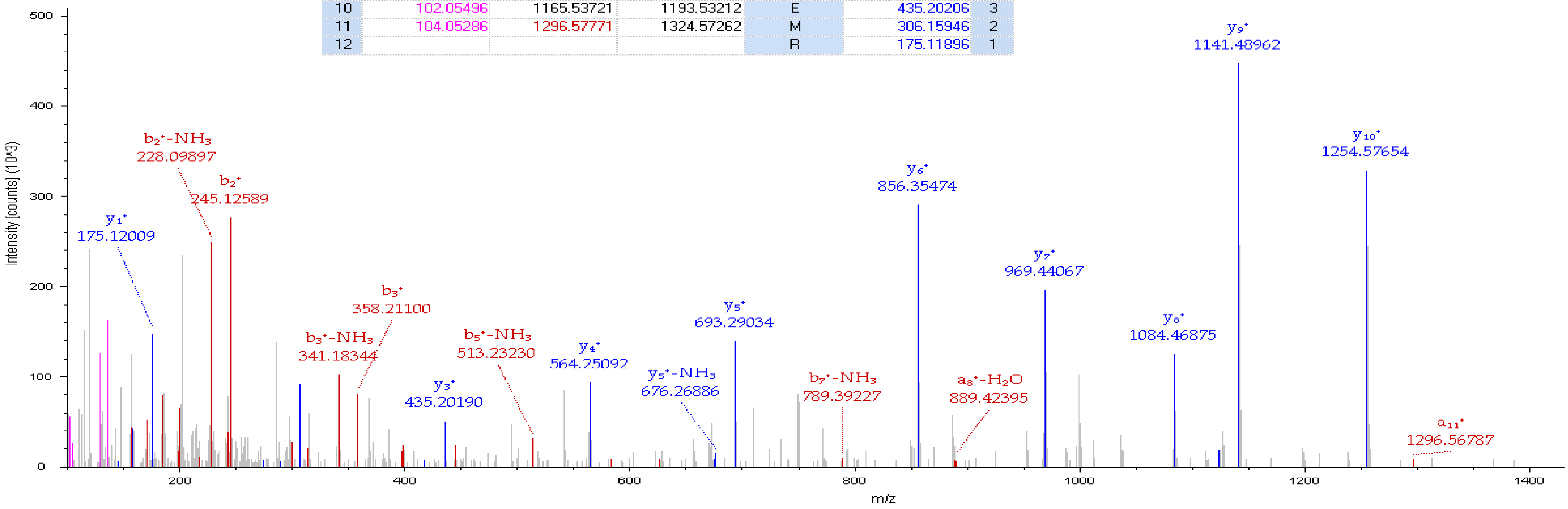

AUP sample #112

Sequence: MGSRAGGPTTPLSPTR, M1-Oxidation (15.99492 Da), R4-Deamidated (0.98402 Da)
Charge: +2, Monoisotopic m/z: 801.89972 Da (+0.82 mmu/+1.02 ppm), MH+: 1602.79216 Da,
RT: 34.79 min,
Identified with: Sequest HT (v1.3); XCorr:2.99, Ions matched by search engine: 0/0
Fragment match tolerance used for search: 0.02 Da
Fragments used for search: b; b-H_2O; b-NH_3; y; y-H_2O; y-NH_3

Protein references (1):
- Lamin-B1 OS=Homo sapiens GN=LMNB1 PE=1 SV=2 - [LMNB1_HUMAN]

#1	Immonium	a^+	b^+	Seq.	y^+	#2
1	104.05286	120.04778	148.04269	M-Oxidation		16
2	30.03383	177.06925	205.06416	G	1455.75511	15
3	60.04439	264.10128	292.09619	S	1398.73364	14
4	129.11348	421.18641	449.18133	R-Deamid...	1311.70161	13
5	44.04948	492.22353	520.21845	A	1154.61647	12
6	30.03383	549.24500	577.23992	G	1083.57935	11
7	30.03383	606.26647	634.26139	G	1026.55788	10
8	70.06513	703.31924	731.31416	P	969.53641	9
9	74.06004	804.36692	832.36184	T	872.48364	8
10	74.06004	905.41460	933.40952	T	771.43596	7
11	70.06513	1002.46737	1030.46229	P	670.38828	6
12	86.09643	1115.55144	1143.54636	L	573.33551	5
13	60.04439	1202.58347	1230.57839	S	460.25144	4
14	70.06513	1299.63624	1327.63116	P	373.21941	3
15	74.06004	1400.68392	1428.67884	T	276.16664	2
16				R	175.11896	1

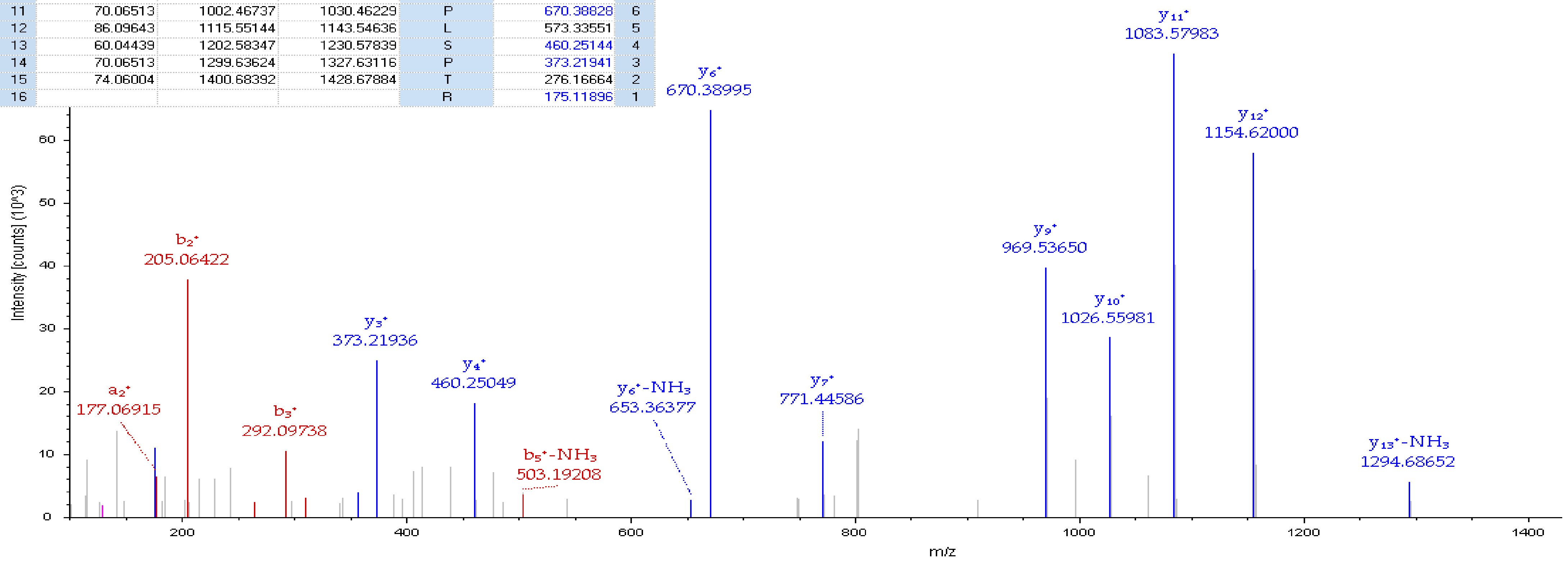

AUP sample #112

Sequence: LLIYGASTRATGIPAR, R9-Deamidated (0.98402 Da), R16-Deamidated (0.98402 Da)
Charge: +2, Monoisotopic m/z: 831.46680 Da (+2.07 mmu/+2.49 ppm), MH+: 1661.92632 Da, RT: 52.10 min,
Identified with: Sequest HT (v1.3); XCorr:2.80, Ions matched by search engine: 0/0
Fragment match tolerance used for search: 0.02 Da
Fragments used for search: b; b-H_2O; b-NH_3; y; y-H_2O; y-NH_3

Protein references (1):
- Ig kappa chain V-III region POM OS=Homo sapiens PE=1 SV=1 - [KV306_HUMAN]

#1	Immonium	a^+	a^{2+}	b^+	b^{2+}	Seq.	y^+	y^{2+}	#2
1	86.09643	86.09643	43.55185	114.09135	57.54931	L			16
2	86.09643	199.18050	100.09389	227.17542	114.09135	L	1548.83810	774.92269	15
3	86.09643	312.26457	156.63592	340.25949	170.63338	I	1435.75403	718.38065	14
4	136.07568	475.32789	238.16758	503.32281	252.16504	Y	1322.66996	661.83862	13
5	30.03383	532.34936	266.67832	560.34428	280.67578	G	1159.60664	580.30696	12
6	44.04948	603.38648	302.19688	631.38140	316.19434	A	1102.58517	551.79622	11
7	60.04439	690.41851	345.71289	718.41343	359.71035	S	1031.54805	516.27766	10
8	74.06004	791.46619	396.23673	819.46111	410.23419	T	944.51602	472.76165	9
9	129.11348	948.55133	474.77930	976.54624	488.77676	R-Deamid...	843.46834	422.23781	8
10	44.04948	1019.58845	510.29786	1047.58336	524.29532	A	686.38321	343.69524	7
11	74.06004	1120.63613	560.82170	1148.63104	574.81916	T	615.34609	308.17668	6
12	30.03383	1177.65760	589.33244	1205.65251	603.32989	G	514.29841	257.65284	5
13	86.09643	1290.74167	645.87447	1318.73658	659.87193	I	457.27694	229.14211	4
14	70.06513	1387.79444	694.40086	1415.78935	708.39831	P	344.19287	172.60007	3
15	44.04948	1458.83156	729.91942	1486.82647	743.91687	A	247.14010	124.07369	2
16						R-Deamid...	176.10298	88.55513	1

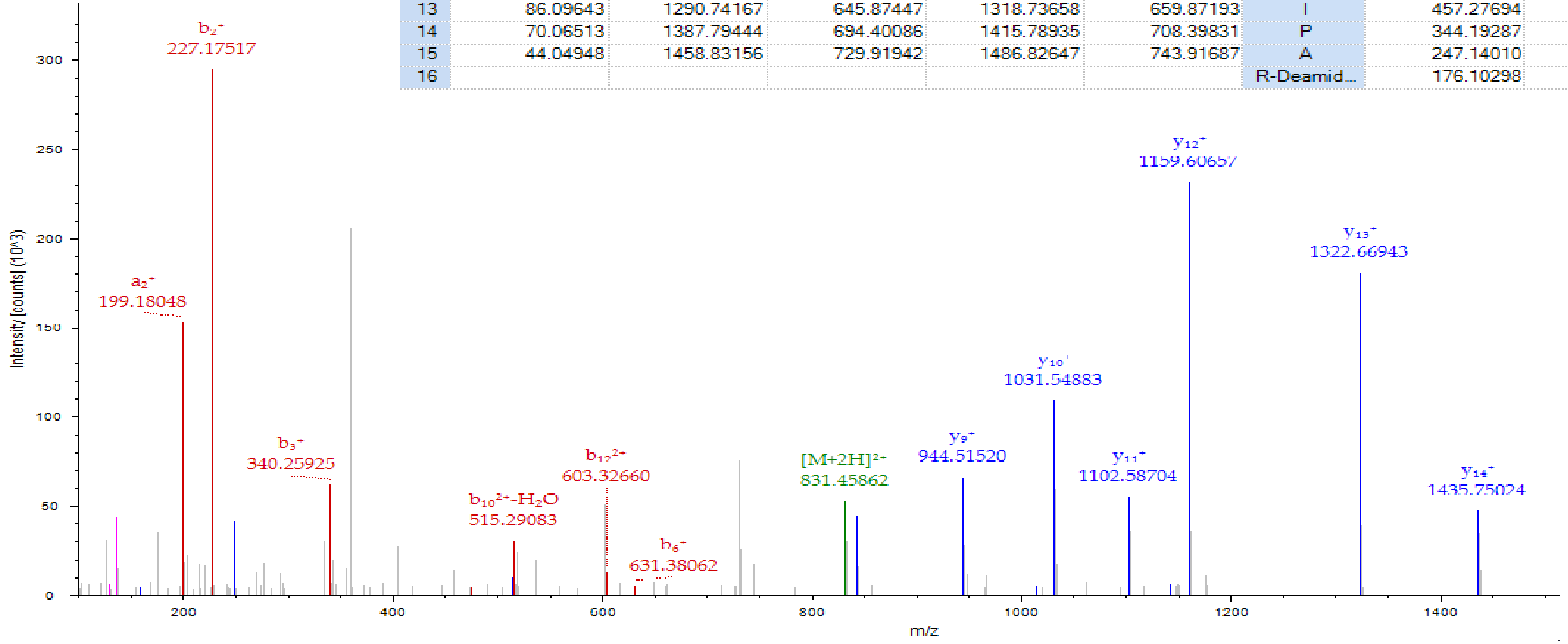

AUP sample #112

Sequence: SPEDLERLLPHK, R7-Deamidated (0.98402 Da)
Charge: +3, Monoisotopic m/z: 478.92767 Da (+3.23 mmu/+6.75 ppm), MH+: 1434.76847 Da, RT: 63.36 min,
Identified with: Sequest HT (v1.3); XCorr:2.54, Ions matched by search engine: 0/0
Fragment match tolerance used for search: 0.02 Da
Fragments used for search: b; b-H_2O; b-NH_3; y; y-H_2O; y-NH_3

Protein references (1):
- Glucose-6-phosphate isomerase OS=Homo sapiens GN=GPI PE=1 SV=4 - [G6PI_HUMAN]

#1	Immonium	a^+	a^{2+}	b^+	b^{2+}	Seq.	y^+	y^{2+}	#2
1	60.04439	60.04439	30.52583	88.03931	44.52329	S			12
2	70.06513	157.09716	79.05222	185.09208	93.04968	P	1347.72676	674.36702	11
3	102.05496	286.13976	143.57352	314.13468	157.57098	E	1250.67399	625.84063	10
4	88.03931	401.16671	201.08699	429.16163	215.08445	D	1121.63139	561.31933	9
5	86.09643	514.25078	257.62903	542.24570	271.62649	L	1006.60444	503.80586	8
6	102.05496	643.29338	322.15033	671.28830	336.14779	E	893.52037	447.26382	7
7	129.11348	800.37852	400.69290	828.37343	414.69035	R-Deamid...	764.47777	382.74252	6
8	86.09643	913.46259	457.23493	941.45750	471.23239	L	607.39263	304.19995	5
9	86.09643	1026.54666	513.77697	1054.54157	527.77442	L	494.30856	247.65792	4
10	70.06513	1123.59943	562.30335	1151.59434	576.30081	P	381.22449	191.11588	3
11	110.07127	1260.65834	630.83281	1288.65325	644.83026	H	284.17172	142.58950	2
12						K	147.11281	74.06004	1

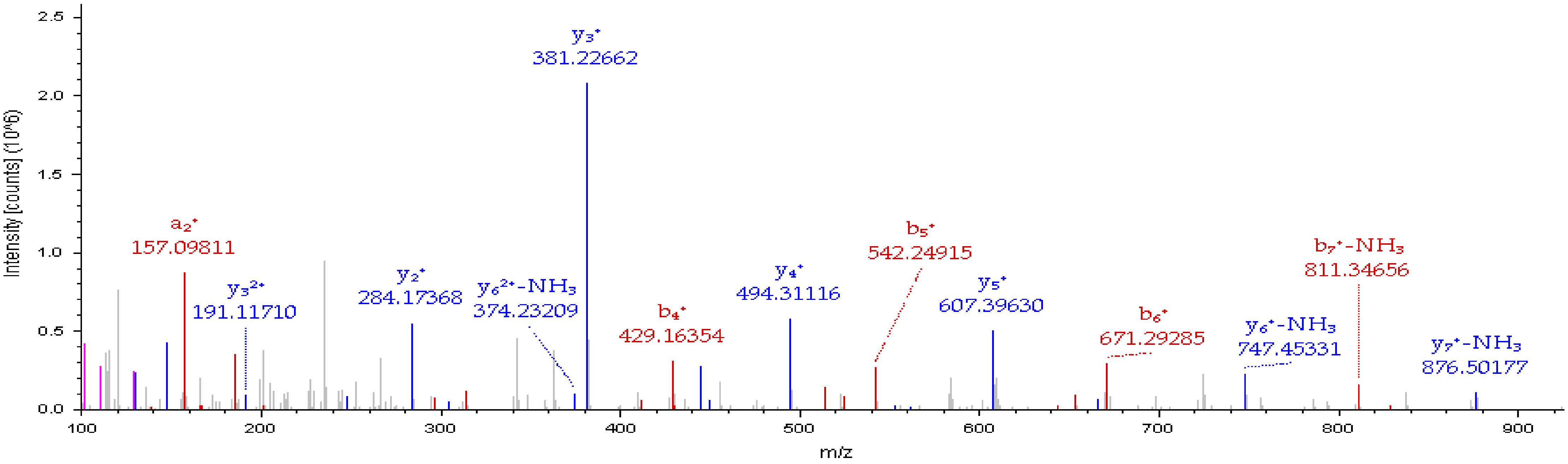

AUP sample #134

Sequence: MSQLERNIETIINTFHQYSVK, R6-Deamidated (0.98402 Da)
Charge: +3, Monoisotopic m/z: 851.43512 Da (+3.13 mmu/+3.67 ppm), MH+: 2552.29081 Da, RT: 104.83 min,
Identified with: Sequest HT (v1.3); XCorr:3.63, Ions matched by search engine: 0/0
Fragment match tolerance used for search: 0.02 Da
Fragments used for search: b; b-H_2O; b-NH_3; y; y-H_2O; y-NH_3

Protein references (1):
- Protein S100-A9 OS=Homo sapiens GN=S100A9 PE=1 SV=1 - [S10A9_HUMAN]

#1	Immonium	a^+	a^{2+}	b^+	b^{2+}	Seq.	y^+	y^{2+}	#2
1	104.05286	104.05286	52.53007	132.04778	66.52753	M			21
2	60.04439	191.08489	96.04608	219.07981	110.04354	S	2421.24094	1211.12411	20
3	101.07094	319.14347	160.07537	347.13839	174.07283	Q	2334.20891	1167.60809	19
4	86.09643	432.22754	216.61741	460.22246	230.61487	L	2206.15033	1103.57880	18
5	102.05496	561.27014	281.13871	589.26506	295.13617	E	2093.06626	1047.03677	17
6	129.11348	718.35528	359.68128	746.35019	373.67873	R-Deamid...	1964.02366	982.51547	16
7	87.05529	832.39821	416.70274	860.39312	430.70020	N	1806.93852	903.97290	15
8	86.09643	945.48228	473.24478	973.47719	487.24223	I	1692.89559	846.95143	14
9	102.05496	1074.52488	537.76608	1102.51979	551.76353	E	1579.81152	790.40940	13
10	74.06004	1175.57256	588.28992	1203.56747	602.28737	T	1450.76892	725.88810	12
11	86.09643	1288.65663	644.83195	1316.65154	658.82941	I	1349.72124	675.36426	11
12	86.09643	1401.74070	701.37399	1429.73561	715.37144	I	1236.63717	618.82222	10
13	87.05529	1515.78363	758.39545	1543.77854	772.39291	N	1123.55310	562.28019	9
14	74.06004	1616.83131	808.91929	1644.82622	822.91675	T	1009.51017	505.25872	8
15	120.08078	1763.89973	882.45350	1791.89464	896.45096	F	908.46249	454.73488	7
16	110.07127	1900.95864	950.98296	1928.95355	964.98041	H	761.39407	381.20067	6
17	101.07094	2029.01722	1015.01225	2057.01213	1029.00970	Q	624.33516	312.67122	5
18	136.07568	2192.08054	1096.54391	2220.07545	1110.54136	Y	496.27658	248.64193	4
19	60.04439	2279.11257	1140.05992	2307.10748	1154.05738	S	333.21326	167.11027	3
20	72.08078	2378.18099	1189.59413	2406.17590	1203.59159	V	246.18123	123.59425	2
21						K	147.11281	74.06004	1

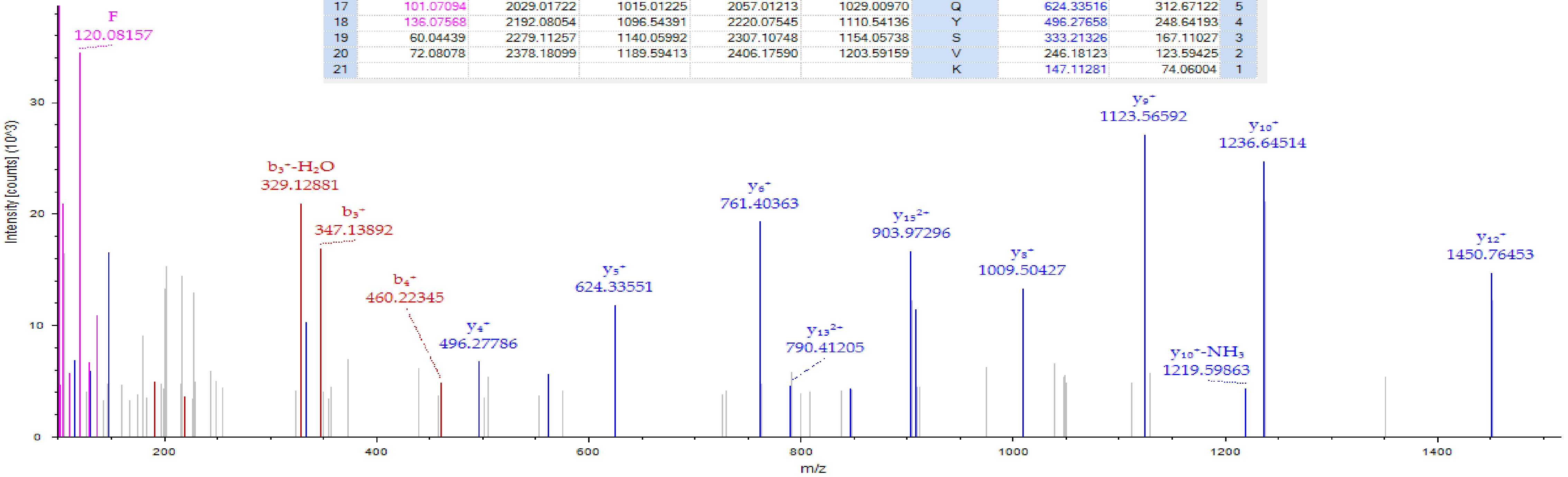

AUP sample #134

Sequence: VPFSLLRGPSWDPFR, R7-Deamidated (0.98402 Da)
Charge: +2, Monoisotopic m/z: 887.96918 Da (+1.74 mmu/+1.96 ppm), MH+: 1774.93108 Da, RT: 89.74 min,
Identified with: Sequest HT (v1.3); XCorr:2.47, Ions matched by search engine: 0/0
Fragment match tolerance used for search: 0.02 Da
Fragments used for search: b; b-H_2O; b-NH_3; y; y-H_2O; y-NH_3

Protein references (1):
- Heat shock protein beta-1 OS=Homo sapiens GN=HSPB1 PE=2 SV=1 - [F8WE04_HUMAN]

#1	Immonium	a^+	b^+	Seq.	y^+	#2
1	72.08078	72.08078	100.07570	V		15
2	70.06513	169.13355	197.12847	P	1675.85919	14
3	120.08078	316.20197	344.19689	F	1578.80642	13
4	60.04439	403.23400	431.22892	S	1431.73800	12
5	86.09643	516.31807	544.31299	L	1344.70597	11
6	86.09643	629.40214	657.39706	L	1231.62190	10
7	129.11348	786.48728	814.48219	R-Deamid...	1118.53783	9
8	30.03383	843.50875	871.50366	G	961.45269	8
9	70.06513	940.56152	968.55643	P	904.43122	7
10	60.04439	1027.59355	1055.58846	S	807.37845	6
11	159.09168	1213.67287	1241.66778	W	720.34642	5
12	88.03931	1328.69982	1356.69473	D	534.26710	4
13	70.06513	1425.75259	1453.74750	P	419.24015	3
14	120.08078	1572.82101	1600.81592	F	322.18738	2
15				R	175.11896	1

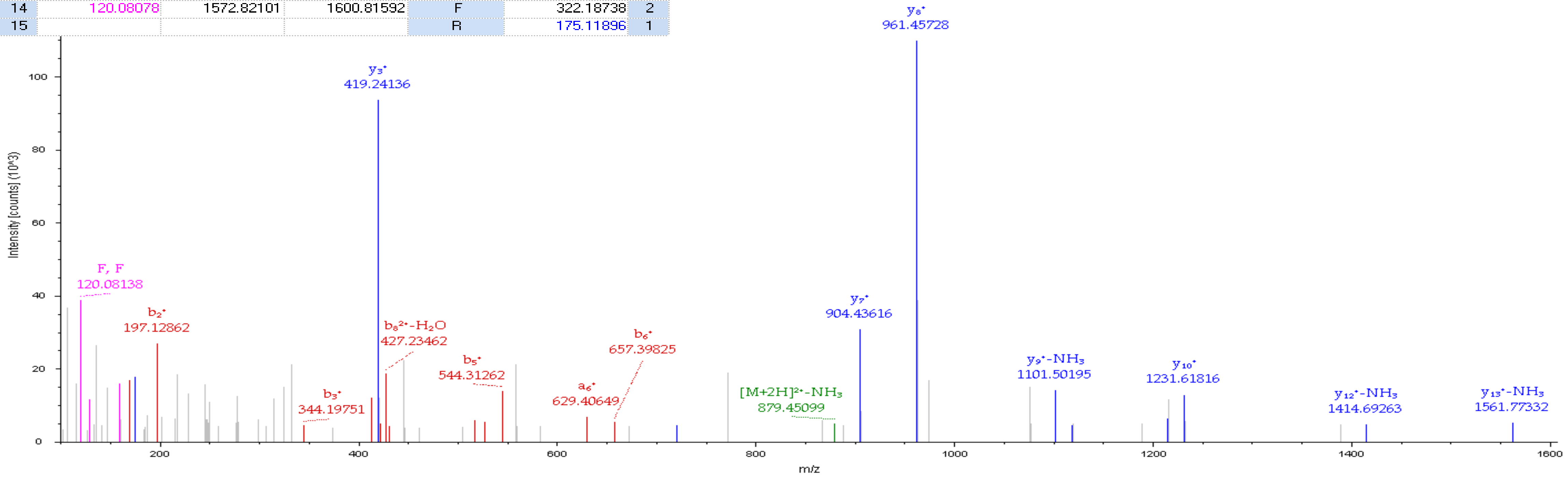

AUP sample #33

Sequence: LRPVAAEVYGTER, R2-Deamidated (0.98402 Da)
Charge: +2, Monoisotopic m/z: 731.39270 Da (+4.22 mmu/+5.77 ppm), MH+: 1461.77812 Da, RT: 27.99 min,
Identified with: Sequest HT (v1.3); XCorr:2.27, Ions matched by search engine: 0/0
Fragment match tolerance used for search: 0.02 Da
Fragments used for search: b; b-H_2O; b-NH_3; y; y-H_2O; y-NH_3

Protein references (1):
- Lactotransferrin OS=Homo sapiens GN=LTF PE=1 SV=6 - [TRFL_HUMAN]

#1	Immonium	a⁺	a²⁺	b⁺	b²⁺	Seq.	y⁺	y²⁺	#2
1	86.09643	86.09643	43.55185	114.09135	57.54931	L			13
2	129.11348	243.18157	122.09442	271.17648	136.09188	R-Deamid...	1348.68562	674.84645	12
3	70.06513	340.23434	170.62081	368.22925	184.61826	P	1191.60048	596.30388	11
4	72.08078	439.30276	220.15502	467.29767	234.15247	V	1094.54771	547.77749	10
5	44.04948	510.33988	255.67358	538.33479	269.67103	A	995.47929	498.24328	9
6	44.04948	581.37700	291.19214	609.37191	305.18959	A	924.44217	462.72472	8
7	102.05496	710.41960	355.71344	738.41451	369.71089	E	853.40505	427.20616	7
8	72.08078	809.48802	405.24765	837.48293	419.24510	V	724.36245	362.68486	6
9	136.07568	972.55134	486.77931	1000.54625	500.77676	Y	625.29403	313.15065	5
10	30.03383	1029.57281	515.29004	1057.56772	529.28750	G	462.23071	231.61899	4
11	74.06004	1130.62049	565.81388	1158.61540	579.81134	T	405.20924	203.10826	3
12	102.05496	1259.66309	630.33518	1287.65800	644.33264	E	304.16156	152.58442	2
13						R	175.11896	88.06312	1

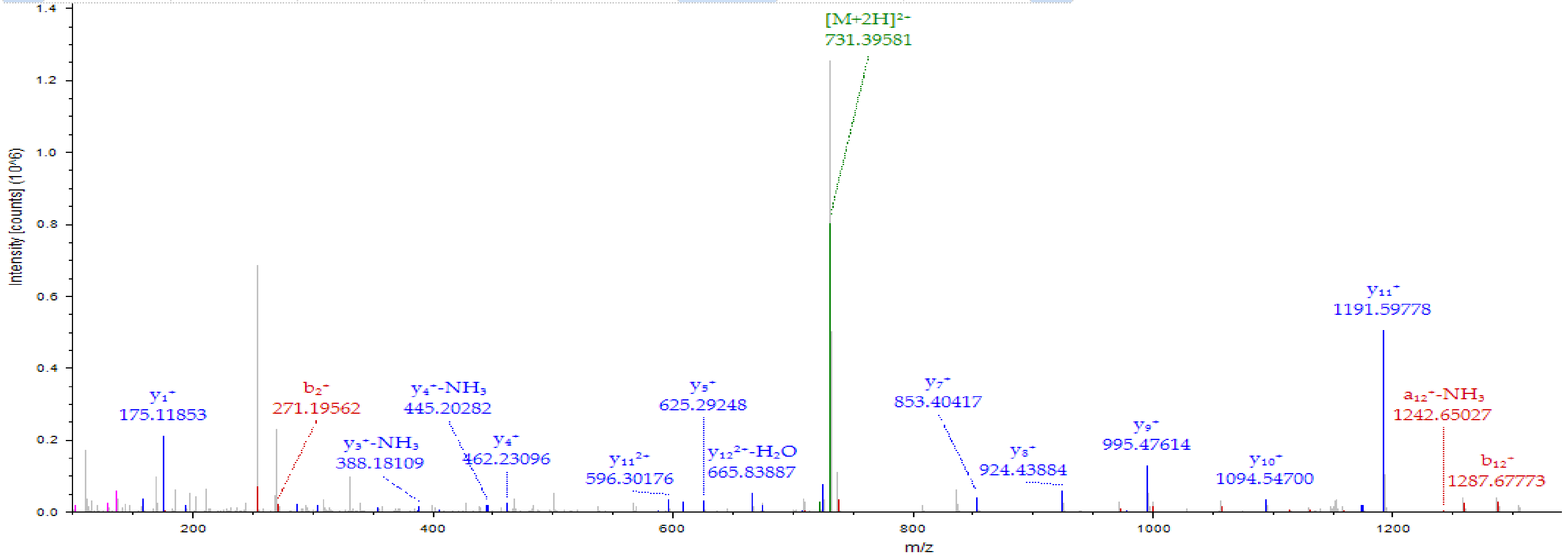

AUP sample #118

Sequence: RAAPEASGTPSSDAVSR, R1-Deamidated (0.98402 Da)
Charge: +3, Monoisotopic m/z: 553.93597 Da (+0 mmu/+0.01 ppm), MH+: 1659.79337 Da, RT: 16.93 min,
Identified with: Sequest HT (v1.3); XCorr:3.73, Ions matched by search engine: 0/0
Fragment match tolerance used for search: 0.02 Da
Fragments used for search: b; b-H_2O; b-NH_3; y; y-H_2O; y-NH_3

Protein references (1):
- Coronin-1A OS=Homo sapiens GN=CORO1A PE=1 SV=4 - [COR1A_HUMAN]

#1	Immonium	a^+	a^{2+}	b^+	b^{2+}	Seq.	y^+	y^{2+}	#2
1	129.11348	130.09750	65.55239	158.09241	79.54984	R-Deamid...			17
2	44.04948	201.13462	101.07095	229.12953	115.06840	A	1502.70822	751.85775	16
3	44.04948	272.17174	136.58951	300.16665	150.58696	A	1431.67110	716.33919	15
4	70.06513	369.22451	185.11589	397.21942	199.11335	P	1360.63398	680.82063	14
5	102.05496	498.26711	249.63719	526.26202	263.63465	E	1263.58121	632.29424	13
6	44.04948	569.30423	285.15575	597.29914	299.15321	A	1134.53861	567.77294	12
7	60.04439	656.33626	328.67177	684.33117	342.66922	S	1063.50149	532.25438	11
8	30.03383	713.35773	357.18250	741.35264	371.17996	G	976.46946	488.73837	10
9	74.06004	814.40541	407.70634	842.40032	421.70380	T	919.44799	460.22763	9
10	70.06513	911.45818	456.23273	939.45309	470.23018	P	818.40031	409.70379	8
11	60.04439	998.49021	499.74874	1026.48512	513.74620	S	721.34754	361.17741	7
12	60.04439	1085.52224	543.26476	1113.51715	557.26221	S	634.31551	317.66139	6
13	88.03931	1200.54919	600.77823	1228.54410	614.77569	D	547.28348	274.14538	5
14	44.04948	1271.58631	636.29679	1299.58122	650.29425	A	432.25653	216.63190	4
15	72.08078	1370.65473	685.83100	1398.64964	699.82846	V	361.21941	181.11334	3
16	60.04439	1457.68676	729.34702	1485.68167	743.34447	S	262.15099	131.57913	2
17						R	175.11896	88.06312	1

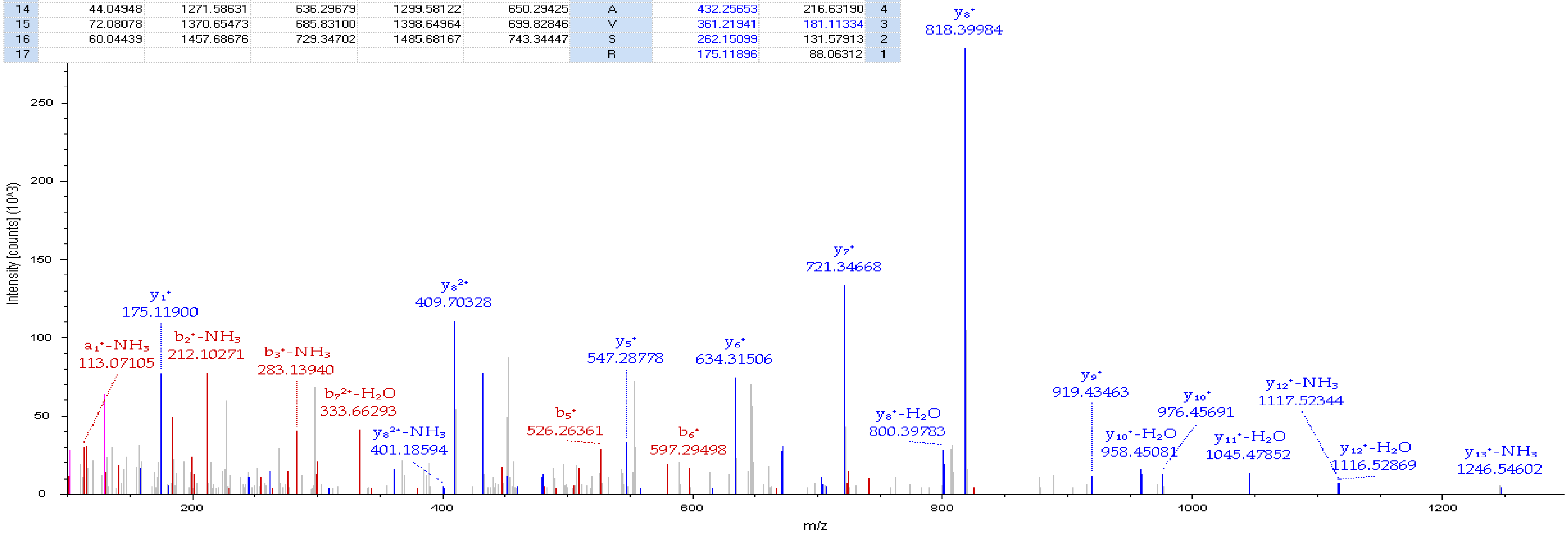

AUP sample #118

Sequence: SRLGDLYEEEMR, R2-Deamidated (0.98402 Da)
Charge: +2, Monoisotopic m/z: 749.84564 Da (-0.15 mmu/-0.2 ppm), MH+: 1498.68401 Da, RT: 51.71 min,
Identified with: Sequest HT (v1.3); XCorr:3.41, Ions matched by search engine: 0/0
Fragment match tolerance used for search: 0.02 Da
Fragments used for search: b; b-H_2O; b-NH_3; y; y-H_2O; y-NH_3

Protein references (2):
- Vimentin OS=Homo sapiens GN=VIM PE=1 SV=4 - [VIME_HUMAN]

#1	Immonium	b^+	b^{2+}	Seq.	y^+	y^{2+}	#2
1	60.04439	88.03931	44.52329	S			12
2	129.11348	245.12444	123.06586	R-Deamid...	1411.65228	706.32978	11
3	86.09643	358.20851	179.60789	L	1254.56714	627.78721	10
4	30.03383	415.22998	208.11863	G	1141.48307	571.24517	9
5	88.03931	530.25693	265.63210	D	1084.46160	542.73444	8
6	86.09643	643.34100	322.17414	L	969.43465	485.22096	7
7	136.07568	806.40432	403.70580	Y	856.35058	428.67893	6
8	102.05496	935.44692	468.22710	E	693.28726	347.14727	5
9	102.05496	1064.48952	532.74840	E	564.24466	282.62597	4
10	102.05496	1193.53212	597.26970	E	435.20206	218.10467	3
11	104.05286	1324.57262	662.78995	M	306.15946	153.58337	2
12				R	175.11896	88.06312	1

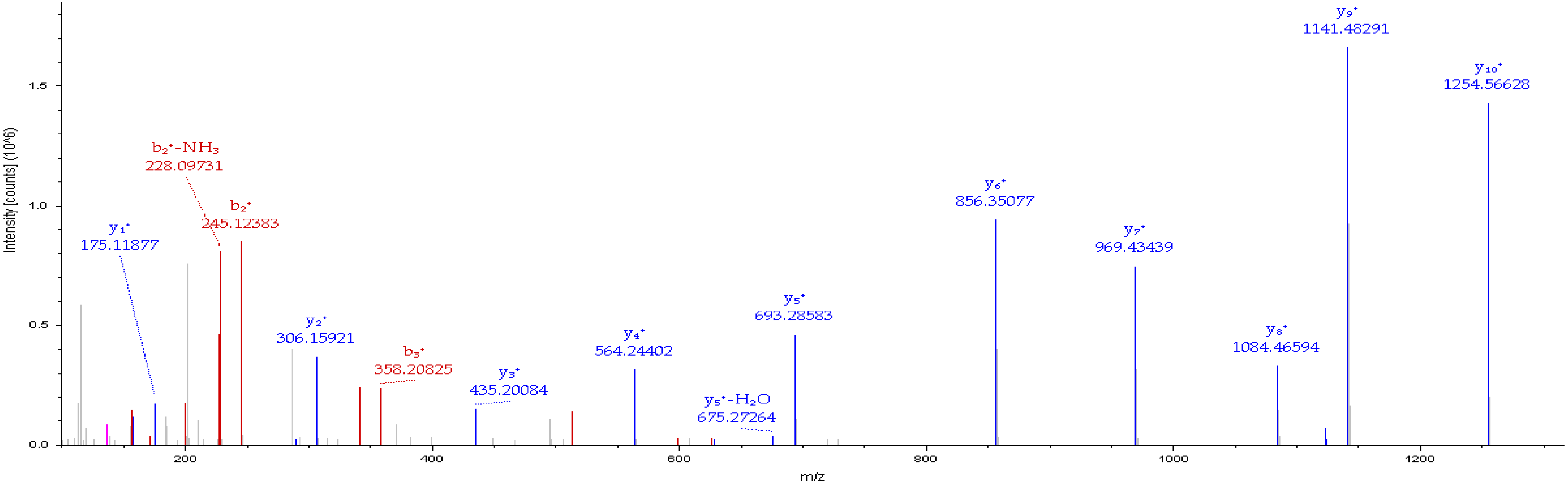

AUP sample #118

Sequence: RDLGEELEALK, R1-Deamidated (0.98402 Da)
Charge: +2, Monoisotopic m/z: 637.33472 Da (-0.67 mmu/-1.05 ppm), MH+: 1273.66216 Da, RT: 54.05 min,
Identified with: Sequest HT (v1.3); XCorr:2.80, Ions matched by search engine: 0/0
Fragment match tolerance used for search: 0.02 Da
Fragments used for search: b; b-H_2O; b-NH_3; y; y-H_2O; y-NH_3

Protein references (1):
- Myosin-9 OS=Homo sapiens GN=MYH9 PE=1 SV=4 - [MYH9_HUMAN]

#1	Immonium	a^+	a^{2+}	b^+	b^{2+}	Seq.	y^+	y^{2+}	#2
1	129.11348	130.09750	65.55239	158.09241	79.54984	R-Deamid...			11
2	88.03931	245.12445	123.06586	273.11936	137.06332	D	1116.57836	558.79282	10
3	86.09643	358.20852	179.60790	386.20343	193.60535	L	1001.55141	501.27934	9
4	30.03383	415.22999	208.11863	443.22490	222.11609	G	888.46734	444.73731	8
5	102.05496	544.27259	272.63993	572.26750	286.63739	E	831.44587	416.22657	7
6	102.05496	673.31519	337.16123	701.31010	351.15869	E	702.40327	351.70527	6
7	86.09643	786.39926	393.70327	814.39417	407.70072	L	573.36067	287.18397	5
8	102.05496	915.44186	458.22457	943.43677	472.22202	E	460.27660	230.64194	4
9	44.04948	986.47898	493.74313	1014.47389	507.74058	A	331.23400	166.12064	3
10	86.09643	1099.56305	550.28516	1127.55796	564.28262	L	260.19688	130.60208	2
11						K	147.11281	74.06004	1

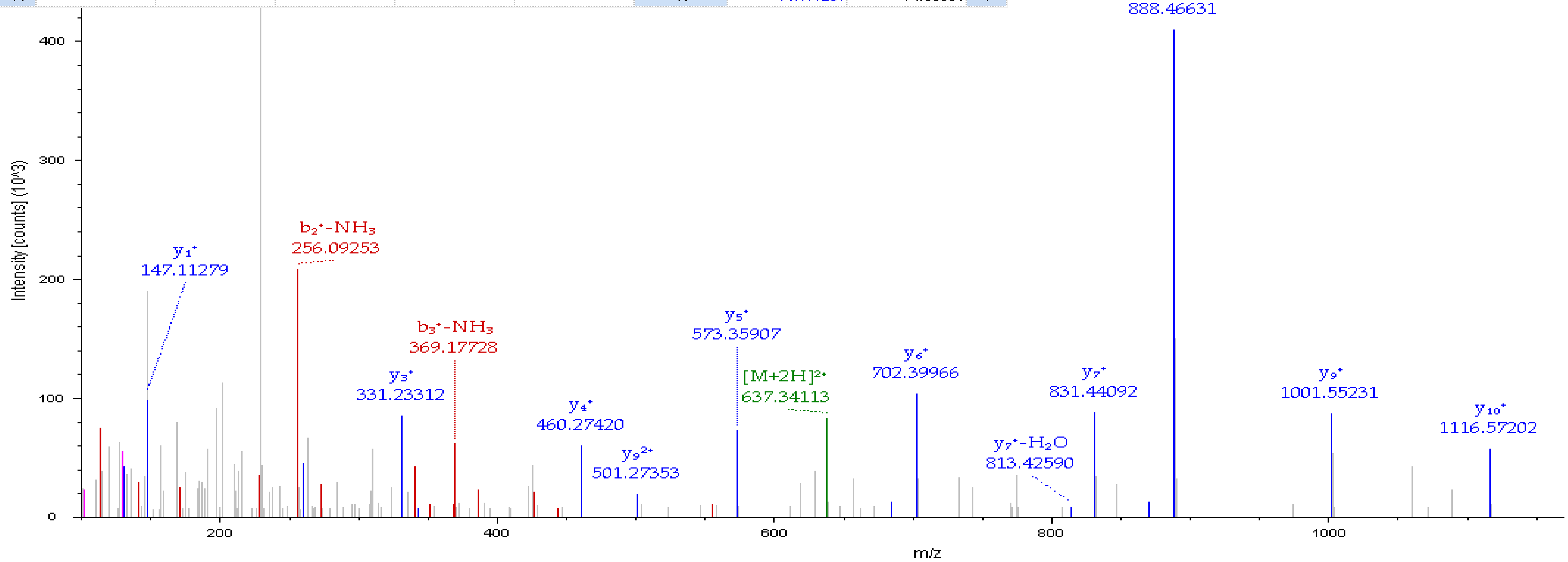

AUP sample #118

Sequence: SRMGPSGGEGMEPER, R2-Deamidated (0.98402 Da)
Charge: +2, Monoisotopic m/z: 789.33606 Da (-1.77 mmu/-2.24 ppm), MH+: 1577.66484 Da, RT: 21.47 min,
Identified with: Sequest HT (v1.3); XCorr:1.98, Ions matched by search engine: 0/0
Fragment match tolerance used for search: 0.02 Da
Fragments used for search: b; b-H_2O; b-NH_3; y; y-H_2O; y-NH_3

Protein references (1):
- LIM and SH3 domain protein 1 OS=Homo sapiens GN=LASP1 PE=2 SV=1 - [B4DGQ0_HUMAN]

#1	Immonium	a^+	a^{2+}	b^+	b^{2+}	Seq.	y^+	y^{2+}	#2
1	60.04439	60.04439	30.52583	88.03931	44.52329	S			15
2	129.11348	217.12953	109.06840	245.12444	123.06586	R-Deamid...	1490.63635	745.82181	14
3	104.05286	348.17003	174.58865	376.16494	188.58611	M	1333.55121	667.27924	13
4	30.03383	405.19150	203.09939	433.18641	217.09684	G	1202.51071	601.75899	12
5	70.06513	502.24427	251.62577	530.23918	265.62323	P	1145.48924	573.24826	11
6	60.04439	589.27630	295.14179	617.27121	309.13924	S	1048.43647	524.72187	10
7	30.03383	646.29777	323.65252	674.29268	337.64998	G	961.40444	481.20586	9
8	30.03383	703.31924	352.16326	731.31415	366.16071	G	904.38297	452.69512	8
9	102.05496	832.36184	416.68456	860.35675	430.68201	E	847.36150	424.18439	7
10	30.03383	889.38331	445.19529	917.37822	459.19275	G	718.31890	359.66309	6
11	104.05286	1020.42381	510.71554	1048.41872	524.71300	M	661.29743	331.15235	5
12	102.05496	1149.46641	575.23684	1177.46132	589.23430	E	530.25693	265.63210	4
13	70.06513	1246.51918	623.76323	1274.51409	637.76068	P	401.21433	201.11080	3
14	102.05496	1375.56178	688.28453	1403.55669	702.28198	E	304.16156	152.58442	2
15						R	175.11896	88.06312	1

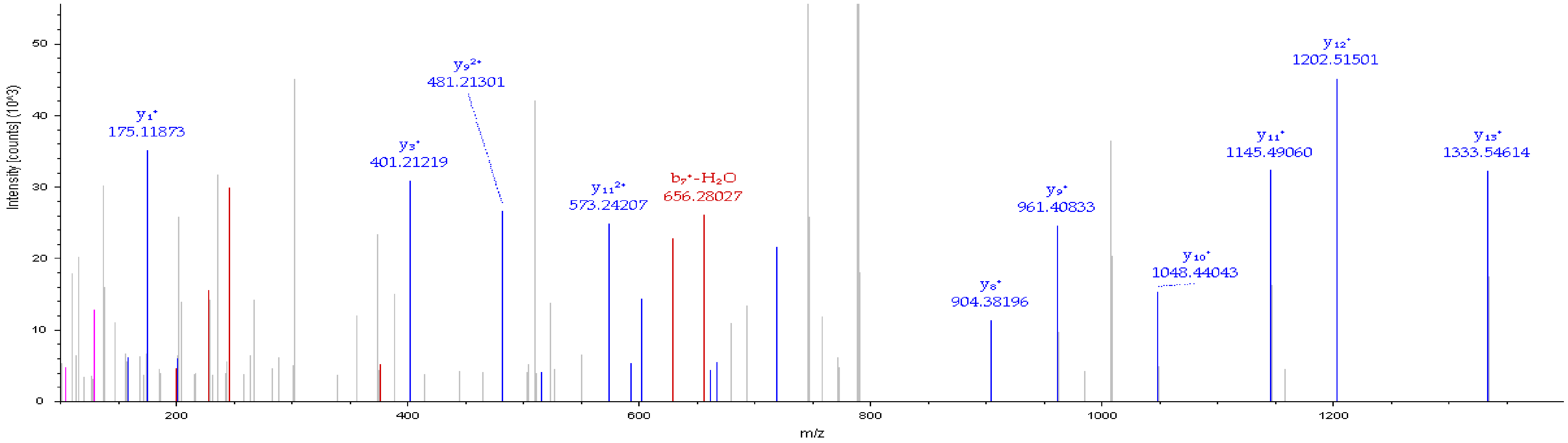

AUP sample #118

Sequence: SRMGPSGGEGMEPER, R2-Deamidated (0.98402 Da), M3-Oxidation (15.99492 Da)
Charge: +2, Monoisotopic m/z: 797.33661 Da (+1.33 mmu/+1.66 ppm), MH+: 1593.66594 Da, RT: 13.71 min,
Identified with: Sequest HT (v1.3); XCorr:2.62, Ions matched by search engine: 0/0
Fragment match tolerance used for search: 0.02 Da
Fragments used for search: b; b-H_2O; b-NH_3; y; y-H_2O; y-NH_3

Protein references (1):
- LIM and SH3 domain protein 1 OS=Homo sapiens GN=LASP1 PE=1 SV=2 - [LASP1_HUMAN]

#1	Immonium	a^+	a^{2+}	b^+	b^{2+}	Seq.	y^+	y^{2+}	#2
1	60.04439	60.04439	30.52583	88.03931	44.52329	S			15
2	129.11348	217.12953	109.06840	245.12444	123.06586	R-Deamid...	1506.63126	753.81927	14
3	104.05286	364.16494	182.58611	392.15986	196.58357	M-Oxidation	1349.54613	675.27670	13
4	30.03383	421.18641	211.09684	449.18133	225.09430	G	1202.51071	601.75899	12
5	70.06513	518.23918	259.62323	546.23410	273.62069	P	1145.48924	573.24826	11
6	60.04439	605.27121	303.13924	633.26613	317.13670	S	1048.43647	524.72187	10
7	30.03383	662.29268	331.64998	690.28760	345.64744	G	961.40444	481.20586	9
8	30.03383	719.31415	360.16071	747.30907	374.15817	G	904.38297	452.69512	8
9	102.05496	848.35675	424.68201	876.35167	438.67947	E	847.36150	424.18439	7
10	30.03383	905.37822	453.19275	933.37314	467.19021	G	718.31890	359.66309	6
11	104.05286	1036.41872	518.71300	1064.41364	532.71046	M	661.29743	331.15235	5
12	102.05496	1165.46132	583.23430	1193.45624	597.23176	E	530.25693	265.63210	4
13	70.06513	1262.51409	631.76068	1290.50901	645.75814	P	401.21433	201.11080	3
14	102.05496	1391.55669	696.28198	1419.55161	710.27944	E	304.16156	152.58442	2
15						R	175.11896	88.06312	1

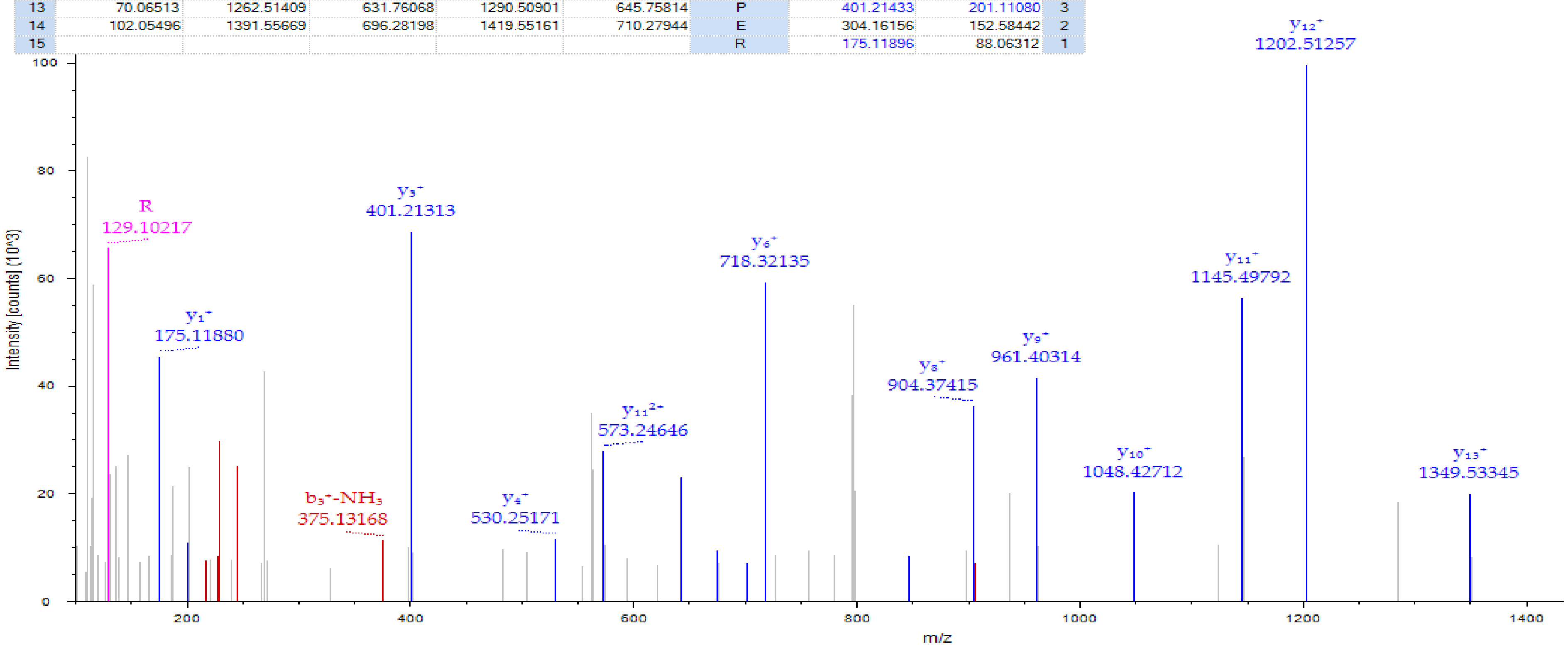

AUP sample #118

Sequence: RGPPPPPR, R1-Deamidated (0.98402 Da)
Charge: +2, Monoisotopic m/z: 437.74869 Da (+0.34 mmu/+0.78 ppm), MH+: 874.49010 Da, RT: 11.77 min,
Identified with: Sequest HT (v1.3); XCorr:1.51, Ions matched by search engine: 0/0
Fragment match tolerance used for search: 0.02 Da
Fragments used for search: b; b-NH_3; y; y-NH_3

Protein references (4):
- RNA-binding motif protein, X chromosome OS=Homo sapiens GN=RBMX PE=1 SV=3 - [RBMX_HUMAN]

#1	Immonium	a^+	a^{2+}	b^+	b^{2+}	Seq.	y^+	y^{2+}	#2
1	129.11348	130.09750	65.55239	158.09241	79.54984	R-Deamid...			8
2	30.03383	187.11897	94.06312	215.11388	108.06058	G	717.40428	359.20578	7
3	70.06513	284.17174	142.58951	312.16665	156.58696	P	660.38281	330.69504	6
4	70.06513	381.22451	191.11589	409.21942	205.11335	P	563.33004	282.16866	5
5	70.06513	478.27728	239.64228	506.27219	253.63973	P	466.27727	233.64227	4
6	70.06513	575.33005	288.16866	603.32496	302.16612	P	369.22450	185.11589	3
7	70.06513	672.38282	336.69505	700.37773	350.69250	P	272.17173	136.58950	2
8						R	175.11896	88.06312	1

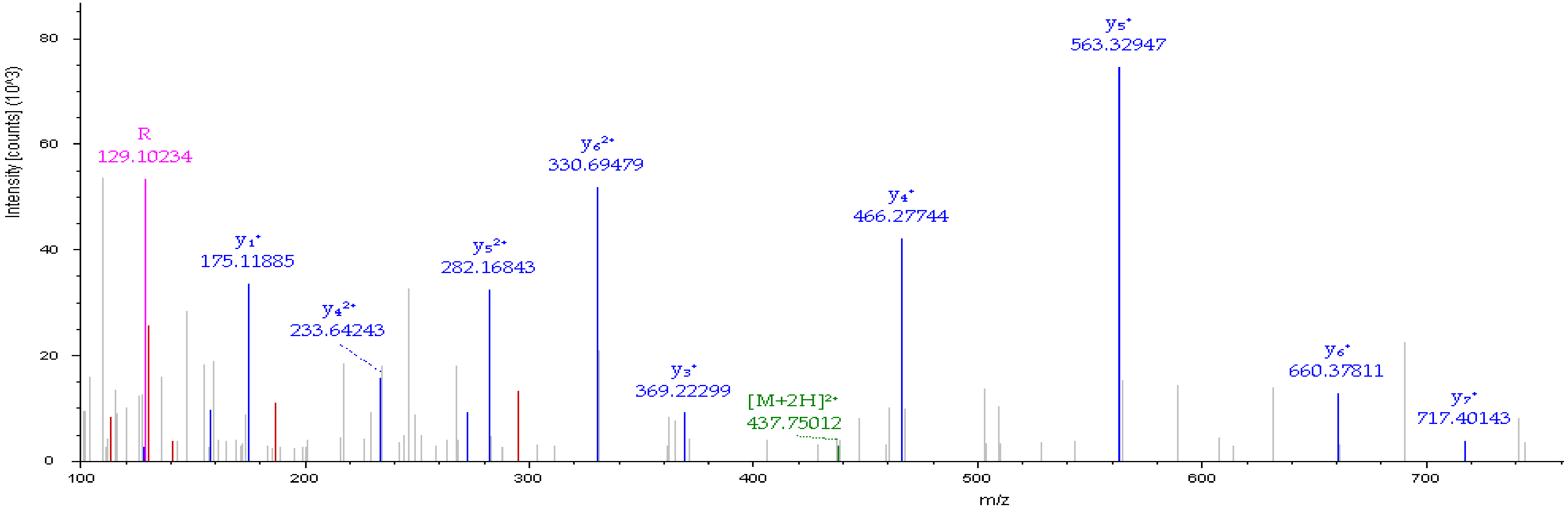

AUP sample #118

Sequence: RDYDDMSPR, R1-Deamidated (0.98402 Da)
Charge: +2, Monoisotopic m/z: 578.24115 Da (+0.73 mmu/+1.26 ppm), MH+: 1155.47502 Da, RT: 19.75 min,
Identified with: Sequest HT (v1.3); XCorr:2.18, Ions matched by search engine: 0/0
Fragment match tolerance used for search: 0.02 Da
Fragments used for search: b; b-H_2O; b-NH_3; y; y-H_2O; y-NH_3

Protein references (1):
- Heterogeneous nuclear ribonucleoprotein K OS=Homo sapiens GN=HNRNPK PE=1 SV=1 - [HNRPK_HUMAN]

#1	Immonium	b^+	b^{2+}	Seq.	y^+	y^{2+}	#2
1	129.11348	158.09241	79.54984	R-Deamid...			9
2	88.03931	273.11936	137.06332	D	998.38843	499.69785	8
3	136.07568	436.18268	218.59498	Y	883.36148	442.18438	7
4	88.03931	551.20963	276.10845	D	720.29816	360.65272	6
5	88.03931	666.23658	333.62193	D	605.27121	303.13924	5
6	104.05286	797.27708	399.14218	M	490.24426	245.62577	4
7	60.04439	884.30911	442.65819	S	359.20376	180.10552	3
8	70.06513	981.36188	491.18458	P	272.17173	136.58950	2
9				R	175.11896	88.06312	1

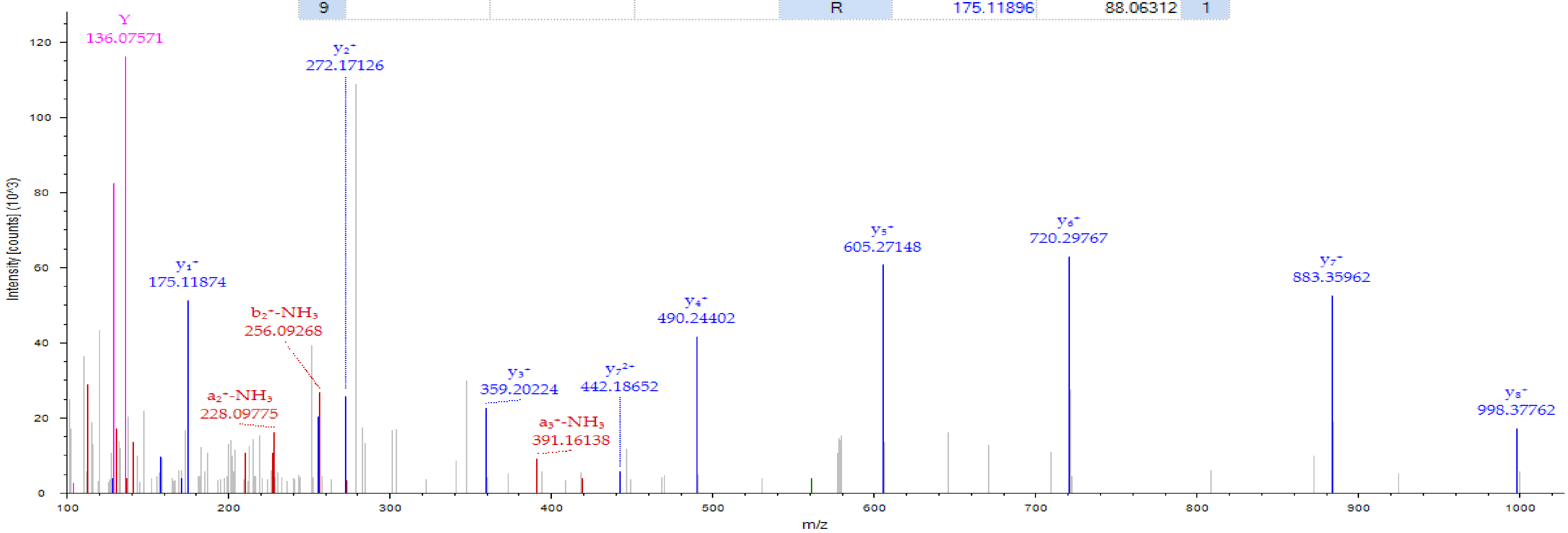

AUP sample #118

Sequence: REEDEPEER, R1-Deamidated (0.98402 Da)
Charge: +2, Monoisotopic m/z: 595.25061 Da (-1.44 mmu/-2.42 ppm), MH+: 1189.49394 Da, RT: 9.29 min,
Identified with: Sequest HT (v1.3); XCorr:2.07, Ions matched by search engine: 0/0
Fragment match tolerance used for search: 0.02 Da
Fragments used for search: b; b-H_2O; b-NH_3; y; y-H_2O; y-NH_3

Protein references (1):
- Heterogeneous nuclear ribonucleoprotein U-like protein 2 OS=Homo sapiens GN=HNRNPUL2 PE=1 SV=1 - [HNRL2_HUMAN]

#1	Immonium	a^+	a^{2+}	b^+	b^{2+}	Seq.	y^+	y^{2+}	#2
1	129.11348	130.09750	65.55239	158.09241	79.54984	R-Deamid...			9
2	102.05496	259.14010	130.07369	287.13501	144.07114	E	1032.41168	516.70948	8
3	102.05496	388.18270	194.59499	416.17761	208.59244	E	903.36908	452.18818	7
4	88.03931	503.20965	252.10846	531.20456	266.10592	D	774.32648	387.66688	6
5	102.05496	632.25225	316.62976	660.24716	330.62722	E	659.29953	330.15340	5
6	70.06513	729.30502	365.15615	757.29993	379.15360	P	530.25693	265.63210	4
7	102.05496	858.34762	429.67745	886.34253	443.67490	E	433.20416	217.10572	3
8	102.05496	987.39022	494.19875	1015.38513	508.19620	E	304.16156	152.58442	2
9						R	175.11896	88.06312	1

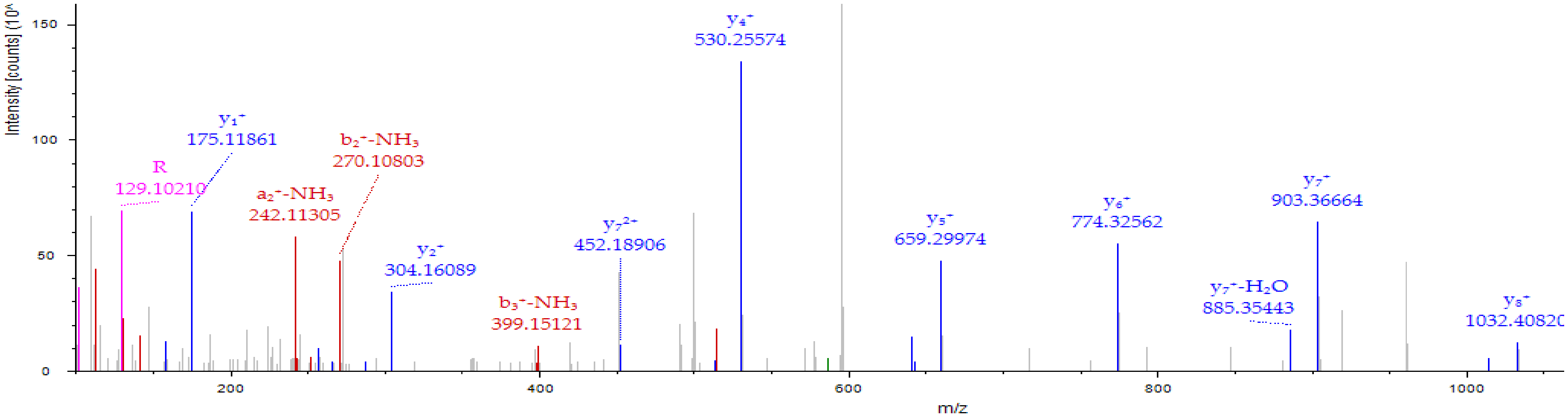

AUP sample #118

Sequence: KREPEDEGEDDD, R2-Deamidated (0.98402 Da)
Charge: +2, Monoisotopic m/z: 717.77820 Da (-0.63 mmu/-0.88 ppm), MH+: 1434.54912 Da, RT: 9.11 min,
Identified with: Sequest HT (v1.3); XCorr:2.38, Ions matched by search engine: 0/0
Fragment match tolerance used for search: 0.02 Da
Fragments used for search: b; b-H_2O; b-NH_3; y; y-H_2O; y-NH_3

Protein references (1):
- Acidic leucine-rich nuclear phosphoprotein 32 family member A OS=Homo sapiens GN=ANP32A PE=1 SV=1 - [AN32A_HUMAN]

#1	b^+	Seq.	y^+	#2
1	129.10225	K		12
2	286.18738	R-Deamid...	1306.45542	11
3	415.22998	E	1149.37028	10
4	512.28275	P	1020.32768	9
5	641.32535	E	923.27491	8
6	756.35230	D	794.23231	7
7	885.39490	E	679.20536	6
8	942.41637	G	550.16276	5
9	1071.45897	E	493.14129	4
10	1186.48592	D	364.09869	3
11	1301.51287	D	249.07174	2
12		D	134.04479	1

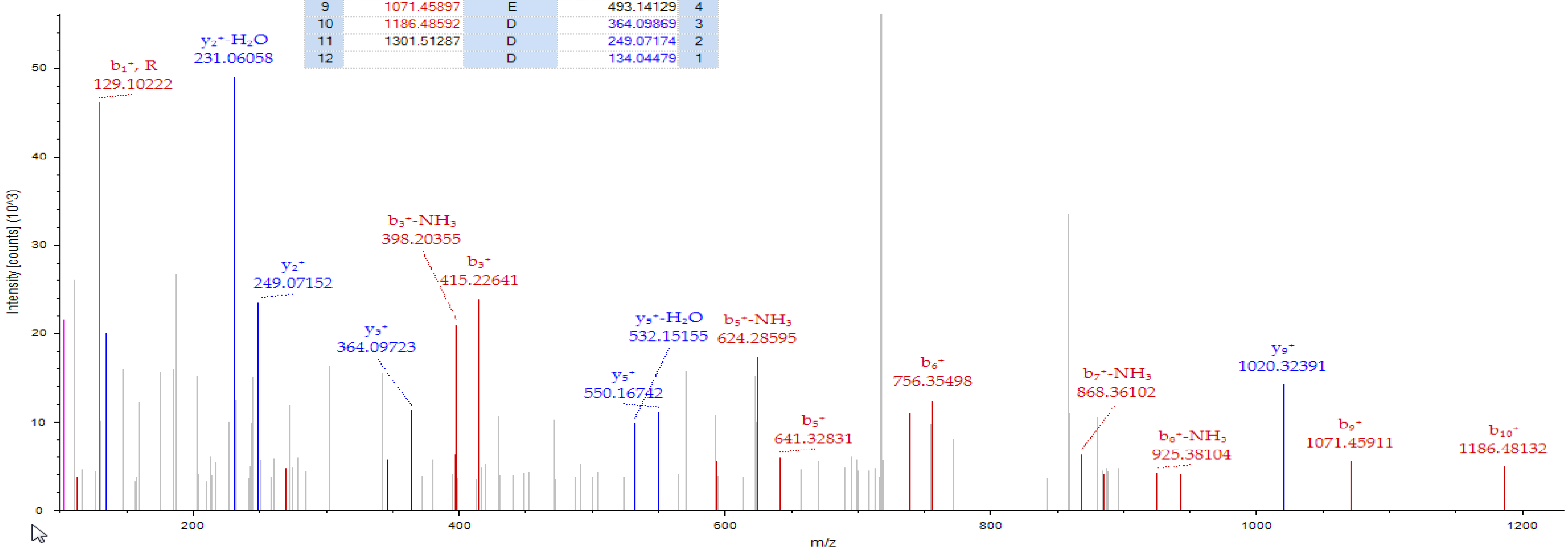

DUP sample #2

Sequence: ILTE**R(196)**GYSFTTTAER, R5-Deamidated (0.98402 Da)
Charge: +2, Monoisotopic m/z: 873.44525 Da (+6.36 mmu/+7.28 ppm), MH+: 1745.88323 Da, RT: 54.08 min,
Identified with: Sequest HT (v1.3); XCorr:3.44, Ions matched by search engine: 0/0
Fragment match tolerance used for search: 0.02 Da
Fragments used for search: b; b-H_2O; b-NH_3; y; y-H_2O; y-NH_3

Protein references (1):
- Actin, cytoplasmic 1 OS=Homo sapiens GN=ACTB PE=1 SV=1 - [ACTB_HUMAN]

#1	Immonium	a^+	a^{2+}	b^+	b^{2+}	Seq.	y^+	y^{2+}	#2
1	86.09643	86.09643	43.55185	114.09135	57.54931	I			15
2	86.09643	199.18050	100.09389	227.17542	114.09135	L	1632.78645	816.89686	14
3	74.06004	300.22818	150.61773	328.22310	164.61519	T	1519.70238	760.35483	13
4	102.05496	429.27078	215.13903	457.26570	229.13649	E	1418.65470	709.83099	12
5	129.11348	586.35592	293.68160	614.35083	307.67905	R-Deamid...	1289.61210	645.30969	11
6	30.03383	643.37739	322.19233	671.37230	336.18979	G	1132.52696	566.76712	10
7	136.07568	806.44071	403.72399	834.43562	417.72145	Y	1075.50549	538.25638	9
8	60.04439	893.47274	447.24001	921.46765	461.23746	S	912.44217	456.72472	8
9	120.08078	1040.54116	520.77422	1068.53607	534.77167	F	825.41014	413.20871	7
10	74.06004	1141.58884	571.29806	1169.58375	585.29551	T	678.34172	339.67450	6
11	74.06004	1242.63652	621.82190	1270.63143	635.81935	T	577.29404	289.15066	5
12	74.06004	1343.68420	672.34574	1371.67911	686.34319	T	476.24636	238.62682	4
13	44.04948	1414.72132	707.86430	1442.71623	721.86175	A	375.19868	188.10298	3
14	102.05496	1543.76392	772.38560	1571.75883	786.38305	E	304.16156	152.58442	2
15						R	175.11896	88.06312	1

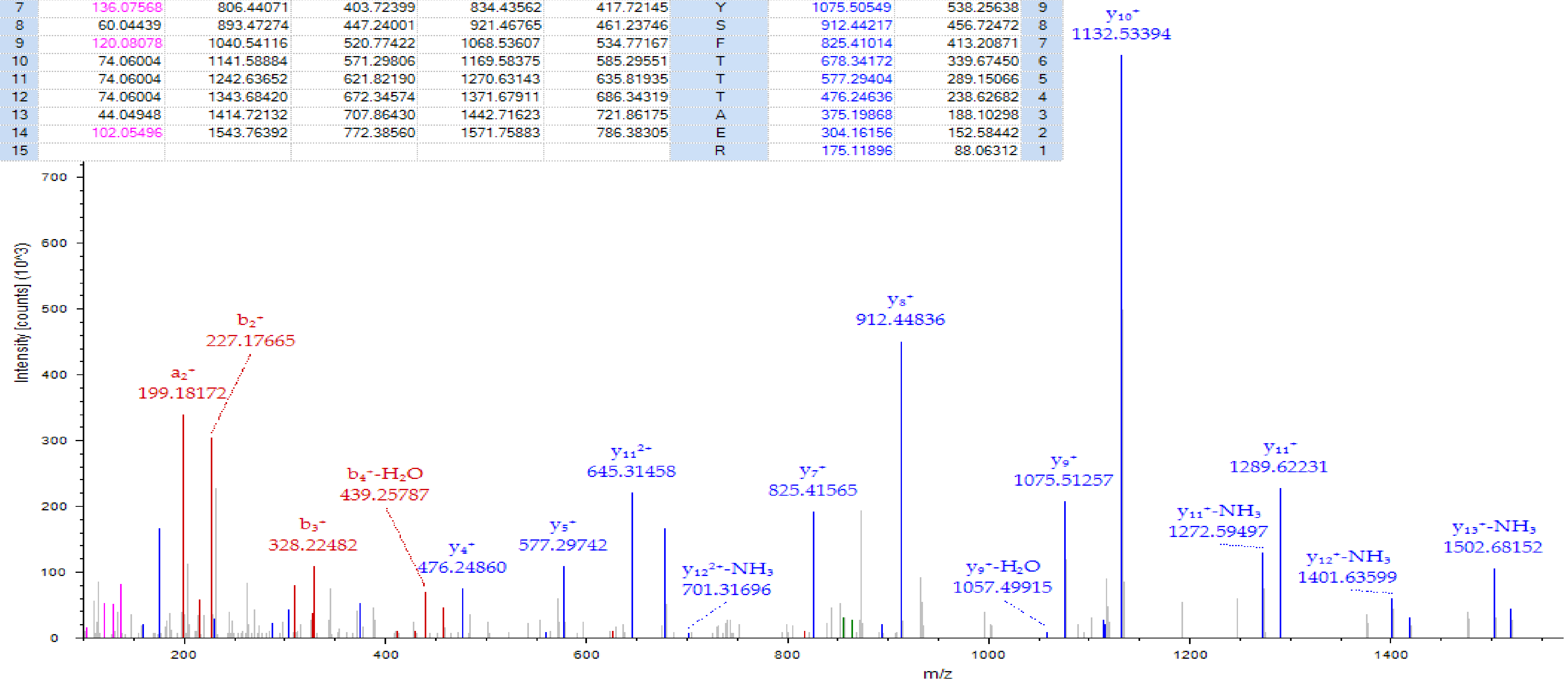

DUP sample #2

Sequence: ILTER(196)GYSFTTTAER(206)EIVR(210)DIK, R5-Deamidated (0.98402 Da), R15-Deamidated (0.98402 Da), R19-Deamidated (0.98402 Da)
Charge: +3, Monoisotopic m/z: 867.79004 Da (+4.94 mmu/+5.69 ppm), MH+: 2601.35556 Da, RT: 86.96 min,
Identified with: Sequest HT (v1.3); XCorr:3.69, Ions matched by search engine: 0/0
Fragment match tolerance used for search: 0.02 Da
Fragments used for search: b; b-H_2O; b-NH_3; y; y-H_2O; y-NH_3

Protein references (1):
- Actin, cytoplasmic 1 OS=Homo sapiens GN=ACTB PE=1 SV=1 - [ACTB_HUMAN]

#1	Immonium	a+	a2+	b+	b2+	Seq.	y+	y2+	#2
1	86.09643	86.09643	43.55185	114.09135	57.54931	I			22
2	86.09643	199.18050	100.09389	227.17542	114.09135	L	2488.25668	1244.63198	21
3	74.06004	300.22818	150.61773	328.22310	164.61519	T	2375.17261	1188.08994	20
4	102.05496	429.27078	215.13903	457.26570	229.13649	E	2274.12493	1137.56610	19
5	129.11348	586.35592	293.68160	614.35083	307.67905	R-Deamid...	2145.08233	1073.04480	18
6	30.03383	643.37739	322.19233	671.37230	336.18979	G	1987.99719	994.50223	17
7	136.07568	806.44071	403.72399	834.43562	417.72145	Y	1930.97572	965.99150	16
8	60.04439	893.47274	447.24001	921.46765	461.23746	S	1767.91240	884.45984	15
9	120.08078	1040.54116	520.77422	1068.53607	534.77167	F	1680.88037	840.94382	14
10	74.06004	1141.58884	571.29806	1169.58375	585.29551	T	1533.81195	767.40961	13
11	74.06004	1242.63652	621.82190	1270.63143	635.81935	T	1432.76427	716.88577	12
12	74.06004	1343.68420	672.34574	1371.67911	686.34319	T	1331.71659	666.36193	11
13	44.04948	1414.72132	707.86430	1442.71623	721.86175	A	1230.66891	615.83809	10
14	102.05496	1543.76392	772.38560	1571.75883	786.38305	E	1159.63179	580.31953	9
15	129.11348	1700.84905	850.92817	1728.84397	864.92562	R-Deamid...	1030.58919	515.79823	8
16	102.05496	1829.89165	915.44947	1857.88657	929.44692	E	873.50406	437.25567	7
17	86.09643	1942.97572	971.99150	1970.97064	985.98896	I	744.46146	372.73437	6
18	72.08078	2042.04414	1021.52571	2070.03906	1035.52317	V	631.37739	316.19233	5
19	129.11348	2199.12928	1100.06828	2227.12419	1114.06574	R-Deamid...	532.30897	266.65812	4
20	88.03931	2314.15623	1157.58175	2342.15114	1171.57921	D	375.22383	188.11555	3
21	86.09643	2427.24030	1214.12379	2455.23521	1228.12125	I	260.19688	130.60208	2
22						K	147.11281	74.06004	1

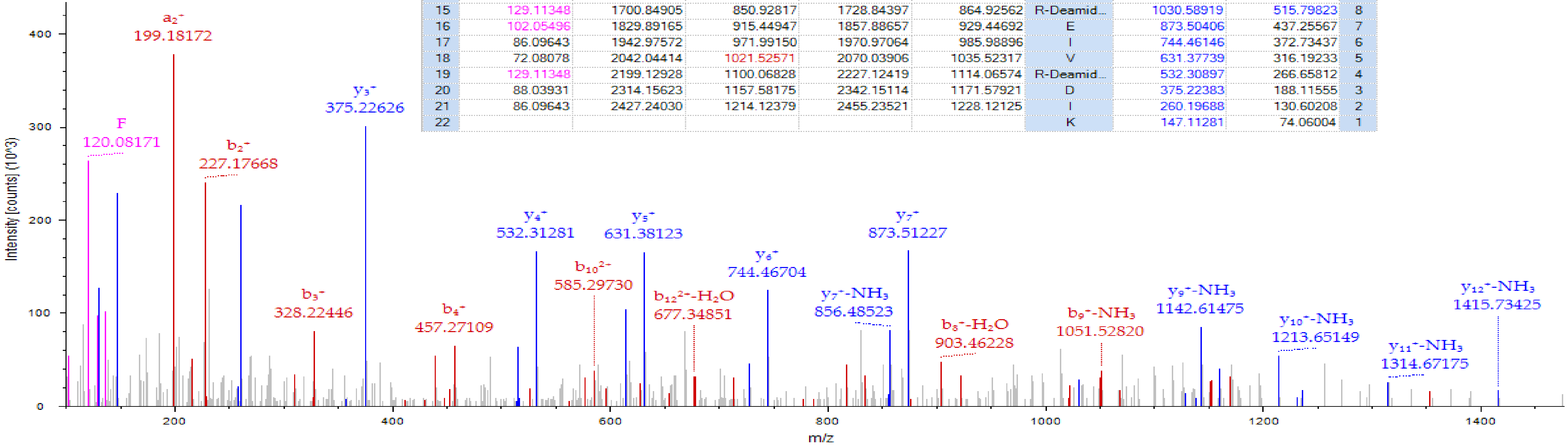

Supplement: S4 Data — (PDF) [file ppat.1006151.s004.pdf]
